# The global intellectual property ecosystem for insulin and its public health implications: an observational study

**DOI:** 10.1186/s40545-016-0072-8

**Published:** 2016-07-19

**Authors:** Warren A. Kaplan, Reed F. Beall

**Affiliations:** Department of Global Health, Boston University School of Public Health, Boston, MA 02118 USA; Population Health Program, Faculties of Medicine and of Law, University of Ottawa, One Stewart St, Ottawa, ON K1N 6N5 Canada

**Keywords:** Patent, Intellectual property, Pharmaceutical policy, Generic medicine

## Abstract

**Background:**

Lack of access to insulin and poor health outcomes are issues for both low and high income countries. This has been accompanied by a shift from relatively inexpensive human insulin to its more expensive analogs, marketed by three to four main global players. Nonetheless, patent-based market exclusivities are beginning to expire there for the first generation insulin analogs. This paper adds a global dimension to information on the U.S. patent landscape for insulin by reviewing the patent status of insulins with emphasis on the situation outside the US and Europe.

**Methods:**

Using the term “insulin”, we searched for patents listed on the United States Food and Drug Administration’s (USFDA) Orange Book and the Canadian Online Drug Product Database Online Query and its Patent Register. With this information, we expanded the search globally using the World Intellectual Property Organization (WIPO) PatentScope database, the European Patent Office’s INPADOC database and various country-specific Patent Offices.

**Results:**

Patent protected insulins marketed in the U.S. and other countries are facing an imminent patent-expiration “cliff’ yet the three companies that dominate the global insulin market are continuing to file for patents in and outside the U.S, but very rarely in Africa. Only a few local producers in the so-called "pharmerging" markets (e.g., Brazil, India, China) are filing for global patent protection on their own insulins. There is moderate, but statistically significant association between patent filings and diabetes disease burden.

**Conclusions:**

The global market dominance by a few companies of analog over human insulin will likely continue even though patents on the current portfolio of insulin analogs will expire very soon. Multinationals are continuing to file for more insulin patents in the bigger markets with large disease burdens and a rapidly emerging middle class. Off-patent human insulins can effectively manage diabetes. A practical way forward would be find (potential) generic manufacturers globally and nudge them towards opportunities to diversify their national insulin markets with acceptable off-patent products for export.

**Electronic supplementary material:**

The online version of this article (doi:10.1186/s40545-016-0072-8) contains supplementary material, which is available to authorized users.

## Background

The disease burden of diabetes has been steadily rising and improving access to insulin, long considered an "essential medicine" by many countries as well as the World Health Organization (WHO) [1], has taken on increasing importance [2]. Essential medicines satisfy the priority health care needs of societies and are considered as a basis for public procurement or reimbursement decisions, yet fully one third of the world’s population currently has no guaranteed access to essential medicines [3]. More than 2 billion people in low and middle income (LMIC) countries face significant barriers in accessing basic health services. Nevertheless, the challenge of access to essential medicines is not limited to low and middle income countries [4].

A recent situational review of global insulin access [5] notes that although insulin was discovered in 1921, the drug is unattainable to many globally. There is a wide range and complexity of factors that contribute to this unattainability. This review noted that “… little has been done globally to address the issue of access, despite the UN’s political commitment to address non-communicable diseases and ensure universal access to drugs for these disorders.” Lack of access to insulin is a common issue in the United States [6] and Europe [7]. Insulin sales in the USA for 2011 totalled US$8.3 billion, a 14.9 % increase compared with 2010 and U.S. government reimbursement costs for insulin have been steadily rising as well, complicating access to this vital therapeutic to un- and under-insured populations [8]. Between 1991 and 2014, there was a near-exponential upward trend in Medicaid payments on a per-unit basis for a wide variety of insulin products regardless of formulation, duration of action, and whether the product was patented [8]. It has been almost a century since the first patient was treated with insulin and recombinant human insulin has been off-patent around the world for a decade and a half [9, 10]. Yet reimbursements for newer, patent-protected insulin analogs increased at a faster rate than reimbursements for older insulins [8], and older porcine- and bovine insulin products are no longer available on the American market. We note that manufacturing of beef insulin for human use in the U.S. was discontinued in 1998 as was the manufacturing of pork insulin (Iletin II) for human use in 2006. According to the U.S. Food and Drug Admininstration (FDA) discontinuation of animal-sourced insulins was a voluntary withdrawal of these products made by the manufacturers and not based on any FDA regulatory action [11].

All this has been accompanied by a shift from human insulin to its analogs, marketed by three or four main global players [12]. In 2000, 86.3 % of insulin used in the UK was human and 10.7 % analog insulins. By 2008, however, the use of human insulin had fallen to 23.2 %, with analogs representing 76.1 % of the total [5]. This trend toward increasing use of insulin analogues is occurring despite a 2011 World Health Organization (WHO) report which asserted while many comparative clinical trials “… find a statistically significant difference between analogue insulins and standard recombinant human insulin for some blood glucose measurements, there is no evidence of a clinically significant difference in most outcomes” [5]. We will not speculate as to whether this move towards analogue insulin was motivated by better clinical outcomes or by commercial and marketing interests [13].

This paper adds a global dimension to the previous information on the U.S. patent ‘landscape’ for insulin [9]. We review the patent status of insulin from a public health lens with emphasis on the situation outside the US and Europe. In a recent study of national Essential Medicines Lists [1], six of 32 countries (19 %) had selected insulin analogs as essential medicines, all of which were amongst the upper middle income countries and predominantly from the region of the Americas (4 out of 6 countries).

We show that while the present suite of marketed insulins has already expired- or will soon expire- globally (the so-called insulin patent-expiration “cliff’) the companies that dominate the global market are continuing to file for insulin patents in and outside the U.S, albeit rarely in Africa. We further show that only a few manufacturers in the "pharmerging" markets (e.g., Brazil, India, China) are filing for global patent protection on their own insulins. We then discuss the possible implications of this intellectual property (IP) global ecosystem for access to insulin.

## Methods

### Patents

Using the term “insulin”, we searched the United States Food and Drug Administration’s (USFDA) Orange Book [14] (OB). Companies with marketed products in the US are required by law to list each of their patents protecting “… the drug or a method of using the drug… with respect to which a claim of patent infringement could reasonably be asserted if a person not licensed by the owner of the patent engaged in the manufacture, use, or sale of the drug product” [15]. Companies with medicines on the Canadian market are similarly required to list patents associated with their marketed products with Health Canada (HC) [16–18]. As others have focused on the insulin landscape in the United States [9], we also collected data in Canada to further diversify our product and patent datasets. We relied on the Orange Book [14] and Canada’s Drug Product Database Online Query [16] for our list of marketed insulin products, regardless of patent status. Luo and Kesselheim [9] consulted the U.S. Patent Office database to locate other US products that may have not been included in the Orange Book. Their product list was the same as ours and our respective Orange Book patent lists were identical. See Additional file 1 for the list of products (INN and proprietary name) included for the present analysis.

We also checked the DrugBank website [19] which contains a historical log of patents that have been previously disclosed in the US or Canada in order to capture important additional patents that may have expired in the United States, but might not have expired elsewhere.

We then sorted these data by the supplier company (e.g., Sanofi, Novo Nordisk, Eli Lilly, Pfizer) and then by the type of insulin (i.e., human or analog). The term “insulin” provided a better retrieval of relevant patents than “analog” or any combination of these two terms (see Additional file 2).

Since the Orange Book and Health Canada databases do not contain, for example, process patents or patents for insulins that are not approved for marketing (i.e., under development), we undertook a supplemental search using several free, public patent databases. We briefly note that the European Patent Office (EPO) and World Intellectual Property Organization (WIPO) facilitate patent procedures and communications on a global or regional level. These organizations have the most official and complete information on applications as well as adjunct information. There are well over 100 countries with a patent office [20] who will have their own website with patent information but not all have the ability to search for patents online. There are many commercial and other third party patent databases, not used in this study, except as otherwise noted.

Our first patent search used the WIPO PatentScope database [21]. Although there are no globally-applicable patents, WIPO keeps record of the nearly global patent application system. We searched WIPO PatentScope for patent publications containing the word “insulin” on the cover page, with a filing date more recent than 1 January 1994, and that were submitted by the four insulin suppliers identified during the previous phase of the project, namely, Eli Lilly, Pfizer, Novo Nordisk, and Sanofi Aventis. We documented all results found in WIPO PatentScope in the same fashion as for the OB and HC.

Further, we consulted the EPO's International Patent Documentation (INPADOC) database. INPADOC is publicly available, has bibliographic information from over 95 countries and provides information about patent families, i.e. corresponding patent applications, i.e., patent applications in different countries which claim the same first filing date and which normally disclose the same invention. It also provides information concerning the legal status of patent applications and patents in those countries which report status changes [22]. We input all of our starting OB/HC and WIPO publication numbers and retrieved a list of related patent publications from around the world by pulling the entire INPADOC extended patent families (a group of related patents internationally) that were connected to our starting patent data from the United States and Canada [23]. We chose INPADOC for retrieving our international data because it is a free source, which is important for reproducibility. As mentioned above, “premium” international patent databases such as Derwent exist [24], as well as enhanced premium versions of INPADOC, such as LexisNexis Total Patent, Thomson Innovation, and Delphion [22]). There were no patents from India in our results, and so we undertook a supplementary search with the Indian Patent Office directly for patent applications and issued patents filed by these companies [25].

We were careful to group the output data by the starting patent publication, as this allowed us to clearly trace each patent publication to a marketed product by one of the four suppliers in the North American market or to a publication found in WIPO PatentScope. INPADOC returns patent publication threads. A thread starts from a single patent application filing and may include multiple legal events or publications that eventually culminate in a patent grant. Since multiple legal events are contained in the same file, we report the number of INPADOC threads, *not* the number of individual publications or issued patents within a given thread, unless otherwise noted. We have taken this approach because not all threads in INPADOC are complete, especially for developing countries, nor do they necessarily end with the granting of a patent. While our data may not provide the most up-to-date information on the legal status of a given filing, our data provide a sound global perspective on where patent rights are being pursued by various insulin manufacturers. We are further able to use this data to distinguish the type of technology described in different patent documents (e.g., insulin itself, method of manufacturing insulin, method of using insulin).

### Manufacturers

A list of putative insulin manufacturers [26] was generated based from two major sources: first, a literature review of global market research using LexisNexis® Academic, ProQuest®, various country market reports (e.g., Frost & Sullivan Market Report Reviews, Business Monitor International Pharmaceutical & Healthcare Industry Reports) [27, 28] and second, a review of the websites of various pharmaceutical companies and Medicine Regulatory Authorities (MRAs). We reviewed this generated list of putative insulin producing companies and searched WIPO PatentScope using the company name and the search term “insulin” found anywhere in either the front page of the WIPO published patent application or in the Abstract of the patent application, with a filing date more recent than 1 January 1994.

### Data storage and analysis

We created a single database for our main analysis, removed duplicates as well as any documents related to applications filed more than 20 years ago. In order to maintain focus upon insulin itself, we also set aside filings describing devices related to insulin administration. The complete dataset is in Additional file 3. Our findings in the area of insulin devices have been published elsewhere [29] and are briefly mentioned in the Discussion.

**Fig. 1:**
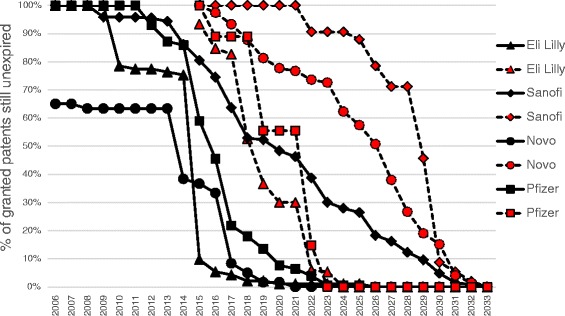
The percentage of all granted insulin patents remaining in force in a given year for Eli Lilly, Novo Nordisk, Sanofi Aventis and Pfizer

Beginning with patent filings as of 1995, we analyzed what percentage of all patent threads filed in that year remained in force over time. (See Fig. 1). We performed a simple correlation analysis using the non-parametric Spearman’s rank order correlation using Excel®. This statistical test is independent of whether or not the data is normally distributed. We looked at the association between number of patent threads per country and a) the diabetes disease burden of that country and b) the gross national income per capita of that country. See Additional file 4. Estimates of the average number of persons with diabetes (2007 and 2010) were obtained from the International Diabetes Federation Atlas [30]. The average gross national income per capita (current US dollars) was obtained for various countries from the World Bank for the years 1995–2015 [31].

## Results

### Global insulin patents

Most patents on insulin products in the world have already expired by 2015 yet many markets continue to be dominated by the brand-name versions marketed by original patent-holders. Figure 1 plots the percentage of all OB/HC granted patents on insulin remaining in force in any given year (based on a 20 year-from-filing patent life (black markers), and shows how relatively quickly the Eli Lilly, Novo and Pfizer insulin OB/HC patents are expiring compared to Sanofi. We confirm that after 2016, between about 5–20% of Pfizer, Eli Lilly and Novo Nordisk patents listed in the OB/HC remain un-expired and these percentages rapidly dimish, except for those of Sanofi who appears to have listed OB/HC patents whose expirations would extend well into 2030 and beyond (i.e., derived from a patent application filed in 2010).

Figure 1 also shows the percentage of all granted patents remaining in force on insulin in any given year (based on a 20 year-from-filing patent life) for the WIPO PatentScope data (red markers) for products not on OB/HC. Novo Nordisk has filed their non-OB/HC insulin patent appliations in a manner similar to Sanofi, such that Novo’s expirations tend to be spread out over many years, unlike the Lilly or Pfizer portfolios. This insulin patent portfolio of Eli Lilly is likely to expire at least a decade before that of Novo and Sanofi. The presence of Pfizer in the insulin landscape is mainly for the non-injectable powdered human insulin inhalation product Exubera® but it is certainly worth noting that in 2007, after 11 years of development and barely one full year of sales, Pfizer stopped its production [32].

Although Fig. 1 may look similar to a Kaplan-Meier survivorship analysis, it is not. Unlike a real-world survivorship analysis, there is no censoring of the data because all the “subjects” (i.e.,. patent threads) have the same lifespan, as it were. All threads expire at the end of 20 years from filing and all patent expiries are recorded. The step function is due to the fact that large groups of patent filings often come to the end of their patent term at roughly the same time.

**Fig. 2:**
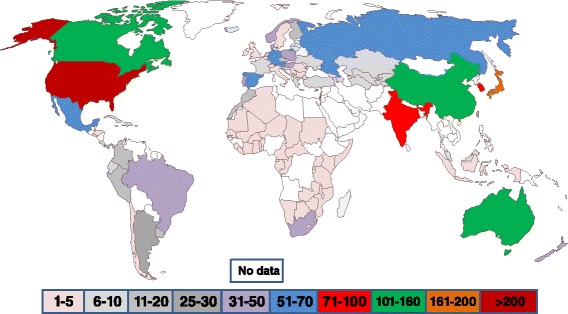
A map showing the number of insulin patent threads for Eli Lilly, Novo Nordisk, Sanofi Aventis based on patent applications filed after 1995. The different colors represent the number of insulin patent threads

The map in Fig. 2 shows the total number of patent threads found in the INPADOC database for Lilly, Sanofi, Novo and Pfizer for patent applications filed after 1995. In Africa, there are two regional patent offices, the Organisation Africaine de la Propriété Intellectuelle (OAPI) and the African Regional Intellectual Property Organization (ARIPO). Each is an intergovernmental organization for cooperation among African states in patent and other intellectual property matters. Both have the capacity to grant applications for patents in its member states who are parties to its patent protocol. OAPI and ARIPO refer to patent filings in countries of primarily French- West Africa and English- East Africa, respectively. 1 There are in total, 37 OAPI/ARIPO African countries but only between 1 and 5 patent thread filings per country were found (Fig. 2).

There is "no data" for some countries in Africa and many in the Middle East. This reflects either a lack of country data in INPADOC and/or a lack of interest in filing patent applications on the part of Lilly, Novo, Sanofi and/or Pfizer. Significantly, Africa has a low number of patent threads with South Africa having the highest. Indeed, the largest number of INPADOC patent thread filings are in the NAFTA countries (Mexico, U.S., Canada), the European Union countries (although not all), Japan and the BRIC countries (Brazil, Russia, India, China). Considering all the countries with evidence of INPADOC patent filings, the association between number of patent threads per country and the number of persons with diabetes in that country is moderate but significantly different than zero (Spearman rank correlation coefficient rho = 0.52; *p* < <0.005; df = 65). The association of patent thread per country with wealth per capita in that country was weaker and not significant (rho = 0.19; *p* = 0.12; df = 65).

The major players in the Canadian, US and European markets have, not surprisingly, filed patent applications outside these markets and have received issued patents on technology claimed by their rapidly expiring Orange Book/Health Canada patents. They will expire at about the same time as the corresponding US patent portfolios (Fig. 1, black markers). Where our study detected Orange Book/Health Canada national patent filings, 64.6% were in high income, 27.7% were upper-middle income, and the remaining 7.7% were in lower middle income countries. Most are restricted to North America, Europe, Australia, India and China.

### Global insulin manufacturers

Issued patents in low income settings were rare, even when we included regional patent regimes such as ARIPO or OAPI (Fig. 2). Particularly in Africa (Fig. 2), third parties may be free to exploit the technology claimed by the existing- and rapidly expiring-OB/HC patents as well as that for human insulin. Julphar (Gulf Pharmaceutical Industries, a UAE company), in early 2015 announced that construction of an insulin factory would start in Ethiopia [33].

Of the 40 putative insulin manufacturers identified in low- and middle-income countries, as of this writing we found only four (Biocon, Wockhardt, Tonghua Dongbao, Zhuhai Laboratories) that had any publicly available patent applications related to insulin. These four foreign manufacturers are filing patent applications primarily in Europe, the United States, Japan, China, India, South Korea, Israel, Russian Federation, Mexico, Malaysia, Canada, Australia, Ukraine, New Zealand and Egypt.

## Discussion

It has been suggested [5] that because 53 % of United States patents on insulin were linked to the delivery devices and not the insulin itself [9], intellectual property is not a barrier for earlier versions of insulin entering the market. Patentable innovations in insulin delivery devices are designed to extend the overall patent protection of medicine/device product combinations. Such innovations are incremental but very common [29].

The statement that insulin IP is not a barrier to market entry is accurate only for the presently marketed insulins not linked to devices (Fig. 1: black symbols), and the main insulin producers are continually filing for patents on analog insulins in their R&D pipelines so their market exclusivity (assuming that these patent applications mature into issued patents) are likely to continue for years to come (Fig. 1: red symbols). In short, analysis of publicly-available data on global insulin patents and manufacturers indicates that the vast majority of the world’s insulin markets are dominated by brand-name manufacturers long after the original product and process patents have expired.

The North American insulin market is dominated by the small number of companies who are the sole suppliers of one or more of six insulin analogs, which are available exclusively as brand name products. There is no US or Canadian human, non-analog insulin. Although third parties are likely free to exploit technology claimed by expiring OB/HC patents, it is possible that existing (i.e., non-expired) IP portfolios of Lilly, Novo, Sanofi and Pfizer in the U.S. and Canada (Fig. 1: red symbols) would prevent or hinder such exploitation. Given that the IP for recombinant human insulin, including DNA sequences and vectors is long off-patent, the existing insulin portfolios are unlikely to be sufficient to block production of human, recombinant insulin. Patent barriers are not the main reason for a lack of a generic version of recombinant human insulin in the U.S. marketplace or indeed, anywhere else in the world.

Moreover, insulin markets have evolved towards containing the newest, most expensive analog products not only in the US and Europe but in every measured insulin market in the world. These shifts greatly complicate access to medicines for the 2.8 billion people living on less than $2 a day, and for many living on higher incomes as well. Stimulating markets for acceptable, yet older products is critical for changing insulin market dynamics; otherwise, brand name companies will continue to introduce upgraded and patented products, deeming their older offerings as obsolete and pulling them from the market. We do not know what fraction of the domestic production of insulin in areas outside the US and Canada is based on producing insulin under license for Novo Nordisk, Lilly, Sanofi and possibly for Pfizer. The positive relationship between INPADOC patent threads for these four large multinational companies and diabetes prevalence (Additional file 3) we infer as manifestation of the scaling effect of market size.

We observed that only 10 % of the 40 putative insulin manufacturers identified in low- and middle-income countries were filing patent applications related to insulin. From this, we infer that they have intentions to market their own insulin in these countries and/or are already marketing their own insulin. For example, there are many companies making insulins for the Indian market and these products include, among others, purified bovine insulin (Bovine Longact® from USV), recombinant human insulin (Wosulin®: rDNA human monocomponent isophane Insulin from Wockhardt; Insugen®, human insulin from Biocon) and various insulin analogs (Lantus®- insulin glargine from Sanofi Aventis; Novomix-30®, Soluble insulin aspart 30 %, insulin aspart protamine 70 % from Novo Nordisk; Glaritus®, Insulin glargine from Wockhardt; Basalog®, insulin glargine from Biocon;) and combinations (e.g., Mixulin®, Porcine Insulin 30 %, Isophane Insulin 70 % from Cadila) [34–36].

Consider the following thought experiment: Assume Company X is producing both human analog insulin and human non-analog insulin in Ethiopia and wants to export both (i.e., respectively, a Lantus® and Humulin® equivalent) into the United States, Europe and a low income country (LIC). At the outset, we reiterate that within a few years patents in all these destinations (U.S., Europe and the LIC), if they exist at all, are unlikely to be a barrier to commercialization of the analog and there are no IP barriers to production of recombinant human insulin.

What regulatory options exist to stimulate more competitive insulin markets? First, if imported into the US or made in the US under contract with Company X, both insulins will be regulated as a “drug” not as a biologic [37] and the regulatory dossier would be under the ANDA (“Abbreviated New Drug Application” pathway of US FDA Section 505(b)2. Indeed, this pathway was already used in 2006 for approval of a generic recombinant growth hormone product, Omnitrope® by Sandoz relying in-part on the FDA’s prior approval of Pfizer’s pioneer rhGH product, Genotropin® [38].

In August 2014, the US FDA granted tentative approval for Eli Lilly’s Basaglar®, a recombinantly produced insulin glargine analog for treating diabetes. As a 505(b)(2) product, approval relied in part on clinical studies carried out for the originator, Sanofi's Lantus® (insulin glargine). Basaglar® does not have final approval due to patent litigation involving Sanofi's patents. Time to tentative approval was rapid, however. It was exactly ten months [39]. The same product was approved as a “biosimilar” in 2014 in Europe. In the US and Europe, a recombinant version of non-analog human insulin would follow the same respective pathways [40]. Analog insulin glargine has recently been approved in Mexico [41] according to the biocomparable approvals pathway defined in 2012 (i.e., Galactus®, under license to PiSA Pharmaceuticals). A key issue, at least for the United States FDA, is whether a biosimilar insulin can be freely substituted at the pharmacy level [42]. The interchangeability of different small-molecule generics leads to substantially reduced drug pricing. When there is no interchangeability, it is not clear whether or not price competition will have an impact unless there is coherence with other policy interventions [43].

It is an open question as to whether or not the LICs could rely on the regulatory authorities in the US, India or Mexico and allow marketing of a version of glargine or human insulin. Notwithstanding the relative ease of US and European approval of Basaglar®, different manufacturing processes may result in subtly different insulin products. Such differences between versions of all insulins and their respective reference products could be expected [43].

Regulatory solutions can only partly address the structural problems contributing to uncompetitive off-patent insulin markets, if they do not address the broader problems of physician and patient preference. One of the biggest barriers to widespread access is the fact that doctors may be influenced by claims that insulin analogs are superior to human insulin when the evidence is equivocal. According to the WHO, no clear advantage (with lack of clinically important benefits) of analog insulin over recombinant human insulin has been established [44]. To be sure, if there are clinical complications associated with human insulin use, patients may indeed not want to switch from analog products to a human generic. In markets dominated by analogs, when a patient gets diagnosed (and needs insulin), he/she will likely be given the (multinational) analog insulin. If the patient feels better, they would want to continue with the same (analog) insulin and not switch to other (human) products/brands. Switching to another insulin would mean that a patient will have to regularly visit the doctor for tests/readings, and the patient would likely prefer to remain stable with one insulin. Simply put, the multinational companies have a wide physician network which reinforces their brand perceptions. In low- and middle-income countries where human insulin is still the predominant market share [45] this behavioral situation may well be less onerous yet, irrespective of insulin type, we suspect physician acceptance is a critical access barrier to overcome.

Finally, once approved for market, the buyers of, as well as the payers for, these generic human insulins will need to negotiate for price, although in the US this opportunity is limited [46]. At present, the major sellers of insulin are well organized and their buyers are not. As pointed out recently [5], by contrast with antiretrovirals, which were paid for by donors such as the Global Fund, insulin is not purchased by donors, but rather directly from country budgets. In situations where pooled procurement of essential medicines is ongoing [47, 48] or proposed [49], its implementation may have a great influence on procurement prices for insulins of all types. Pooled procurement, in principle, avoids the costs of sustaining local production facilities that may not be viable in any case. However, it is difficult to investigate the extent to which such pooled procurement is effective in significantly increasing medicine penetration at the national level. But if the end result is that lower prices are being offered and more patients have access to medicines, the health system still benefits. One lesson from the ARV situation is that a possible barrier to pooled procurement is a lack of regulatory and procurement capacity at the country level [50].

Another option that has been used is a restricted tender system (in contrast to open tenders) for purchasing from well-known pre-qualified suppliers whose products have been previously authorised and with whom the procurement authority has had satisfactory results. However, a potential concern is that restricted tendering rounds may increase the likelihood of market concentration if the same suppliers win contracts, so that competitors let their product market authorisations expire. This is challenge for buyers to be mindful of. Some level of competition is naturally critical for tendering to work effectively, bearing in mind that quality and the continuity of supply are also important considerations [51].

Some arrangements allowing for tenders might be set up in a way that several manufacturers are selected for supplying the medicine at the same price. If this can be done so that competition is still suppressing prices, this might, in principle, prevent excessive concentration and its negative effects on future prices [52]. Further, the time period for which tenders are awarded could be limited to encourage more diversity in the market. Other criteria besides price can be included in a request for tender, such as quality of the product, quality of the delivery system (e.g., insulin vials versus insulin pens) and security of supply. The tendering system could be structured to ensure patients and their doctors retain adequate choice of subsidised treatments.

A limitation of our method is that, in order for our study to be feasible and replicable, we confined our international patent search to the only international patent databases freely available (i.e., the EPO’s INPADOC via Espacenet, WIPO PatentScope) and India’s national patent database where many major generic pharmaceutical companies are based. However, there are other premium international patent databases (e.g., Derwent) and all other national patent databases [20, 53] which may yield additional records. Nonetheless, the EPO and the WIPO facilitate procedures and communications on a global or regional level. These organizations have the most official and complete information on global applications as well as adjunct information. They should always be used for any serious research that has legal and financial ramifications and for verifying information found in other sources.

## Conclusions

This global analysis of patents and producers of global insulin documents that for most of the world there is little to no alternatives to brand-named analog insulins and non-analog human alternatives in low- and middle-income countries. The market dominance of analog over human insulin is not a function of intellectual property exclusivity as patents on human insulin have expired long ago. Although patents on the current portfolio of analogs will expire very soon, there are many patent filings and granted patents on insulins that are not marketed in the United States so a very few companies are enjoying complete monopolies in these markets for a surprisingly long time. The moderate, but statistically significant association between patent filings and diabetes disease burden suggests these multinationals are filing for more patents in the bigger markets with large numbers of persons with diabetes and an rapidly emerging middle class, although these bigger markets (Brazil, India, China) are not the wealthiest per capita. This should not be a surprise to anyone.

Mapping the patent estates on insulins is a first step in encouraging manufactures globally to consider this opportunity to enter other, far smaller markets. We need to identify other bottlenecks in the global insulin market and to ensure that by 2022, the centenary of the first person with diabetes to be treated with insulin, all those requiring this life-saving medicine have access to affordable versions of it. More than 80 % of diabetes deaths occur in low- and middle-income countries [54].

Yet there are also many challenges in trying to produce copies using current biotechnological approaches without an appropriate internationally agreed process of regulation. Off-patent human insulins can effectively manage diabetes. Others have observed the need for older insulins to be manufactured [10] and our findings support and underscore this need. A practical way forward would be to find (potential) generic manufacturers globally and incentivize them towards opportunities to diversify their national insulin markets with acceptable off-patent products for export.

## Additional file

Additional file 1: Product Table: Patented products in North American market stratified by company and insulin analogue. (DOCX 15.2 KB) Additional file 2: Analysis of patent searching using ‘insulin’ and/or ‘analogue/analog”. A. doc file with search results showing that using the word “analog” or “analogue” as an added search term does not increase the number of patent search result “hits”. (DOCX 131 KB) Additional file 3: Complete dataset of issued patents and patent applications. This file has two tabs: “Post-1995” contains results of all patent searches in which the initial patent application has a priority date after 31 December 1994. “Family size comparison” analyses the number of different patent “threads” for the data in the prior tab. Patent ‘threads” are described in the text. (XLSX 876 KB) Additional file 4: Spearman’s Rho coefficient. This file shows the data used to calculate the non-parametric Spearman’s rank correlation between the number of patent ‘threads” and the a) average number of persons with diabetes x 100 (2007–2010); b) the average GNI/capital (1995–2015). (XLSX 18.6 KB)

## Footnotes

1 The OAPI countries include: : Benin, Burkina Faso, Cameroon, Central African Republic, Chad, Comoros, Congo, Côte d'Ivoire, Gabon, Guinea, Equatorial Guinea, Mali, Mauritania, Niger, Guinea Bissau, Senegal and Togo. The ARIPO countries include: Botswana, Gambia, Ghana, Kenya, Lesotho, Liberia, Malawi, Mozambique, Namibia, Rwanda, Sào Tomé and Príncipe, Sierra Leone, Somalia, Sudan, Swaziland,Tanzania,Uganda,Zambia, Zimbabwe
