# Supplementary material for: The global intellectual property ecosystem for insulin and its public health implications: an observational study
Source: J Pharm Policy Pract. 2016 Jul 19;10:3. doi: 10.1186/s40545-016-0072-8 (PMC4955122; doi:10.1186/s40545-016-0072-8)
Supplement: Additional file 1: — Product Table: Patented products in North American market stratified by company and insulin analogue. (DOCX 15.2 KB) [file 40545_2016_72_MOESM1_ESM.docx]

| **Eli Lilly** |  |  |
| --- | --- | --- |
| **INN and proprietary name** | **Route** | **Strength** |
| INSULIN LISPRO PROTAMINE RECOMBINANT; INSULIN LISPRO RECOMBINANT |  |  |
| HUMALOG MIX 50/50 | INJECTABLE;INJECTION | 50 UNITS/ML;50 UNITS/ML |
| HUMALOG MIX 50/50 KWIKPEN | INJECTABLE;INJECTION | 50 UNITS/ML;50 UNITS/ML |
| HUMALOG MIX 75/25 | INJECTABLE;INJECTION | 75 UNITS/ML;25 UNITS/ML |
| HUMALOG MIX 75/25 KWIKPEN | INJECTABLE;INJECTION | 75 UNITS/ML;25 UNITS/ML |
|  |  |  |
|  |  |  |
|  |  |  |
| INSULIN LISPRO RECOMBINANT |  |  |
| HUMAJECT HUMALOG | solution for injection | 100 U/ml |
|  |  |  |
|  |  |  |
| HUMALOG | INJECTABLE;INJECTION | 100 U/ml |
| HUMALOG KWIKPEN | INJECTABLE;INJECTION | 100 UNITS/ML |
| INSULIN RECOMBINANT HUMAN |  |  |
| HUMULIN R | INJECTABLE;INJECTION | 500 UNITS/ML |

Additional File 1. Patented products in North American market stratified by company and insulin analogue

| **NOVO NORDISK INC** |  |  |
| --- | --- | --- |
| **INN and proprietary name** | **Route** | **Strength** |
| INSULIN ASPART PROTAMINE RECOMBINANT; INSULIN ASPART RECOMBINANT |  |  |
| NOVOLOG MIX 70/30 | INJECTABLE;SUBCUTANEOUS | 700 UNITS/10ML; 300 UNITS/10ML (70 UNITS/ML; 30 UNITS/ML) |
| NOVOLOG MIX 70/30 FLEXPEN | INJECTABLE;SUBCUTANEOUS | 210 UNITS/3ML; 90 UNITS/3ML (70 UNITS/ML; 30 UNITS/ML) |
| INSULIN ASPART RECOMBINANT |  |  |
| NOVOLOG | INJECTABLE;SUBCUTANEOUS | 1000 UNITS/10ML (100 UNITS/ML) |
| NOVOLOG FLEXPEN | INJECTABLE;SUBCUTANEOUS | 300 UNITS/3ML (100 UNITS/ML) |
| NOVOLOG FLEXTOUCH | INJECTABLE;SUBCUTANEOUS | 300 UNITS/3ML (100 UNITS/ML) |
| NOVOLOG PENFILL | INJECTABLE;SUBCUTANEOUS | 300 UNITS/3ML (100 UNITS/ML) |
|  |  |  |
|  |  |  |
|  |  |  |
| NOVORAPID | INJECTABLE;SUBCUTANEOUS | 100 U/ml |
| INSULIN DETEMIR RECOMBINANT |  |  |
| LEVEMIR | INJECTABLE;SUBCUTANEOUS | 1000 UNITS/10ML (100 UNITS/ML) |
| LEVEMIR FLEXPEN | INJECTABLE;SUBCUTANEOUS | 300 UNITS/3ML (100 UNITS/ML) |
| LEVEMIR FLEXTOUCH | INJECTABLE;SUBCUTANEOUS | 300 UNITS/3ML (100 UNITS/ML) |

| Pfizer | |  |  |
| --- | --- | --- | --- |
| **INN and proprietary name** | | **Route** | **Strength** |
| INSULIN RECOMBINANT HUMAN (inhaled) | | | |
| EXUBERA | powder | | 1mg |
| EXUBERA | powder | | 3mg |

| **SANOFI AVENTIS** |  |  |
| --- | --- | --- |
| **INN and proprietary name** | **Route** | **Strength** |
| INSULIN GLARGINE RECOMBINANT |  |  |
| LANTUS | INJECTABLE;INJECTION (10 ml vial solution) | 100 UNITS/ML |
| LANTUS SOLOSTAR | INJECTABLE;INJECTION | 300 UNITS/3ML (100 UNITS/ML) |
| APIDRA | INJECTABLE;IV (INFUSION), SUBCUTANEOUS | 1000 UNITS/10ML (100 UNITS/ML) |
| APIDRA | INJECTABLE;IV (INFUSION), SUBCUTANEOUS | 300 UNITS/3ML (100 UNITS/ML) |
| APIDRA (10 ML VIAL) | solution | 100 U/mL |
| APIDRA (3ML CARTRIDGE) | solution | 100 U/mL |
| APIDRA (3ML OPTISET) | solution | 100 U/mL |
| APIDRA (3ML SOLOSTAR) | solution | 100 U/mL |
| APIDRA SOLOSTAR | INJECTABLE;SUBCUTANEOUS | 300 UNITS/3ML |
| INSULIN RECOMBINANT HUMAN (inhaled) | | |
| AFREZZA | POWDER;INHALATION | 4 UNITS/INH |
| AFREZZA | POWDER;INHALATION | 8 UNITS/INH |
