# Supplementary material for: The global intellectual property ecosystem for insulin and its public health implications: an observational study
Source: J Pharm Policy Pract. 2016 Jul 19;10:3. doi: 10.1186/s40545-016-0072-8 (PMC4955122; doi:10.1186/s40545-016-0072-8)
Supplement: Additional file 2: — Analysis of patent searching using ‘insulin’ and/or ‘analogue/analog”. A. doc file with search results showing that using the word “analog” or “analogue” as an added search term does not increase the number of patent search result “hits”. (DOCX 131 KB) [file 40545_2016_72_MOESM2_ESM.docx]

• The term “insulin” provided a better retrieval of relevant patents than “analogue” or any combination of these two terms. We tested this using two different search engines, WIPO Patentscope and the US Patent Office.

*WIPO Patentscope*: Searching the “front page” of all patent documents of Eli Lilly for the word “insulin”  between January 1994 to 1 January 2015. This search includes the title and abstract.

**Search 1:** insulin* : 371

**Search 2:** analog* : 494

**Search 3:** analog* OR insulin*: 371

**Search 4:** analog* AND insulin*: 162

For this database**,**  “insulin” alone is the superior search term.  Adding the term “analog” to the search terms only added irrelevant results to our search. When we searched “insulin”,  we received 371 “hits”. When using “insulin OR analog”, we got 371. Search 4 shows that of the 371 results, 162 also mention “analog”. The first 200 results from Search 2 above are listed starting on Page 2 of this file.  Of these 200, 185 (about 93%) were documents directed to irrelevant proteins.

*US Patent Office*: Searching the abstract alone (similar- but not idential to the “Front Page” of the WIPO database engine) of all patent documents of Novo Nordisk and Eli Lilly for the word “insulin”, “analogue” or both  between January 1994 to 1 January 2015.

**Novo Search 1 :** insulin : 125

**Novo Search 2:** analog : 24

**NovoSearch 3:** analog OR insulin: 136

**Novo Search 4:** analog AND insulin: 0

• In the “analogue” search, only 13 patents were duplicative of the “insulin” search. So the remaining 11 were  presumed “new” (about 9%:  11/125). Of the 11 “new” patents in the ‘analogue’ search,  none were related in any way to insulin. Conversely, of the 11 “extra” patents in search 3, none were related to insulin.

**Lilly Search 1 :** insulin : 59

**Lilly Search 2:** analog : 1

**Lilly Search 3:** analog OR insulin: 60

**Lilly Search 4:** analog AND insulin: 0

• As in the prior search, using “analogue” provides no extra benefit. The single “new” patent was not directed to insulin."

| **Title** | | | **Ctr** | **PubDate** |
| --- | --- | --- | --- | --- |
| **Int.Class** | **Appl.No** | **Applicant** | **Inventor** | |
| 1. [2809651](https://patentscope.wipo.int/search/en/detail.jsf?docId=EP128395525&recNum=1&office=&queryString=FP%3Aanalog*+AND+PA%3ALilly+AND+PD%3A%28%5B01.01.1994+to+01.01.2015%5D%29+&prevFilter=&sortOption=Pub+Date+Desc&maxRec=494) **BENZYL SULFONAMIDE DERIVATIVES USEFUL AS MOGAT - 2 INHIBITORS** | | | EP | 10.12.2014 |
| \| C07D 213/64 \| Top of Form  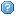  Bottom of Form \| \| --- \| --- \| | 13703234 | LILLY CO ELI | FERNANDEZ MARIA CARMEN | |
| The present invention provides compounds of Formula (I) below: and analogues thereof where the various substituent groups, R1, R2, R3, R4, R5 A, and X are described herein; or a pharmaceutical salt thereof; a method of treating a condition such as hypertriglyceridemia and a process for preparing the compounds. | | | | |
| 2. [2515928](https://patentscope.wipo.int/search/en/detail.jsf?docId=PT123793693&recNum=2&office=&queryString=FP%3Aanalog*+AND+PA%3ALilly+AND+PD%3A%28%5B01.01.1994+to+01.01.2015%5D%29+&prevFilter=&sortOption=Pub+Date+Desc&maxRec=494) **OXYNTOMODULIN PEPTIDE ANALOGUE** | | | PT | 03.09.2014 |
| \| A61K 38/17 \| Top of Form  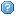  Bottom of Form \| \| --- \| --- \| | 10801033 | LILLY CO ELI | ALSINA-FERNANDEZ JORGE | |
| ABSTRACT The present invention provides an Oxyntomodulin peptide analogue useful in the treatment of diabetes and/or obesity. | | | | |
| 3. [11201404106Q](https://patentscope.wipo.int/search/en/detail.jsf?docId=SG131298201&recNum=3&office=&queryString=FP%3Aanalog*+AND+PA%3ALilly+AND+PD%3A%28%5B01.01.1994+to+01.01.2015%5D%29+&prevFilter=&sortOption=Pub+Date+Desc&maxRec=494) **BENZYL SULFONAMIDE DERIVATIVES USEFUL AS MOGAT - 2 INHIBITORS** | | | SG | 28.08.2014 |
| \| C07D 213/64 \| Top of Form  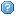  Bottom of Form \| \| --- \| --- \| | 11201404106Q | ELI LILLY AND COMPANY | FERNANDEZ, Maria Carmen | |
| (12) INTERNATIONAL APPLICATION PUBLISHED UNDER THE PATENT COOPERATION TREATY (PCT) (19) World Intellectual Property Organization International Bureau (43) International Publication Date 8 August 2013 (08.08.2013) WIPOIPCT (10) International Publication Number WO 2013/116075 A1 (51) International Patent Classification: C07D 213/64 (2006.01) C07D 213/65 (2006.01) A61K31/4402 (2006.01) A61P 3/10 (2006.01) C07C 311/05 (2006.01) (21) International Application Number: (22) International Filing Date: (25) Filing Language: (26) Publication Language: PCT/US2013/022870 24 January 2013 (24.01.2013) English (30) Priority Data: 61/592,717 31 January 2012 (31.01.2012) 12382432.8 6 November 2012 (06.11.2012) English US EP (71) Applicant: ELI LILLY AND COMPANY [US/US]; Lilly Corporate Center, Indianapolis, Indiana 46285 (US). (72) Inventors: FERNANDEZ, Maria Carmen; c/o Eli Lilly and Company, P.O. Box 6288, Indianapolis, Indiana 46206-6288 (US). GONZALEZ-GARCIA, Maria Rosar- io; c/o Eli Lilly and Company, P.O. Box 6288, Indianapol­ is, Indiana 46206-6288 (US). PFEIFER, Lance Allen; c/o Eli Lilly and Company, P.O. Box 6288, Indianapolis, Indi­ ana 46206-6288 (US). (74) Agents: MYERS, James et al.; Eli Lilly And Company, P.O. Box 6288, Indianapolis, Indiana 46206-6288 (US). (81) Designated States (unless otherwise indicated, for every kind of national protection available)'. AE, AG, AL, AM, AO, AT, AU, AZ, BA, BB, BG, BH, BN, BR, BW, BY, BZ, CA, CH, CL, CN, CO, CR, CU, CZ, DE, DK, DM, DO, DZ, EC, EE, EG, ES, FI, GB, GD, GE, GH, GM, GT, HN, HR, HU, ID, IL, IN, IS, JP, KE, KG, KM, KN, KP, KR, KZ, LA, LC, LK, LR, LS, LT, LU, LY, MA, MD, ME, MG, MK, MN, MW, MX, MY, MZ, NA, NG, NI, NO, NZ, OM, PA, PE, PG, PH, PL, PT, QA, RO, RS, RU, RW, SC, SD, SE, SG, SK, SL, SM, ST, SV, SY, TH, TJ, TM, TN, TR, TT, TZ, UA, UG, US, UZ, VC, VN, ZA, ZM, ZW. (84) Designated States (unless otherwise indicated, for every kind of regional protection available)'. ARIPO (BW, GH, GM, KE, LR, LS, MW, MZ, NA, RW, SD, SL, SZ, TZ, UG, ZM, ZW), Eurasian (AM, AZ, BY, KG, KZ, RU, TJ, TM), European (AL, AT, BE, BG, CH, CY, CZ, DE, DK, EE, ES, FI, FR, GB, GR, HR, HU, IE, IS, IT, LT, LU, LV, MC, MK, MT, NL, NO, PL, PT, RO, RS, SE, SI, SK, SM, TR), OAPI (BF, BJ, CF, CG, CI, CM, GA, GN, GQ, GW, ML, MR, NE, SN, TD, TG). Declarations under Rule 4.17: — as to applicant's entitlement to apply for and be granted a patent (Rule 4.17(H)) — as to the applicant's entitlement to claim the priority of the earlier application (Rule 4.17(iii)) Published: — with international search report (Art. 21(3)) (54) Title: BENZYL SULFONAMIDE DERIVATIVES USEFUL AS MOGAT - 2 INHIBITORS i> o CJ (i) (57) Abstract: The present invention provides compounds of Formula (I) below: and analogues thereof where the various substitu - © ent groups, Rl, R2, R3, R4, R5 A, and X are described herein; or a pharmaceutical salt thereof; a method of treating a condition such as hypertriglyceridemia and process a for preparing the compounds. | | | | |
| 4. [088351](https://patentscope.wipo.int/search/en/detail.jsf?docId=AR128933302&recNum=4&office=&queryString=FP%3Aanalog*+AND+PA%3ALilly+AND+PD%3A%28%5B01.01.1994+to+01.01.2015%5D%29+&prevFilter=&sortOption=Pub+Date+Desc&maxRec=494) **ANALOGOS DE PIRAZOL SUSTITUIDOS** | | | AR | 28.05.2014 |
| \|  \|  \| \| --- \| --- \| | P120103851 | ELI LILLY AND COMPANY |  | |
| Métodos para tratar osteoartritis y el dolor asociado con osteoartritis usando los compuestos; y proceso para preparar los compuestos. Reivindicación 1: Un compuesto que tiene una fórmula (1) caracterizado porque: A es CH o N; X es CH o N; R¹ se selecciona de: -SO₂CH₃, -SO₂N(CH₃)₂, -C(O)N(R³)₂, -C(O)R⁴, y -NHSO₂CH₃; R² se selecciona de: -alquilo C₃₋₄, -OCH(CH₃)₂, y -SCH(CH₃)₂; cada R³ se selecciona independientemente de: H y -CH₃; R⁴ se selecciona de: 4-morfolinilo, 1-piperidinilo, 4-tiomorfolinilo, -NH(CH₂)₃OH, y 4-metil-1-piperazinilo; y siempre que cuando uno de A o X es N, el otro de A o X es CH; o una sal del mismo farmacéuticamente aceptable. | | | | |
| 5. [WO/2014/031420](https://patentscope.wipo.int/search/en/detail.jsf?docId=WO2014031420&recNum=5&office=&queryString=FP%3Aanalog*+AND+PA%3ALilly+AND+PD%3A%28%5B01.01.1994+to+01.01.2015%5D%29+&prevFilter=&sortOption=Pub+Date+Desc&maxRec=494) **HOMODIMERIC PROTEINS** | | | WO | 27.02.2014 |
| \| [C07K 14/605](http://www.wipo.int/ipcpub/?symbol=C07K0014605000&refresh=page&viewmode=a&notes=no&headings=no&showdeleted=no) \| Top of Form  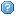  Bottom of Form \| \| --- \| --- \| | PCT/US2013/055041 | ELI LILLY AND COMPANY | ALSINA-FERNANDEZ, Jorge | |
| This present invention relates to a homodimeric protein comprising fibroblast growth factor 21 (FGF21) and glucagon-like peptide (GLP-1), pharmaceutical compositions comprising the homodimeric protein, and methods for treating type 2 diabetes, obesity, dyslipidemia, and/or metabolic syndrome using such homodimeric protein. | | | | |
| 6. [WO/2013/116075](https://patentscope.wipo.int/search/en/detail.jsf?docId=WO2013116075&recNum=6&office=&queryString=FP%3Aanalog*+AND+PA%3ALilly+AND+PD%3A%28%5B01.01.1994+to+01.01.2015%5D%29+&prevFilter=&sortOption=Pub+Date+Desc&maxRec=494) **BENZYL SULFONAMIDE DERIVATIVES USEFUL AS MOGAT - 2 INHIBITORS** | | | WO | 08.08.2013 |
| \| [C07D 213/64](http://www.wipo.int/ipcpub/?symbol=C07D0213640000&refresh=page&viewmode=a&notes=no&headings=no&showdeleted=no) \| Top of Form  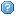  Bottom of Form \| \| --- \| --- \| | PCT/US2013/022870 | ELI LILLY AND COMPANY | FERNANDEZ, Maria Carmen | |
| The present invention provides compounds of Formula (I) below: and analogues thereof where the various substituent groups, R1, R2, R3, R4, R5 A, and X are described herein; or a pharmaceutical salt thereof; a method of treating a condition such as hypertriglyceridemia and a process for preparing the compounds. | | | | |
| 7. [2859995](https://patentscope.wipo.int/search/en/detail.jsf?docId=CA108110293&recNum=7&office=&queryString=FP%3Aanalog*+AND+PA%3ALilly+AND+PD%3A%28%5B01.01.1994+to+01.01.2015%5D%29+&prevFilter=&sortOption=Pub+Date+Desc&maxRec=494) **BENZYL SULFONAMIDE DERIVATIVES USEFUL AS MOGAT - 2 INHIBITORS** | | | CA | 08.08.2013 |
| \| C07D 213/64 \| Top of Form  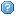  Bottom of Form \| \| --- \| --- \| | 2859995 | ELI LILLY AND COMPANY |  | |
| The present invention provides compounds of Formula (I) below: and analogues thereof where the various substituent groups, R1, R2, R3, R4, R5 A, and X are described herein; or a pharmaceutical salt thereof; a method of treating a condition such as hypertriglyceridemia and a process for preparing the compounds. | | | | |
| 8. [20130197039](https://patentscope.wipo.int/search/en/detail.jsf?docId=US90328499&recNum=8&office=&queryString=FP%3Aanalog*+AND+PA%3ALilly+AND+PD%3A%28%5B01.01.1994+to+01.01.2015%5D%29+&prevFilter=&sortOption=Pub+Date+Desc&maxRec=494) **Benzyl sulfonamide derivatives useful as MOGAT-2 inhibitors** | | | US | 01.08.2013 |
| \| C07D 211/72 \| Top of Form  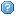  Bottom of Form \| \| --- \| --- \| | 13748627 | Eli Lilly and Company | Fernandez Maria Carmen | |
| The present invention provides compounds of Formula below:  and analogues thereof where the various substituent groups, R1, R2, R3, R4, R5 A, and X are described herein; or a pharmaceutical salt thereof; a method of treating a condition such as hypertriglyceridemia and a process for preparing the compounds. | | | | |
| 9. [WO/2013/066640](https://patentscope.wipo.int/search/en/detail.jsf?docId=WO2013066640&recNum=9&office=&queryString=FP%3Aanalog*+AND+PA%3ALilly+AND+PD%3A%28%5B01.01.1994+to+01.01.2015%5D%29+&prevFilter=&sortOption=Pub+Date+Desc&maxRec=494) **SUBSTITUTED PYRAZOLE ANALOGUES AS RAR ANTAGONISTS** | | | WO | 10.05.2013 |
| \| [C07D 231/12](http://www.wipo.int/ipcpub/?symbol=C07D0231120000&refresh=page&viewmode=a&notes=no&headings=no&showdeleted=no) \| Top of Form  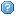  Bottom of Form \| \| --- \| --- \| | PCT/US2012/060995 | ELI LILLY AND COMPANY | BLEISCH, Thomas, John | |
| The present invention provides compounds of Formula I or a pharmaceutical salt thereof; methods of treating osteoarthritis and the pain associated with osteoarthritis using the compounds; and processes for preparing the compounds. | | | | |
| 10. [2850516](https://patentscope.wipo.int/search/en/detail.jsf?docId=CA105501519&recNum=10&office=&queryString=FP%3Aanalog*+AND+PA%3ALilly+AND+PD%3A%28%5B01.01.1994+to+01.01.2015%5D%29+&prevFilter=&sortOption=Pub+Date+Desc&maxRec=494) **SUBSTITUTED PYRAZOLE ANALOGUES AS RAR ANTAGONISTS** | | | CA | 10.05.2013 |
| \| C07D 231/12 \| Top of Form  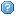  Bottom of Form \| \| --- \| --- \| | 2850516 | ELI LILLY AND COMPANY |  | |
| The present invention provides compounds of Formula I or a pharmaceutical salt thereof; methods of treating osteoarthritis and the pain associated with osteoarthritis using the compounds; and processes for preparing the compounds. | | | | |
| 11. [34077](https://patentscope.wipo.int/search/en/detail.jsf?docId=MA128924358&recNum=11&office=&queryString=FP%3Aanalog*+AND+PA%3ALilly+AND+PD%3A%28%5B01.01.1994+to+01.01.2015%5D%29+&prevFilter=&sortOption=Pub+Date+Desc&maxRec=494) | | | MA | 05.03.2013 |
| \| A61K 38/16 \| Top of Form  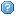  Bottom of Form \| \| --- \| --- \| | 35233 | ELI LILLY AND COMPANY | ALSINA-FERNANDEZ, Jorge | |
| La présente invention porte sur le domaine du traitement du diabète et concerne des peptides qui présentent une activité pour, à la fois, le récepteur du peptide insulinotrope dépendant du glucose (GIP-R) et le récepteur du peptide-1 de type glucagon (GLP-1-R) et qui leur sont sélectifs par rapport au récepteur du glucagon (Gluc-R). Plus précisément, l'invention concerne des analogues de GIP avec des substitutions d'acide aminé introduites pour moduler l'activité d'à la fois GIP-R et de GLP-1-R et pour maintenir une sélectivité par rapport à Gluc-R. | | | | |
| 12. [2552471](https://patentscope.wipo.int/search/en/detail.jsf?docId=EP76087732&recNum=12&office=&queryString=FP%3Aanalog*+AND+PA%3ALilly+AND+PD%3A%28%5B01.01.1994+to+01.01.2015%5D%29+&prevFilter=&sortOption=Pub+Date+Desc&maxRec=494) **NOVEL PEPTIDES AND METHODS FOR THEIR PREPARATION AND USE** | | | EP | 06.02.2013 |
| \| A61K 38/16 \| Top of Form  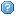  Bottom of Form \| \| --- \| --- \| | 11711424 | LILLY CO ELI | ALSINA-FERNANDEZ JORGE | |
| The present invention is in the field of treatment of diabetes and relates to peptides that exhibit activity for both glucose-dependent insulinotropic peptide receptor (GIP-R) and glucagon-like peptide- 1 receptor (GLP-1-R) and are selective over glucagon receptor (Gluc-R). Specifically provided are GIP analogs with amino acid substitutions introduced to modulate activity for both GIP-R and GLP-1-R and maintain selectivity over Gluc-R. | | | | |
| 13. [1951658](https://patentscope.wipo.int/search/en/detail.jsf?docId=PT108253165&recNum=13&office=&queryString=FP%3Aanalog*+AND+PA%3ALilly+AND+PD%3A%28%5B01.01.1994+to+01.01.2015%5D%29+&prevFilter=&sortOption=Pub+Date+Desc&maxRec=494) **GLUCAGON RECEPTOR ANTAGONISTS, PREPARATION AND THERAPEUTIC USES** | | | PT | 12.11.2012 |
| \| C07C 233/83 \| Top of Form  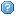  Bottom of Form \| \| --- \| --- \| | 06850148 | LILLY CO ELI | ZHU GUOXIN | |
| The present invention discloses novel compounds of Formula I, or pharmaceutically acceptable salts thereof, which have glucagon receptor antagonist or inverse agonist activity, as well as methods for preparing such compounds. In another embodiment, the invention discloses pharmaceutical compositions comprising compounds of Formula I as well as methods of using them to treat diabetic and other glucagon related metabolic disorders, and the like. | | | | |
| 14. [2515928](https://patentscope.wipo.int/search/en/detail.jsf?docId=EP74863054&recNum=14&office=&queryString=FP%3Aanalog*+AND+PA%3ALilly+AND+PD%3A%28%5B01.01.1994+to+01.01.2015%5D%29+&prevFilter=&sortOption=Pub+Date+Desc&maxRec=494) **OXYNTOMODULIN PEPTIDE ANALOGUE** | | | EP | 31.10.2012 |
| \| A61K 38/17 \| Top of Form  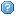  Bottom of Form \| \| --- \| --- \| | 10801033 | LILLY CO ELI | ALSINA-FERNANDEZ JORGE | |
| ABSTRACT The present invention provides an Oxyntomodulin peptide analogue useful in the treatment of diabetes and/or obesity. | | | | |
| 15. [2515927](https://patentscope.wipo.int/search/en/detail.jsf?docId=EP74863053&recNum=15&office=&queryString=FP%3Aanalog*+AND+PA%3ALilly+AND+PD%3A%28%5B01.01.1994+to+01.01.2015%5D%29+&prevFilter=&sortOption=Pub+Date+Desc&maxRec=494) **OXYNTOMODULIN PEPTIDE ANALOGUE** | | | EP | 31.10.2012 |
| \| A61K 38/17 \| Top of Form  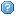  Bottom of Form \| \| --- \| --- \| | 10799178 | LILLY CO ELI | ALSINA-FERNANDEZ JORGE | |
| The present invention provides Oxyntomodulin peptide analogues useful in the treatment of diabetes and/or obesity. | | | | |
| 16. [220093](https://patentscope.wipo.int/search/en/detail.jsf?docId=il75634374&recNum=16&office=&queryString=FP%3Aanalog*+AND+PA%3ALilly+AND+PD%3A%28%5B01.01.1994+to+01.01.2015%5D%29+&prevFilter=&sortOption=Pub+Date+Desc&maxRec=494) **OXYNTOMODULIN PEPTIDE ANALOGUE** | | | il | 24.09.2012 |
| \| A61K / \| Top of Form  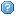  Bottom of Form \| \| --- \| --- \| | 220093 | ELI LILLY AND COMPANY |  | |
|  | | | | |
| 17. [2496249](https://patentscope.wipo.int/search/en/detail.jsf?docId=EP74562661&recNum=17&office=&queryString=FP%3Aanalog*+AND+PA%3ALilly+AND+PD%3A%28%5B01.01.1994+to+01.01.2015%5D%29+&prevFilter=&sortOption=Pub+Date+Desc&maxRec=494) **GLP-1 RECEPTOR AGONIST COMPOUNDS FOR OBSTRUCTIVE SLEEP APNEA** | | | EP | 12.09.2012 |
| \| A61K 38/26 \| Top of Form  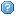  Bottom of Form \| \| --- \| --- \| | 10828932 | AMYLIN PHARMACEUTICALS INC | VAN CAUTER EVE | |
| The disclosure provides, among other things, the use of GLP-1 receptor agonist compounds to treat obstructive sleep apnea. The GLP-1 receptor agonist compounds may be exendins, exendin analogs, GLP-1(7-37), GLP-1 (7-37) analogs (e.g., GLP-1 (7-36)-NH2) and th like. The GLP-1 receptor agonist compound may be exenatide. | | | | |
| 18. [P2012000176](https://patentscope.wipo.int/search/en/detail.jsf?docId=do74429321&recNum=18&office=&queryString=FP%3Aanalog*+AND+PA%3ALilly+AND+PD%3A%28%5B01.01.1994+to+01.01.2015%5D%29+&prevFilter=&sortOption=Pub+Date+Desc&maxRec=494) **ANALOGO PEPTIDICO DE OXINTOMODULINA** | | | do | 31.08.2012 |
| \|  \|  \| \| --- \| --- \| | 2012000176 | ELI LILLY AND COMPANY | WAYNE DAVID KOHN | |
| La presente invención se refiere a un análogo peptídico de Oxintomodulina empleado en el tratamiento de diabetes y/u obesidad. | | | | |
| 19. [P2012000175](https://patentscope.wipo.int/search/en/detail.jsf?docId=do74429320&recNum=19&office=&queryString=FP%3Aanalog*+AND+PA%3ALilly+AND+PD%3A%28%5B01.01.1994+to+01.01.2015%5D%29+&prevFilter=&sortOption=Pub+Date+Desc&maxRec=494) **ANALOGO PEPTIDICO DE OXINTOMODULINA** | | | do | 31.08.2012 |
| \|  \|  \| \| --- \| --- \| | 2012000175 | ELI LILLY AND COMPANY | WAYNE DAVID KOHN | |
| La presente invención se refiere a un análogo peptídico de Oxintomodulina empleado en el tratamiento de diabetes y/u obesidad. | | | | |
| 20. [220161](https://patentscope.wipo.int/search/en/detail.jsf?docId=il74427803&recNum=20&office=&queryString=FP%3Aanalog*+AND+PA%3ALilly+AND+PD%3A%28%5B01.01.1994+to+01.01.2015%5D%29+&prevFilter=&sortOption=Pub+Date+Desc&maxRec=494) **OXYNTOMODULIN PEPTIDE ANALOGUE** | | | il | 31.07.2012 |
| \| A61K / \| Top of Form  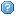  Bottom of Form \| \| --- \| --- \| | 220161 | ELI LILLY AND COMPANY |  | |
|  | | | | |
| 21. [181430](https://patentscope.wipo.int/search/en/detail.jsf?docId=sg90471038&recNum=21&office=&queryString=FP%3Aanalog*+AND+PA%3ALilly+AND+PD%3A%28%5B01.01.1994+to+01.01.2015%5D%29+&prevFilter=&sortOption=Pub+Date+Desc&maxRec=494) **OXYNTOMODULIN PEPTIDE ANALOGUE** | | | sg | 30.07.2012 |
| \| A61K 38/17 \| Top of Form  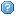  Bottom of Form \| \| --- \| --- \| | 2012036844 | ELI LILLY AND COMPANY | ALSINA-FERNANDEZ, JORGE | |
| ABSTRACT The present invention provides an Oxyntomodulin peptide analogue useful in the treatment of diabetes and/or obesity. | | | | |
| 22. [181872](https://patentscope.wipo.int/search/en/detail.jsf?docId=sg90471749&recNum=22&office=&queryString=FP%3Aanalog*+AND+PA%3ALilly+AND+PD%3A%28%5B01.01.1994+to+01.01.2015%5D%29+&prevFilter=&sortOption=Pub+Date+Desc&maxRec=494) **OXYNTOMODULIN PEPTIDE ANALOGUE** | | | sg | 30.07.2012 |
| \| A61K 38/17 \| Top of Form  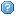  Bottom of Form \| \| --- \| --- \| | 2012046223 | ELI LILLY AND COMPANY | ALSINA-FERNANDEZ, JORGE | |
| The present invention provides Oxyntomodulin peptide analogues useful in the treatment of diabetes and/or obesity. | | | | |
| 23. [2435061](https://patentscope.wipo.int/search/en/detail.jsf?docId=EP45574856&recNum=23&office=&queryString=FP%3Aanalog*+AND+PA%3ALilly+AND+PD%3A%28%5B01.01.1994+to+01.01.2015%5D%29+&prevFilter=&sortOption=Pub+Date+Desc&maxRec=494) **GLP-1 RECEPTOR AGONIST COMPOUNDS FOR SLEEP ENHANCEMENT** | | | EP | 04.04.2012 |
| \| A61K 38/00 \| Top of Form  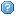  Bottom of Form \| \| --- \| --- \| | 10781185 | AMYLIN PHARMACEUTICALS INC | BASS JOSEPH T | |
| The disclosure provides, among other things, the use of GLP-1 receptor agonist compounds to enhance sleep, increase the duration and/or intensity of non-rapid eye movement (NREM) sleep, treat NREM sleep disorders, and to treat circadian rhythm sleep disorders. The GLP-1 receptor agonist compounds may be exendins, exendin analogs, GLP-1(7-37), GLP-1(7-37) analogs (e.g., GLP-1(7-36)-NH2) and the like. In one embodiment, the GLP-1 receptor agonist compound is exenatide. | | | | |
| 24. [079344](https://patentscope.wipo.int/search/en/detail.jsf?docId=ar44919976&recNum=24&office=&queryString=FP%3Aanalog*+AND+PA%3ALilly+AND+PD%3A%28%5B01.01.1994+to+01.01.2015%5D%29+&prevFilter=&sortOption=Pub+Date+Desc&maxRec=494) **ANALOGO PEPTIDICO DE OXINTOMODULINA, COMPOSICION FARMACEUTICA QUE LO COMPRENDE Y USO PARA PREPARAR UN MEDICAMENTO UTIL PARA TRATAR DIABETES NO INSULINODEPENDIENTE Y/U OBESIDAD** | | | ar | 18.01.2012 |
| \| A61K 38/26 \| Top of Form  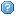  Bottom of Form \| \| --- \| --- \| | P100104554 | ELI LILLY AND COMPANY |  | |
| Análogo peptídico de Oxintomodulina que comprende la secuencia de aminoácido: His-(D-Ser)-Gln-Gly-Thr-Phe-Thr-Ser-Asp-(1-Nal)-Ser-Lys-Tyr-Leu-Asp-Glu-Lys-Ala-Ala-Gln-Glu-Phe-Val-Gln-Trp-Leu-Leu-Asn-(Aib)-Ala-Arg-Asn-Arg-Asn-Asn-Ile-Ala-Xaa38-Xaa39 (SEC ID Ns 5) en donde Xaa38 es Cys, Cys-PEG, o está ausente; Xaa39 es Cys, Cys-PEG, o está ausente; y en donde el aminoácido C-terminales opcionalmente amidado o la secuencia de aminoácido: His-(D-Ser)-Gln-Gly-Thr-Phe-Thr-Ser-Asp-(1-Nal)-Ser-Lys-Tyr-Leu-Asp-Glu-Lys-Ala-Ala-Gln-Glu-Phe-Val-Gln-Trp-Leu-Leu-Asn-(Aib)-Ala-Arg-Asn-Arg-Asn-Asn-Ile-Ala-Cys-Cys (SEC ID Ns 2). Composición farmacéutica que lo comprende. Su uso para preparar un medicamento útil para tratar diabetes no insulinodependiente y/u obesidad. | | | | |
| 25. [079345](https://patentscope.wipo.int/search/en/detail.jsf?docId=ar44919978&recNum=25&office=&queryString=FP%3Aanalog*+AND+PA%3ALilly+AND+PD%3A%28%5B01.01.1994+to+01.01.2015%5D%29+&prevFilter=&sortOption=Pub+Date+Desc&maxRec=494) **ANALOGO PEPTIDICO DE OXINTOMODULINA** | | | ar | 18.01.2012 |
| \| A61K 38/26 \| Top of Form  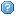  Bottom of Form \| \| --- \| --- \| | P100104555 | ELI LILLY AND COMPANY |  | |
| Análogo peptídico de Oxintomodulina que comprende la secuencia de aminoácido: His-(Aib)-Gln-Gly-Thi-Phe-Tbr-Ser-Asp-Tvr-Ser-Lys-Tyr-Leu-Asp-Ser-Lys-LvsA1a-G1n-G1u-Phe -Va1-G1n-Trp-Leu-Leu-Asn-(Aib)-G1y-Ar-Asn-Ar-Asn-Asn- I1eA1a- Xaa8-Xaag (SEÇ ID NO: 5) en donde Xaa38 es Cys, Cys-PEG, o está ausente; Xaa39 es Cys, Cys-PEG, o está ausente; y en donde el aminoácido C-terminales opcionalmente amidado o la secuencia de aminoácido: His-(Aib)-GIn-G1v-Thr-Phe-Thr-Sel-Asp-Tyr-Ser-Lys-Tvr-LeuAsp-Ser-LysLvs A1a-GIn-Glu-Phe-Va1-GIn-Trp-Leu-Leu-Asn-(Aib)-G1y-Ar-Asn-Ar-Asn-Asn Ile-Ala-Cvs-C'vs (SEÇ 1D NO: ) Composición farmacéutica que lo comprende. Su uso para preparar un medicamento útil para tratar para tratar diabetes no insulinodependiente u obesidad. | | | | |
| 26. [2371072](https://patentscope.wipo.int/search/en/detail.jsf?docId=es32290573&recNum=26&office=&queryString=FP%3Aanalog*+AND+PA%3ALilly+AND+PD%3A%28%5B01.01.1994+to+01.01.2015%5D%29+&prevFilter=&sortOption=Pub+Date+Desc&maxRec=494) **PROTEINAS DE FUSION ANALOGAS DE GLP-1.** | | | es | 27.12.2011 |
| \| C07K 14/605 \| Top of Form  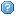  Bottom of Form \| \| --- \| --- \| | 04752589 | ELI LILLY AND COMPANY | GLAESNER, Wolfgang | |
|  | | | | |
| 27. [1641823](https://patentscope.wipo.int/search/en/detail.jsf?docId=PT108252651&recNum=27&office=&queryString=FP%3Aanalog*+AND+PA%3ALilly+AND+PD%3A%28%5B01.01.1994+to+01.01.2015%5D%29+&prevFilter=&sortOption=Pub+Date+Desc&maxRec=494) **GLP-1 ANALOG FUSION PLROTEINS** | | | PT | 08.11.2011 |
| \| C07K 14/605 \| Top of Form  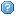  Bottom of Form \| \| --- \| --- \| | 04752589 | LILLY CO ELI | GLAESNER WOLFGANG | |
| The invention provides specific GLP-1 analogs fused to specific IgG4-Fc derivatives. These fusion proteins have an increased half-life, decreased immunogenicity, and reduce effector activity. The fusion proteins are useful in treating diabetes, obesity, irritable bowel syndrome and other conditions that would be benefited by lowering plasma glucose, inhibiting gastric and/or intestinal motility and inhibiting gastric and/or intestinal emptying, or inhibiting food intake. | | | | |
| 28. [WO/2011/119657](https://patentscope.wipo.int/search/en/detail.jsf?docId=WO2011119657&recNum=28&office=&queryString=FP%3Aanalog*+AND+PA%3ALilly+AND+PD%3A%28%5B01.01.1994+to+01.01.2015%5D%29+&prevFilter=&sortOption=Pub+Date+Desc&maxRec=494) **NOVEL PEPTIDES AND METHODS FOR THEIR PREPARATION AND USE** | | | WO | 29.09.2011 |
| \| [A61K 38/16](http://www.wipo.int/ipcpub/?symbol=A61K0038160000&refresh=page&viewmode=a&notes=no&headings=no&showdeleted=no) \| Top of Form  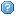  Bottom of Form \| \| --- \| --- \| | PCT/US2011/029501 | ELI LILLY AND COMPANY | ALSINA-FERNANDEZ, Jorge | |
| The present invention is in the field of treatment of diabetes and relates to peptides that exhibit activity for both glucose-dependent insulinotropic peptide receptor (GIP-R) and glucagon-like peptide- 1 receptor (GLP-1-R) and are selective over glucagon receptor (Gluc-R). Specifically provided are GIP analogs with amino acid substitutions introduced to modulate activity for both GIP-R and GLP-1-R and maintain selectivity over Gluc-R. | | | | |
| 29. [2794664](https://patentscope.wipo.int/search/en/detail.jsf?docId=CA94577791&recNum=29&office=&queryString=FP%3Aanalog*+AND+PA%3ALilly+AND+PD%3A%28%5B01.01.1994+to+01.01.2015%5D%29+&prevFilter=&sortOption=Pub+Date+Desc&maxRec=494) **NOVEL PEPTIDES AND METHODS FOR THEIR PREPARATION AND USE** | | | CA | 29.09.2011 |
| \| A61K 38/16 \| Top of Form  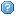  Bottom of Form \| \| --- \| --- \| | 2794664 | ELI LILLY AND COMPANY | ALSINA-FERNANDEZ, JORGE | |
| The present invention is in the field of treatment of diabetes and relates to peptides that exhibit activity for both glucose-dependent insulinotropic peptide receptor (GIP-R) and glucagon-like peptide- 1 receptor (GLP-1-R) and are selective over glucagon receptor (Gluc-R). Specifically provided are GIP analogs with amino acid substitutions introduced to modulate activity for both GIP-R and GLP-1-R and maintain selectivity over Gluc-R. | | | | |
| 30. [2368909](https://patentscope.wipo.int/search/en/detail.jsf?docId=EP29999492&recNum=30&office=&queryString=FP%3Aanalog*+AND+PA%3ALilly+AND+PD%3A%28%5B01.01.1994+to+01.01.2015%5D%29+&prevFilter=&sortOption=Pub+Date+Desc&maxRec=494) **GLP-1 analog fusion proteins** | | | EP | 28.09.2011 |
| \| C07K 14/605 \| Top of Form  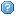  Bottom of Form \| \| --- \| --- \| | 11166548 | LILLY CO ELI | GLAESNER WOLFGANG | |
| The invention provides specific GLP-1 analogs fused to specific IgG4-Fc derivatives. These fusion proteins have an increased half-life, decreased immunogenicity, and reduced effector activity. The fusion proteins are useful in treating diabetes, obesity, irritable bowel syndrome and other conditions that would be benefited by lowering plasma glucose, inhibiting gastric and/or intestinal motility and inhibiting gastric and/or intestinal emptying, or inhibiting food intake. | | | | |
| 31. [WO/2011/087671](https://patentscope.wipo.int/search/en/detail.jsf?docId=WO2011087671&recNum=31&office=&queryString=FP%3Aanalog*+AND+PA%3ALilly+AND+PD%3A%28%5B01.01.1994+to+01.01.2015%5D%29+&prevFilter=&sortOption=Pub+Date+Desc&maxRec=494) **OXYNTOMODULIN PEPTIDE ANALOGUE** | | | WO | 21.07.2011 |
| \| [A61K 38/17](http://www.wipo.int/ipcpub/?symbol=A61K0038170000&refresh=page&viewmode=a&notes=no&headings=no&showdeleted=no) \| Top of Form  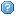  Bottom of Form \| \| --- \| --- \| | PCT/US2010/060380 | ELI LILLY AND COMPANY | ALSINA-FERNANDEZ, Jorge | |
| The present invention provides Oxyntomodulin peptide analogues useful in the treatment of diabetes and/or obesity. | | | | |
| 32. [WO/2011/087672](https://patentscope.wipo.int/search/en/detail.jsf?docId=WO2011087672&recNum=32&office=&queryString=FP%3Aanalog*+AND+PA%3ALilly+AND+PD%3A%28%5B01.01.1994+to+01.01.2015%5D%29+&prevFilter=&sortOption=Pub+Date+Desc&maxRec=494) **OXYNTOMODULIN PEPTIDE ANALOGUE** | | | WO | 21.07.2011 |
| \| [A61K 38/17](http://www.wipo.int/ipcpub/?symbol=A61K0038170000&refresh=page&viewmode=a&notes=no&headings=no&showdeleted=no) \| Top of Form  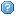  Bottom of Form \| \| --- \| --- \| | PCT/US2010/060390 | ELI LILLY AND COMPANY | ALSINA-FERNANDEZ, Jorge | |
| ABSTRACT The present invention provides an Oxyntomodulin peptide analogue useful in the treatment of diabetes and/or obesity. | | | | |
| 33. [2784671](https://patentscope.wipo.int/search/en/detail.jsf?docId=CA94560111&recNum=33&office=&queryString=FP%3Aanalog*+AND+PA%3ALilly+AND+PD%3A%28%5B01.01.1994+to+01.01.2015%5D%29+&prevFilter=&sortOption=Pub+Date+Desc&maxRec=494) **OXYNTOMODULIN PEPTIDE ANALOGUE** | | | CA | 21.07.2011 |
| \| C07K 14/575 \| Top of Form  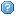  Bottom of Form \| \| --- \| --- \| | 2784671 | ELI LILLY AND COMPANY |  | |
| ABSTRACT The present invention provides an Oxyntomodulin peptide analogue useful in the treatment of diabetes and/or obesity. | | | | |
| 34. [2784668](https://patentscope.wipo.int/search/en/detail.jsf?docId=CA94560108&recNum=34&office=&queryString=FP%3Aanalog*+AND+PA%3ALilly+AND+PD%3A%28%5B01.01.1994+to+01.01.2015%5D%29+&prevFilter=&sortOption=Pub+Date+Desc&maxRec=494) **OXYNTOMODULIN PEPTIDE ANALOGUE** | | | CA | 21.07.2011 |
| \| C07K 14/575 \| Top of Form  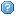  Bottom of Form \| \| --- \| --- \| | 2784668 | ELI LILLY AND COMPANY |  | |
| The present invention provides Oxyntomodulin peptide analogues useful in the treatment of diabetes and/or obesity. | | | | |
| 35. [WO/2011/056713](https://patentscope.wipo.int/search/en/detail.jsf?docId=WO2011056713&recNum=35&office=&queryString=FP%3Aanalog*+AND+PA%3ALilly+AND+PD%3A%28%5B01.01.1994+to+01.01.2015%5D%29+&prevFilter=&sortOption=Pub+Date+Desc&maxRec=494) **GLP-1 RECEPTOR AGONIST COMPOUNDS FOR OBSTRUCTIVE SLEEP APNEA** | | | WO | 12.05.2011 |
| \| [A61K 38/26](http://www.wipo.int/ipcpub/?symbol=A61K0038260000&refresh=page&viewmode=a&notes=no&headings=no&showdeleted=no) \| Top of Form  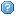  Bottom of Form \| \| --- \| --- \| | PCT/US2010/054553 | AMYLIN PHARMACEUTICALS, INC. | VAN CAUTER, Eve | |
| The disclosure provides, among other things, the use of GLP-1 receptor agonist compounds to treat obstructive sleep apnea. The GLP-1 receptor agonist compounds may be exendins, exendin analogs, GLP-1(7-37), GLP-1 (7-37) analogs (e.g., GLP-1 (7-36)-NH2) and th like. The GLP-1 receptor agonist compound may be exenatide. | | | | |
| 36. [WO/2010/138671](https://patentscope.wipo.int/search/en/detail.jsf?docId=WO2010138671&recNum=36&office=&queryString=FP%3Aanalog*+AND+PA%3ALilly+AND+PD%3A%28%5B01.01.1994+to+01.01.2015%5D%29+&prevFilter=&sortOption=Pub+Date+Desc&maxRec=494) **GLP-1 RECEPTOR AGONIST COMPOUNDS FOR SLEEP ENHANCEMENT** | | | WO | 02.12.2010 |
| \| [A61K 38/00](http://www.wipo.int/ipcpub/?symbol=A61K0038000000&refresh=page&viewmode=a&notes=no&headings=no&showdeleted=no) \| Top of Form  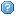  Bottom of Form \| \| --- \| --- \| | PCT/US2010/036326 | AMYLIN PHARMACEUTICALS, INC. | BASS, Joseph, T. | |
| The disclosure provides, among other things, the use of GLP-1 receptor agonist compounds to enhance sleep, increase the duration and/or intensity of non-rapid eye movement (NREM) sleep, treat NREM sleep disorders, and to treat circadian rhythm sleep disorders. The GLP-1 receptor agonist compounds may be exendins, exendin analogs, GLP-1(7-37), GLP-1(7-37) analogs (e.g., GLP-1(7-36)-NH2) and the like. In one embodiment, the GLP-1 receptor agonist compound is exenatide. | | | | |
| 37. [024755](https://patentscope.wipo.int/search/en/detail.jsf?docId=eg90314184&recNum=37&office=&queryString=FP%3Aanalog*+AND+PA%3ALilly+AND+PD%3A%28%5B01.01.1994+to+01.01.2015%5D%29+&prevFilter=&sortOption=Pub+Date+Desc&maxRec=494) **GLUCAGON-LIKE PEPTIDE -1 ANALOGS** | | | eg | 10.11.2010 |
| \| C07K 14/605 \| Top of Form  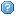  Bottom of Form \| \| --- \| --- \| | 2001060628 | ايلى ليلى اند كومبانى | وولفجانج جلاسنر | |
| DISCLOSED ARE GLUCAGON - LIKE PEPTID-1(GLP-1) COMPOUND WITH MODIFICATIONS AT ONE OR MORE OF THE FOLLOWING POSITIONS :11,12,16,22,23,24,25,27,30,33,34,35,36 OR 37 . METHOD OF TREATING A SUBJECT IN NEED OF GLP-1 RECEPTOR STIMULATION USING THESE GLP-1 COMPUNDS ARE ALSO DISCLOSED | | | | |
| 38. [024755](https://patentscope.wipo.int/search/en/detail.jsf?docId=EG90314184&recNum=38&office=&queryString=FP%3Aanalog*+AND+PA%3ALilly+AND+PD%3A%28%5B01.01.1994+to+01.01.2015%5D%29+&prevFilter=&sortOption=Pub+Date+Desc&maxRec=494) **GLUCAGON-LIKE PEPTIDE -1 ANALOGS** | | | EG | 10.11.2010 |
| \| C07K 14/605 \| Top of Form  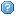  Bottom of Form \| \| --- \| --- \| | 2001060628 | ايلى ليلى اند كومبانى | وولفجانج جلاسنر | |
| DISCLOSED ARE GLUCAGON - LIKE PEPTID-1(GLP-1) COMPOUND WITH MODIFICATIONS AT ONE OR MORE OF THE FOLLOWING POSITIONS :11,12,16,22,23,24,25,27,30,33,34,35,36 OR 37 . METHOD OF TREATING A SUBJECT IN NEED OF GLP-1 RECEPTOR STIMULATION USING THESE GLP-1 COMPUNDS ARE ALSO DISCLOSED | | | | |
| 39. [1951659](https://patentscope.wipo.int/search/en/detail.jsf?docId=PT108253166&recNum=39&office=&queryString=FP%3Aanalog*+AND+PA%3ALilly+AND+PD%3A%28%5B01.01.1994+to+01.01.2015%5D%29+&prevFilter=&sortOption=Pub+Date+Desc&maxRec=494) **GLUCAGON RECEPTOR ANTAGONISTS, PREPARATION AND THERAPEUTIC USES** | | | PT | 10.09.2010 |
| \| C07C 235/42 \| Top of Form  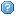  Bottom of Form \| \| --- \| --- \| | 06850138 | LILLY CO ELI | CONNER SCOTT EUGENE | |
| The present invention discloses novel compounds of Formula (I), or pharmaceutically acceptable salts thereof, which have glucagon receptor antagonist or inverse agonist activity, as well as methods for preparing such compounds. In another embodiment, the invention discloses pharmaceutical compositions comprising compounds of Formula (I) as well as methods of using them to treat diabetic and other glucagon related metabolic disorders, and the like. | | | | |
| 40. [2181712](https://patentscope.wipo.int/search/en/detail.jsf?docId=EP11140226&recNum=40&office=&queryString=FP%3Aanalog*+AND+PA%3ALilly+AND+PD%3A%28%5B01.01.1994+to+01.01.2015%5D%29+&prevFilter=&sortOption=Pub+Date+Desc&maxRec=494) **Use of glucagon-like peptide-1 or analogs to abolish catabolic changes after surgery** | | | EP | 05.05.2010 |
| \| A61K 38/00 \| Top of Form  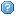  Bottom of Form \| \| --- \| --- \| | 08159135 | LILLY CO ELI | EFENDIC SUAD | |
| This invention provides a compound selected from GLP-1, GLP-1 analogues, GLP-1 derivatives, and pharmaceutically-acceptable salts thereof for use in the attenuation of post-surgical catabolic changes and insulin resistance. | | | | |
| 41. [2168982](https://patentscope.wipo.int/search/en/detail.jsf?docId=EP11126079&recNum=41&office=&queryString=FP%3Aanalog*+AND+PA%3ALilly+AND+PD%3A%28%5B01.01.1994+to+01.01.2015%5D%29+&prevFilter=&sortOption=Pub+Date+Desc&maxRec=494) **GLP-1 analog fusion protein formulations** | | | EP | 31.03.2010 |
| \| C07K 14/50 \| Top of Form  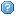  Bottom of Form \| \| --- \| --- \| | 09160182 | LILLY CO ELI | GLAESNER WOLFGANG | |
| The invention provides a stable solution formulation comprising a GLP-1-Fc fusion at a pH between about pH 6 and about pH 8.5. analogs fused to specific IgG4-Fc derivatives. These formulations provide unexpected and considerably greater chemical stability than when compared to GLP-1-Fc fusions at a pH outside the described ranges. The formulations comprising a GLP-1-Fc fusion are useful in treating diabetes, obesity, irritable bowel syndrome and other conditions that would be benefited by lowering plasma glucose, inhibiting gastric and/or intestinal motility and inhibiting gastric and/or intestinal emptying, or inhibiting food intake. | | | | |
| 42. [20100075299](https://patentscope.wipo.int/search/en/detail.jsf?docId=US43733438&recNum=42&office=&queryString=FP%3Aanalog*+AND+PA%3ALilly+AND+PD%3A%28%5B01.01.1994+to+01.01.2015%5D%29+&prevFilter=&sortOption=Pub+Date+Desc&maxRec=494) **Detection and use of antiviral resistance mutations** | | | US | 25.03.2010 |
| \| C12Q 1/70 \| Top of Form  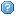  Bottom of Form \| \| --- \| --- \| | 12303942 | Bartholomeusz Angeline Ingrid | Bartholomeusz Angeline Ingrid | |
| The present invention relates generally to viral variants exhibiting reduced sensitivity to particular agents and/or reduced interactivity with immunological reagents. More particularly, the present invention is directed to hepatitis B virus (HBV) variants exhibiting complete or partial resistance to nucleoside or nucleotide analogs and/or reduced interactivity with antibodies to viral surface components including reduced sensitivity to these antibodies. Vaccines and diagnostic assays are also contemplated herein. | | | | |
| 43. [153453](https://patentscope.wipo.int/search/en/detail.jsf?docId=il4304539&recNum=43&office=&queryString=FP%3Aanalog*+AND+PA%3ALilly+AND+PD%3A%28%5B01.01.1994+to+01.01.2015%5D%29+&prevFilter=&sortOption=Pub+Date+Desc&maxRec=494) **GLUCAGON-LIKE PEPTIDE-1 ANALOGS AND USES THEREOF IN THE PREPARATION OF MEDICAMENTS FOR THE TREATMENT OF NON-INSULIN DEPENDENT DIABETES, OBESITY, STROKE, MYOCARDIAL INFARCTION, CATABOLIC CHANGES AFTER SURGERY AND IRRITABLE BOWEL SYNDROME** | | | il | 18.11.2009 |
| \|  \|  \| \| --- \| --- \| | 153453 | ELI LILLY & CO. |  | |
|  | | | | |
| 44. [2326906](https://patentscope.wipo.int/search/en/detail.jsf?docId=es5736537&recNum=44&office=&queryString=FP%3Aanalog*+AND+PA%3ALilly+AND+PD%3A%28%5B01.01.1994+to+01.01.2015%5D%29+&prevFilter=&sortOption=Pub+Date+Desc&maxRec=494) **FORMULACIONES DE PROTEINAS DE FUSION ANALOGAS AL GLP-1.** | | | es | 21.10.2009 |
| \| C07K 14/605 \| Top of Form  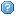  Bottom of Form \| \| --- \| --- \| | E05854150 | ELI LILLY AND COMPANY | GLAESNER, WOLFGANG | |
| Una formulación de disolución estable que comprende una cantidad terapéuticamente efectiva de una fusión GLP-1-Fc a un pH de entre aproximadamente un pH de 6 y aproximadamente un pH de 8,5 en la que la fusión GLP-1-Fc comprende un análogo de GLP-1 que comprende una secuencia seleccionada de entre: a) (ID de SEC Nº: 1) ** ver secuencia** en la que Xaa 8 está seleccionado de entre Gly y Val; b) (ID de SEC Nº: 2) ** ver secuencia** en la que Xaa 8 está seleccionado de entre Gly y Val; c) (ID de SEC Nº: 3) ** ver secuencia** en la que Xaa8 está seleccionado de entre Gly y Val; d) (ID de SEC Nº: 4) ** ver secuencia** en la que Xaa8 está seleccionado de entre Gly y Val; e) (ID de SEC Nº: 5) ** ver secuencia** en la que Xaa8 está seleccionado de entre Gly y Val; f) (ID de SEC Nº: 6) ** ver secuencia** en la que: en la que Xaa 8 está seleccionado de entre Gly y Val; fusionada con la porción Fc de una inmunoglobulina que comprende la secuencia de la ID de SEC Nº: 7 Xaa en la posición 16 es Pro o Glu; Xaa en la posición 17 es Phe, Val o Ala; Xaa en la posición 18 es Leu, Glu o Ala; Xaa en la posición 80 es Asn o Ala; y Xaa en la posición 230 es Lys o está ausente. | | | | |
| 45. [1831252](https://patentscope.wipo.int/search/en/detail.jsf?docId=PT108250122&recNum=45&office=&queryString=FP%3Aanalog*+AND+PA%3ALilly+AND+PD%3A%28%5B01.01.1994+to+01.01.2015%5D%29+&prevFilter=&sortOption=Pub+Date+Desc&maxRec=494) **GLP-1 ANALOG FUSION PROTEIN FORMULATIONS** | | | PT | 17.09.2009 |
| \| C07K 14/50 \| Top of Form  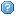  Bottom of Form \| \| --- \| --- \| | 05854150 | LILLY CO ELI | MILLICAN ROHN LEE JR | |
| The invention provides a stable solution formulation comprising a GLP-1-Fc fusion at a pH between about pH 6 and about pH 8.5. analogs fused to specific IgG4-Fc derivatives. These formulations provide unexpected and considerably greater chemical stability than when compared to GLP-1-Fc fusions at a pH outside the described ranges. The formulations comprising a GLP-1-Fc fusion are useful in treating diabetes, obesity, irritable bowel syndrome and other conditions that would be benefited by lowering plasma glucose, inhibiting gastric and/or intestinal motility and inhibiting gastric and/or intestinal emptying, or inhibiting food intake. | | | | |
| 46. [2325777](https://patentscope.wipo.int/search/en/detail.jsf?docId=es5725262&recNum=46&office=&queryString=FP%3Aanalog*+AND+PA%3ALilly+AND+PD%3A%28%5B01.01.1994+to+01.01.2015%5D%29+&prevFilter=&sortOption=Pub+Date+Desc&maxRec=494) **USO DE GLP-1 O ANALOGOS EN EL TRATAMIENTO DE ACCIDENTE CEREBROVASCULAR.** | | | es | 16.09.2009 |
| \| A61P 9/10 \| Top of Form  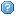  Bottom of Form \| \| --- \| --- \| | E99951570 | ELI LILLY AND COMPANY | EFENDIC, SUAD | |
| El uso de un compuesto seleccionado entre GLP-1, análogos de GLP-1, derivados de GLP-1 y sales farmacéuticamente aceptables de los mismos, en una cantidad efectiva en la fabricación de un medicamento para tratar pacientes en la fase aguda de un accidente cerebrovascular. | | | | |
| 47. [000060038735](https://patentscope.wipo.int/search/en/detail.jsf?docId=DE104197353&recNum=47&office=&queryString=FP%3Aanalog*+AND+PA%3ALilly+AND+PD%3A%28%5B01.01.1994+to+01.01.2015%5D%29+&prevFilter=&sortOption=Pub+Date+Desc&maxRec=494) **PSEUDOMYCIN ANALOGE** | | | DE | 02.07.2009 |
| \| C07K 7/06 \| Top of Form  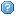  Bottom of Form \| \| --- \| --- \| | 60038735 | LILLY CO ELI | KULANTHAIVEL PALANIAPPAN | |
|  | | | | |
| 48. [2321439](https://patentscope.wipo.int/search/en/detail.jsf?docId=es5565620&recNum=48&office=&queryString=FP%3Aanalog*+AND+PA%3ALilly+AND+PD%3A%28%5B01.01.1994+to+01.01.2015%5D%29+&prevFilter=&sortOption=Pub+Date+Desc&maxRec=494) **ANALOGOS DEL PEPTIDO-1 SIMILAR A GLUCAGON.** | | | es | 05.06.2009 |
| \| A61K 38/26 \| Top of Form  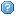  Bottom of Form \| \| --- \| --- \| | E06114553 | ELI LILLY & COMPANY | GLAESNER, WOLFGANG | |
| Un compuesto GLP-1 que comprende la secuencia de aminoácidos de la fórmula 1 (SEQ ID Nº 1) en la que: Xaa8 es: Gly o Val; Xaa 11 es: Asp, Glu, Arg, Thr, Ala, Lys, o His; Xaa12 es: His, Trp, Phe, o Tyr; Xaa 16 es: Leu, Ser, Thr, Trp, His, Phe, Asp, Val, Glu, o Ala; Xaa23 es: His, Asp, Lys, Glu, Gln o Arg; Xaa24 es: Glu, Arg, Ala, o Lys; Xaa26 es: Trp, Tyr, Phe, Asp, Lys, Glu, o His; Xaa27 es: Ala, Glu, His, Phe, Tyr, Trp, Arg, o Lys; Xaa 30 es: Ala, Glu, Asp, Ser, o His; Xaa33 es: Asp, Arg, Val, Lys, Ala, Gly, o Glu; Xaa 34 es: Glu, Lys, o Asp; Xaa35 es: Thr, Ser, Lys, Arg, Trp, Tyr, Phe, Asp, Gly, Pro, His, o Glu; Xaa 36 es: Thr, Ser, Asp, Trp, Tyr, Phe, Arg, Glu, o His; R es: Lys, Arg, Thr, Ser, Glu, Asp, Trp, Tyr, Phe, His, -NH2, Gly, Gly-Pro, o Gly-Pro-NH2, o está suprimido. | | | | |
| 49. [1695983](https://patentscope.wipo.int/search/en/detail.jsf?docId=PT108376758&recNum=49&office=&queryString=FP%3Aanalog*+AND+PA%3ALilly+AND+PD%3A%28%5B01.01.1994+to+01.01.2015%5D%29+&prevFilter=&sortOption=Pub+Date+Desc&maxRec=494) **GLUCAGON-LIKE PEPTIDE-1 ANALOGS** | | | PT | 05.05.2009 |
| \| A61K 38/00 \| Top of Form  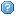  Bottom of Form \| \| --- \| --- \| | 06114553 | LILLY CO ELI | GLAESNER WOLFGANG | |
| Disclosed are glucagon-like peptide-1 (GLP-1) compounds with modifications at one or more of the following positions: 11, 12, 16, 22, 23, 24, 25, 27, 30, 33, 34, 35, 36, or 37. Methods of treating a subject in need of GLP-1 receptor stimulation using these GLP-1 compounds are also disclosed. | | | | |
| 50. [000069738615](https://patentscope.wipo.int/search/en/detail.jsf?docId=DE104536891&recNum=50&office=&queryString=FP%3Aanalog*+AND+PA%3ALilly+AND+PD%3A%28%5B01.01.1994+to+01.01.2015%5D%29+&prevFilter=&sortOption=Pub+Date+Desc&maxRec=494) **VERWENDUNG VON GLP-1 ODER ANALOGEN ZUR BEHANDLUNG VON MYOKARDISCHEM INFARKT** | | | DE | 30.04.2009 |
| \| A61K 38/26 \| Top of Form  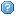  Bottom of Form \| \| --- \| --- \| | 69738615 | LILLY CO ELI | EFENDIC SUAD | |
|  | | | | |
| 51. [20090074769](https://patentscope.wipo.int/search/en/detail.jsf?docId=US43005047&recNum=51&office=&queryString=FP%3Aanalog*+AND+PA%3ALilly+AND+PD%3A%28%5B01.01.1994+to+01.01.2015%5D%29+&prevFilter=&sortOption=Pub+Date+Desc&maxRec=494) **GLP-1 analog fusion proteins** | | | US | 19.03.2009 |
| \| A61K 38/26 \| Top of Form  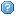  Bottom of Form \| \| --- \| --- \| | 12262832 | Eli Lilly and Company | Glaesner Wolfgang | |
| The invention provides specific GLP-1 analogs fused to specific IgG4-Fc derivatives. These fusion proteins have an increased half-life, decreased immunogenicity, and reduce effector activity. The fusion proteins are useful in treating diabetes, obesity, irritable bowel syndrome and other conditions that would be benefited by lowering plasma glucose, inhibiting gastric and/or intestinal motility and inhibiting gastric and/or intestinal emptying, or inhibiting food intake. | | | | |
| 52. [2029160](https://patentscope.wipo.int/search/en/detail.jsf?docId=EP15078646&recNum=52&office=&queryString=FP%3Aanalog*+AND+PA%3ALilly+AND+PD%3A%28%5B01.01.1994+to+01.01.2015%5D%29+&prevFilter=&sortOption=Pub+Date+Desc&maxRec=494) **METHODS TO RESTORE GLYCEMIC CONTROL** | | | EP | 04.03.2009 |
| \| A61K 38/22 \| Top of Form  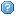  Bottom of Form \| \| --- \| --- \| | 07777061 | AMYLIN PHARMACEUTICALS INC | RABINOVITCH ALEX | |
| Provided herein are methods and compositions to achieve a sustained delay in the progression of, or an amelioration of diabetes in a subject, or a delay in diabetes onset in a subject at risk for diabetes, comprising an abbreviated course of administration of a pharmaceutical composition comprising an exendin or an exendin agonist analog in an amount effective to induce cell regeneration. | | | | |
| 53. [2304954](https://patentscope.wipo.int/search/en/detail.jsf?docId=es5724954&recNum=53&office=&queryString=FP%3Aanalog*+AND+PA%3ALilly+AND+PD%3A%28%5B01.01.1994+to+01.01.2015%5D%29+&prevFilter=&sortOption=Pub+Date+Desc&maxRec=494) **ANALOGOS DE PSEUDOMICINA.** | | | es | 01.11.2008 |
| \| C12P 21/04 \| Top of Form  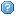  Bottom of Form \| \| --- \| --- \| | E00921593 | ELI LILLY AND COMPANY | KULANTHAIVEL, PALANIAPPAN | |
| Una pseudomicina A'' aislada que tiene la fórmula: (Ver fórmula) o una sal, hidrato o éster farmacéuticamente aceptable de la misma. | | | | |
| 54. [1951658](https://patentscope.wipo.int/search/en/detail.jsf?docId=EP14973928&recNum=54&office=&queryString=FP%3Aanalog*+AND+PA%3ALilly+AND+PD%3A%28%5B01.01.1994+to+01.01.2015%5D%29+&prevFilter=&sortOption=Pub+Date+Desc&maxRec=494) **GLUCAGON RECEPTOR ANTAGONISTS, PREPARATION AND THERAPEUTIC USES** | | | EP | 06.08.2008 |
| \| C07C 233/83 \| Top of Form  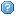  Bottom of Form \| \| --- \| --- \| | 06850148 | LILLY CO ELI | LI JIANKE | |
| The present invention discloses novel compounds of Formula I, or pharmaceutically acceptable salts thereof, which have glucagon receptor antagonist or inverse agonist activity, as well as methods for preparing such compounds. In another embodiment, the invention discloses pharmaceutical compositions comprising compounds of Formula I as well as methods of using them to treat diabetic and other glucagon related metabolic disorders, and the like. | | | | |
| 55. [1951659](https://patentscope.wipo.int/search/en/detail.jsf?docId=EP14973929&recNum=55&office=&queryString=FP%3Aanalog*+AND+PA%3ALilly+AND+PD%3A%28%5B01.01.1994+to+01.01.2015%5D%29+&prevFilter=&sortOption=Pub+Date+Desc&maxRec=494) **GLUCAGON RECEPTOR ANTAGONISTS, PREPARATION AND THERAPEUTIC USES** | | | EP | 06.08.2008 |
| \| C07C 235/42 \| Top of Form  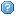  Bottom of Form \| \| --- \| --- \| | 06850138 | LILLY CO ELI | CHAPPELL MARK DONALD | |
| The present invention discloses novel compounds of Formula (I), or pharmaceutically acceptable salts thereof, which have glucagon receptor antagonist or inverse agonist activity, as well as methods for preparing such compounds. In another embodiment, the invention discloses pharmaceutical compositions comprising compounds of Formula (I) as well as methods of using them to treat diabetic and other glucagon related metabolic disorders, and the like. | | | | |
| 56. [2303343](https://patentscope.wipo.int/search/en/detail.jsf?docId=es5752062&recNum=56&office=&queryString=FP%3Aanalog*+AND+PA%3ALilly+AND+PD%3A%28%5B01.01.1994+to+01.01.2015%5D%29+&prevFilter=&sortOption=Pub+Date+Desc&maxRec=494) **USO DE GLP-1 O ANALOGOS EN EL TRATAMIENTO DEL INFARTO DE MIOCARDIO.** | | | es | 01.08.2008 |
| \| A61K 38/00 \| Top of Form  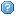  Bottom of Form \| \| --- \| --- \| | E97939579 | ELI LILLY AND COMPANY | EFENDIC, SUAD | |
| Uso de un compuesto seleccionado de GLP-1, análogos de GLP-1, derivados de GLP-1, y sus sales farmacéuticamente aceptables, para la preparación de una composición farmacéutico para el tratamiento de pacientes con un diagnóstico de infarto agudo de miocardio para normalizar la glucemia, en el que el compuesto se debe administrar a una dosis entre 0,25 y 6 pmol/kg de peso corporal/minuto. | | | | |
| 57. [1173471](https://patentscope.wipo.int/search/en/detail.jsf?docId=PT108213036&recNum=57&office=&queryString=FP%3Aanalog*+AND+PA%3ALilly+AND+PD%3A%28%5B01.01.1994+to+01.01.2015%5D%29+&prevFilter=&sortOption=Pub+Date+Desc&maxRec=494) **PSEUDOMYCIN ANALOGS** | | | PT | 21.07.2008 |
| \| C07K 7/06 \| Top of Form  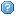  Bottom of Form \| \| --- \| --- \| | 00921593 | LILLY CO ELI | KULANTHAIVEL PALANIAPPAN | |
| The invention relates to pseudomycin natural products including pseudomycins A' and B', methods for making such pseudomycins, and methods employing antifungal activity of these pseudomycins. NMR and mass spectrometry indicate formula (IA) for pseudomycin A'. NMR and mass spectrometry indicate formula (IB) for pseudomycin B'. | | | | |
| 58. [964692](https://patentscope.wipo.int/search/en/detail.jsf?docId=PT108220008&recNum=58&office=&queryString=FP%3Aanalog*+AND+PA%3ALilly+AND+PD%3A%28%5B01.01.1994+to+01.01.2015%5D%29+&prevFilter=&sortOption=Pub+Date+Desc&maxRec=494) **USE OF GLP-1 OR ANALOGS IN TREATMENT OF MYOCARDIAL INFARCTION** | | | PT | 02.06.2008 |
| \| A61K 38/26 \| Top of Form  Bottom of Form \| \| --- \| --- \| | 97939579 | LILLY CO ELI | EFENDIC SUAD | |
| This invention provides a method of reducing mortality and morbidity after myocardial infarction. GLP-1, a GLP-1 analog, or a GLP-1 derivative, is administered at a dose effective to normalize blood glucose. | | | | |
| 59. [000060036199](https://patentscope.wipo.int/search/en/detail.jsf?docId=DE104195235&recNum=59&office=&queryString=FP%3Aanalog*+AND+PA%3ALilly+AND+PD%3A%28%5B01.01.1994+to+01.01.2015%5D%29+&prevFilter=&sortOption=Pub+Date+Desc&maxRec=494) **PROTEASERESISTENTE FLINT-ANALOGE** | | | DE | 21.05.2008 |
| \| C12N 15/12 \| Top of Form  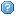  Bottom of Form \| \| --- \| --- \| | 60036199 | LILLY CO ELI | MICANOVIC RADMILA | |
|  | | | | |
| 60. [20080108560](https://patentscope.wipo.int/search/en/detail.jsf?docId=US42547837&recNum=60&office=&queryString=FP%3Aanalog*+AND+PA%3ALilly+AND+PD%3A%28%5B01.01.1994+to+01.01.2015%5D%29+&prevFilter=&sortOption=Pub+Date+Desc&maxRec=494) **Heterologous G-Csf Fusion Proteins** | | | US | 08.05.2008 |
| \| A61K 38/00 \| Top of Form  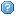  Bottom of Form \| \| --- \| --- \| | 10506455 | ELI LILLY AND COMPANY | Beals John Michael | |
| The present invention encompasses heterologous fusion 5 proteins comprising a hyperglycsoylated G-CSF analog fused to proteins such as albumin and the Fc portion of animmunoglobulin which act to extend the in vivo half-life of the protein compared to native G-CSF. These fusion proteins are particularly suited for the treatment of conditions 10 treatable by stimulation of circulating neutrophils, such as after chemotherapy regimens or in chronic congenital neutropenia. | | | | |
| 61. [2291197](https://patentscope.wipo.int/search/en/detail.jsf?docId=es5720755&recNum=61&office=&queryString=FP%3Aanalog*+AND+PA%3ALilly+AND+PD%3A%28%5B01.01.1994+to+01.01.2015%5D%29+&prevFilter=&sortOption=Pub+Date+Desc&maxRec=494) **ANALOGOS DE FLINT RESISTENTES A PROTEASAS.** | | | es | 01.03.2008 |
| \| A61K 38/17 \| Top of Form  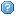  Bottom of Form \| \| --- \| --- \| | E00916264 | ELI LILLY AND COMPANY | MICANOVIC, RADMILA | |
| Un análogo de FLINT resistente a la proteolisis por una proteasa del tipo tripsina entre las posiciones 218 y 219 de SEQ ID nº. 1 o entre las posiciones 247 y 248 de SEQ ID nº. 3, y activo en la unión de Ligando de Fas (FasL) y/o LIGHT, en el que Arg en la posición 218 de SEQ ID nº. 1 o la posición 247 de SEQ ID nº. 3 está remplazado por Gln, y en el que el mencionado análogo es en como mínimo 97% idéntico a SEQ ID nº. 1 o SEQ ID nº. 3. | | | | |
| 62. [MX/a/2007/007565](https://patentscope.wipo.int/search/en/detail.jsf?docId=mx165567&recNum=62&office=&queryString=FP%3Aanalog*+AND+PA%3ALilly+AND+PD%3A%28%5B01.01.1994+to+01.01.2015%5D%29+&prevFilter=&sortOption=Pub+Date+Desc&maxRec=494) **GLP-1 ANALOG FUSION PROTEIN FORMULATIONS** | | | mx | 01.02.2008 |
| \| A61K 38/00 \| Top of Form  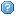  Bottom of Form \| \| --- \| --- \| | MX/a/2007/007565 | ELI LILLY AND COMPANY.* | Wolfgang Glaesner | |
| The invention provides a stable solution formulation comprising a GLP-1-Fc fusion at a pH between about pH 6 and about pH 8.5. analogs fused to specific IgG4-Fc derivatives. These formulations provide unexpected and considerably greater chemical stability than when compared to GLP-1-Fc fusions at a pH outside the described ranges. The formulations comprising a GLP-1-Fc fusion areuseful in treating diabetes, obesity, irritable bowel syndrome and other conditions that would be benefited by lowering plasma glucose, inhibiting gastric and/or intestinal motility and inhibiting gastric and/or intestinal emptying, or inhibiting food intake. | | | | |
| 63. [WO/2007/140522](https://patentscope.wipo.int/search/en/detail.jsf?docId=WO2007140522&recNum=63&office=&queryString=FP%3Aanalog*+AND+PA%3ALilly+AND+PD%3A%28%5B01.01.1994+to+01.01.2015%5D%29+&prevFilter=&sortOption=Pub+Date+Desc&maxRec=494) **DETECTION AND USE OF ANTIVIRAL RESISTANCE MUTATIONS** | | | WO | 13.12.2007 |
| \| [C12N 15/51](http://www.wipo.int/ipcpub/?symbol=C12N0015510000&refresh=page&viewmode=a&notes=no&headings=no&showdeleted=no) \| Top of Form  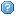  Bottom of Form \| \| --- \| --- \| | PCT/AU2007/000785 | MELBOURNE HEALTH | BARTHOLOMEUSZ, Angeline, Ingrid | |
| The present invention relates generally to viral variants exhibiting reduced sensitivity to particular agents and/or reduced interactivity with immunological reagents. More particularly, the present invention is directed to hepatitis B virus (HBV) variants exhibiting complete or partial resistance to nucleoside or nucleotide analogs and/or reduced interactivity with antibodies to viral surface components including reduced sensitivity to these antibodies. Vaccines and diagnostic assays are also contemplated herein. | | | | |
| 64. [WO/2007/133778](https://patentscope.wipo.int/search/en/detail.jsf?docId=WO2007133778&recNum=64&office=&queryString=FP%3Aanalog*+AND+PA%3ALilly+AND+PD%3A%28%5B01.01.1994+to+01.01.2015%5D%29+&prevFilter=&sortOption=Pub+Date+Desc&maxRec=494) **METHODS TO RESTORE GLYCEMIC CONTROL** | | | WO | 22.11.2007 |
| \| [A61K 38/22](http://www.wipo.int/ipcpub/?symbol=A61K0038220000&refresh=page&viewmode=a&notes=no&headings=no&showdeleted=no) \| Top of Form  Bottom of Form \| \| --- \| --- \| | PCT/US2007/011641 | AMYLIN PHARMACEUTICALS, INC. | RABINOVITCH, Alex | |
| Provided herein are methods and compositions to achieve a sustained delay in the progression of, or an amelioration of diabetes in a subject, or a delay in diabetes onset in a subject at risk for diabetes, comprising an abbreviated course of administration of a pharmaceutical composition comprising an exendin or an exendin agonist analog in an amount effective to induce cell regeneration. | | | | |
| 65. [WO/2007/123581](https://patentscope.wipo.int/search/en/detail.jsf?docId=WO2007123581&recNum=65&office=&queryString=FP%3Aanalog*+AND+PA%3ALilly+AND+PD%3A%28%5B01.01.1994+to+01.01.2015%5D%29+&prevFilter=&sortOption=Pub+Date+Desc&maxRec=494) **GLUCAGON RECEPTOR ANTAGONISTS, PREPARATION AND THERAPEUTIC USES** | | | WO | 01.11.2007 |
| \| [C07C 233/83](http://www.wipo.int/ipcpub/?symbol=C07C0233830000&refresh=page&viewmode=a&notes=no&headings=no&showdeleted=no) \| Top of Form  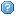  Bottom of Form \| \| --- \| --- \| | PCT/US2006/060857 | ELI LILLY AND COMPANY | LI, Jianke | |
| The present invention discloses novel compounds of Formula I, or pharmaceutically acceptable salts thereof, which have glucagon receptor antagonist or inverse agonist activity, as well as methods for preparing such compounds. In another embodiment, the invention discloses pharmaceutical compositions comprising compounds of Formula I as well as methods of using them to treat diabetic and other glucagon related metabolic disorders, and the like. | | | | |
| 66. [200701364](https://patentscope.wipo.int/search/en/detail.jsf?docId=ea95409446&recNum=66&office=&queryString=FP%3Aanalog*+AND+PA%3ALilly+AND+PD%3A%28%5B01.01.1994+to+01.01.2015%5D%29+&prevFilter=&sortOption=Pub+Date+Desc&maxRec=494) **GLP-1 ANALOG FUSION PROTEIN FORMULATIONS** | | | ea | 26.10.2007 |
| \| C07K 14/50 \| Top of Form  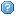  Bottom of Form \| \| --- \| --- \| | 200701364 | ЭЛИ ЛИЛЛИ ЭНД КОМПАНИ | Глеснер Вольфганг | |
| The invention provides a stable solution formulation comprising a GLP-1-Fc fusion at a pH between about pH 6 and about pH 8.5 analogs fused to specific IgG4-Fc derivatives. These formulations provide unexpected and considerably greater chemical stability than when compared to GLP-1-Fc fusions at a pH outside the described ranges. The formulations comprising a GLP-1-Fc fusion are useful in treating diabetes, obesity, irritable bowel syndrome and other conditions that would be benefited by lowering plasma glucose, inhibiting gastric and/or intestinal motility and inhibiting gastric and/or intestinal emptying, or inhibiting food intake. | | | | |
| 67. [WO/2007/114855](https://patentscope.wipo.int/search/en/detail.jsf?docId=WO2007114855&recNum=67&office=&queryString=FP%3Aanalog*+AND+PA%3ALilly+AND+PD%3A%28%5B01.01.1994+to+01.01.2015%5D%29+&prevFilter=&sortOption=Pub+Date+Desc&maxRec=494) **GLUCAGON RECEPTOR ANTAGONISTS, PREPARATION AND THERAPEUTIC USES** | | | WO | 11.10.2007 |
| \| [C07C 235/42](http://www.wipo.int/ipcpub/?symbol=C07C0235420000&refresh=page&viewmode=a&notes=no&headings=no&showdeleted=no) \| Top of Form  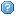  Bottom of Form \| \| --- \| --- \| | PCT/US2006/060769 | ELI LILLY AND COMPANY | CHAPPELL, Mark, Donald | |
| The present invention discloses novel compounds of Formula (I), or pharmaceutically acceptable salts thereof, which have glucagon receptor antagonist or inverse agonist activity, as well as methods for preparing such compounds. In another embodiment, the invention discloses pharmaceutical compositions comprising compounds of Formula (I) as well as methods of using them to treat diabetic and other glucagon related metabolic disorders, and the like. | | | | |
| 68. [101048173](https://patentscope.wipo.int/search/en/detail.jsf?docId=CN83237410&recNum=68&office=&queryString=FP%3Aanalog*+AND+PA%3ALilly+AND+PD%3A%28%5B01.01.1994+to+01.01.2015%5D%29+&prevFilter=&sortOption=Pub+Date+Desc&maxRec=494) **Extended glucagon-like peptide-1 analogs** | | | CN | 03.10.2007 |
| \| A61K 38/26 \| Top of Form  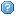  Bottom of Form \| \| --- \| --- \| | 03802038.6 | Lilly Co. Eli | Glaesner Wolfgang | |
| The invention encompasses GLP-1 peptides with modifications at various positions coupled with an extended C-terminus that provides increased stability. | | | | |
| 69. [101044162](https://patentscope.wipo.int/search/en/detail.jsf?docId=CN83233601&recNum=69&office=&queryString=FP%3Aanalog*+AND+PA%3ALilly+AND+PD%3A%28%5B01.01.1994+to+01.01.2015%5D%29+&prevFilter=&sortOption=Pub+Date+Desc&maxRec=494) **Glp-1 analog fusion protein formulations** | | | CN | 26.09.2007 |
| \| C07K 14/50 \| Top of Form  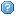  Bottom of Form \| \| --- \| --- \| | 200580035597.2 | Lilly Co. Eli | Glaesner Wolfgang | |
| The invention provides a stable solution formulation comprising a GLP-1-Fc fusion at a pH between about pH 6 and about pH 8.5. analogs fused to specific IgG4-Fc derivatives. These formulations provide unexpected and considerably greater chemical stability than when compared to GLP-1-Fc fusions at a pH outside the described ranges. The formulations comprising a GLP-1-Fc fusion are useful in treating diabetes, obesity, irritable bowel syndrome and other conditions that would be benefited by lowering plasma glucose, inhibiting gastric and/or intestinal motility and inhibiting gastric and/or intestinal emptying, or inhibiting food intake. | | | | |
| 70. [20070219123](https://patentscope.wipo.int/search/en/detail.jsf?docId=US41839499&recNum=70&office=&queryString=FP%3Aanalog*+AND+PA%3ALilly+AND+PD%3A%28%5B01.01.1994+to+01.01.2015%5D%29+&prevFilter=&sortOption=Pub+Date+Desc&maxRec=494) **Selective N-acylation of A82846 glygopeptides analogs** | | | US | 20.09.2007 |
| \| A61K 38/00 \| Top of Form  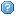  Bottom of Form \| \| --- \| --- \| | 10203533 | Eli Lilly and Company | Thompson Richard Craig | |
| The present invention provides a process for selectively acylating an A82846A, A82846B, A82846C or PA-42867-A glycopeptide at the N1, N2 or N3 positions and the monoacylated compounds prepared therefrom. | | | | |
| 71. [183285](https://patentscope.wipo.int/search/en/detail.jsf?docId=il4368363&recNum=71&office=&queryString=FP%3Aanalog*+AND+PA%3ALilly+AND+PD%3A%28%5B01.01.1994+to+01.01.2015%5D%29+&prevFilter=&sortOption=Pub+Date+Desc&maxRec=494) **GLP-1 ANALOG FUSION PROTEIN FORMULATIONS** | | | il | 20.09.2007 |
| \| C07K / \| Top of Form  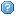  Bottom of Form \| \| --- \| --- \| | 183285 | ELI LILLY AND COMPANY |  | |
|  | | | | |
| 72. [000060124710](https://patentscope.wipo.int/search/en/detail.jsf?docId=DE104218477&recNum=72&office=&queryString=FP%3Aanalog*+AND+PA%3ALilly+AND+PD%3A%28%5B01.01.1994+to+01.01.2015%5D%29+&prevFilter=&sortOption=Pub+Date+Desc&maxRec=494) **ANALOGE DES GLUCAGON ÄHNLICHEN PEPTID-1** | | | DE | 13.09.2007 |
| \| C07K 14/605 \| Top of Form  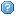  Bottom of Form \| \| --- \| --- \| | 60124710 | LILLY CO ELI | GLAESNER WOLFGANG | |
| Disclosed are glucagon-like peptide-1 (GLP-1) compounds with modifications at one or more of the following positions: 11, 12, 16, 22, 23, 24, 25, 27, 30, 33, 34, 35, 36, or 37. Methods of treating a subject in need of GLP-1 receptor stimulation using these GLP-1 compounds are also disclosed. | | | | |
| 73. [1831252](https://patentscope.wipo.int/search/en/detail.jsf?docId=EP14801943&recNum=73&office=&queryString=FP%3Aanalog*+AND+PA%3ALilly+AND+PD%3A%28%5B01.01.1994+to+01.01.2015%5D%29+&prevFilter=&sortOption=Pub+Date+Desc&maxRec=494) **GLP-1 ANALOG FUSION PROTEIN FORMULATIONS** | | | EP | 12.09.2007 |
| \| C07K 14/50 \| Top of Form  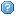  Bottom of Form \| \| --- \| --- \| | 05854150 | LILLY CO ELI | GLAESNER WOLFGANG | |
| The invention provides a stable solution formulation comprising a GLP-1-Fc fusion at a pH between about pH 6 and about pH 8.5. analogs fused to specific IgG4-Fc derivatives. These formulations provide unexpected and considerably greater chemical stability than when compared to GLP-1-Fc fusions at a pH outside the described ranges. The formulations comprising a GLP-1-Fc fusion are useful in treating diabetes, obesity, irritable bowel syndrome and other conditions that would be benefited by lowering plasma glucose, inhibiting gastric and/or intestinal motility and inhibiting gastric and/or intestinal emptying, or inhibiting food intake. | | | | |
| 74. [1020070089187](https://patentscope.wipo.int/search/en/detail.jsf?docId=kr1016643&recNum=74&office=&queryString=FP%3Aanalog*+AND+PA%3ALilly+AND+PD%3A%28%5B01.01.1994+to+01.01.2015%5D%29+&prevFilter=&sortOption=Pub+Date+Desc&maxRec=494) **GLP-1 ANALOG FUSION PROTEIN FORMULATIONS** | | | kr | 30.08.2007 |
| \| C07K 14/605 \| Top of Form  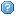  Bottom of Form \| \| --- \| --- \| | 1020077014068 | ELI LILLY AND COMPANY | GLAESNER WOLFGANG | |
| The invention provides a stable solution formulation comprising a GLP-1-Fc fusion at a pH between about pH 6 and about pH 8.5. analogs fused to specific IgG4-Fc derivatives. These formulations provide unexpected and considerably greater chemical stability than when compared to GLP-1-Fc fusions at a pH outside the described ranges. The formulations comprising a GLP-1-Fc fusion are useful in treating diabetes, obesity, irritable bowel syndrome and other conditions that would be benefited by lowering plasma glucose, inhibiting gastric and/or intestinal motility and inhibiting gastric and/or intestinal emptying, or inhibiting food intake.  © KIPO & WIPO 2007 | | | | |
| 75. [128740](https://patentscope.wipo.int/search/en/detail.jsf?docId=il4364992&recNum=75&office=&queryString=FP%3Aanalog*+AND+PA%3ALilly+AND+PD%3A%28%5B01.01.1994+to+01.01.2015%5D%29+&prevFilter=&sortOption=Pub+Date+Desc&maxRec=494) **USE OF GLUCAGON-LIKE PEPTIDE - 1 (GLP-1) OR ANALOGS THEREOF IN THE PREPARATION OF A PHARMACEUTICAL COMPOSITION FOR THE TREATMENT OF STRESS - INDUCED HYPERGLYCEMIA** | | | il | 19.08.2007 |
| \|  \|  \| \| --- \| --- \| | 128740 | ELI LILLY AND COMPANY |  | |
|  | | | | |
| 76. [2275685](https://patentscope.wipo.int/search/en/detail.jsf?docId=es5713073&recNum=76&office=&queryString=FP%3Aanalog*+AND+PA%3ALilly+AND+PD%3A%28%5B01.01.1994+to+01.01.2015%5D%29+&prevFilter=&sortOption=Pub+Date+Desc&maxRec=494) **ANALOGOS DEL PEPTIDO SIMILAR A GLUCAGON 1.** | | | es | 16.06.2007 |
| \| C07K 14/605 \| Top of Form  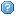  Bottom of Form \| \| --- \| --- \| | E01939252 | ELI LILLY AND COMPANY | GLAESNER, WOLFGANG | |
| Un compuesto GLP-1 seleccionado entre: Val8-Glu22-GLP-1(7-37)OH (ID SEC Nº 5) y Val8-Glu22-GLP-1(7-36)NH2 (ID SEC Nº 32). | | | | |
| 77. [1294757](https://patentscope.wipo.int/search/en/detail.jsf?docId=PT108231286&recNum=77&office=&queryString=FP%3Aanalog*+AND+PA%3ALilly+AND+PD%3A%28%5B01.01.1994+to+01.01.2015%5D%29+&prevFilter=&sortOption=Pub+Date+Desc&maxRec=494) **GLUCAGON-LIKE PEPTIDE-1 ANALOGS** | | | PT | 28.02.2007 |
| \| A61K 38/00 \| Top of Form  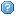  Bottom of Form \| \| --- \| --- \| | 01939252 | LILLY CO ELI | GLAESNER WOLFGANG | |
| Disclosed are glucagon-like peptide-1 (GLP-1) compounds with modifications at one or more of the following positions: 11, 12, 16, 22, 23, 24, 25, 27, 30, 33, 34, 35, 36, or 37. Methods of treating these GLP-1 compounds are also disclosed. | | | | |
| 78. [20070036806](https://patentscope.wipo.int/search/en/detail.jsf?docId=US41815678&recNum=78&office=&queryString=FP%3Aanalog*+AND+PA%3ALilly+AND+PD%3A%28%5B01.01.1994+to+01.01.2015%5D%29+&prevFilter=&sortOption=Pub+Date+Desc&maxRec=494) **GLP-1 analog fusion proteins** | | | US | 15.02.2007 |
| \| A61K 38/26 \| Top of Form  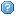  Bottom of Form \| \| --- \| --- \| | 10558627 | Eli Lilly and Company | Glaesner Wolfgang | |
| The invention provides specific GLP-1 analogs fused to specific IgG4-Fc derivatives. These fusion proteins have an increased half-life, decreased immunogenicity, and reduce effector activity. The fusion proteins are useful in treating diabetes, obesity, irritable bowel syndrome and other conditions that would be benefited by lowering plasma glucose, inhibiting gastric and/or intestinal motility and inhibiting gastric and/or intestinal emptying, or inhibiting food intake. | | | | |
| 79. [2264124](https://patentscope.wipo.int/search/en/detail.jsf?docId=es5591328&recNum=79&office=&queryString=FP%3Aanalog*+AND+PA%3ALilly+AND+PD%3A%28%5B01.01.1994+to+01.01.2015%5D%29+&prevFilter=&sortOption=Pub+Date+Desc&maxRec=494) **ACILACION SELECTIVA DE GRUPOS EPSILON-AMINO DE INSULINA.** | | | es | 16.12.2006 |
| \| C07K 14/62 \| Top of Form  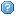  Bottom of Form \| \| --- \| --- \| | E95308167 | ELI LILLY AND COMPANY | BAKER, JEFFREY CLAYTON | |
| LA INVENCION SE REFIERE A LA ACILACION DE PROTEINAS. MAS PARTICULARMENTE, LA INVENCION SE REFIERE A UN PROCESO DE UN SOLO PASO PARA ACILAR SELECTIVAMENTE EL GRUPO DE EPSILON-AMINO LIBRE DE LA INSULINA, DE UN ANALOGO DE LA INSULINA O DE LA PROINSULINA EN LA PRESENCIA DE UN GRUPO DE ALFA-AMINO LIBRE. | | | | |
| 80. [128741](https://patentscope.wipo.int/search/en/detail.jsf?docId=il4352482&recNum=80&office=&queryString=FP%3Aanalog*+AND+PA%3ALilly+AND+PD%3A%28%5B01.01.1994+to+01.01.2015%5D%29+&prevFilter=&sortOption=Pub+Date+Desc&maxRec=494) **USE OF GLP-1 OR ANALOGS AND DERIVATIVES THEREOF FOR PREPARATION OF PHARMACEUTICAL COMPOSITIONS AS AGENTS IN REDUCING MORBIDITY AND MORTALITY OF MYOCARDIAL INFARCTION** | | | il | 10.12.2006 |
| \|  \|  \| \| --- \| --- \| | 128741 | ELI LILLY AND COMPANY |  | |
|  | | | | |
| 81. [20060263849](https://patentscope.wipo.int/search/en/detail.jsf?docId=US41610611&recNum=81&office=&queryString=FP%3Aanalog*+AND+PA%3ALilly+AND+PD%3A%28%5B01.01.1994+to+01.01.2015%5D%29+&prevFilter=&sortOption=Pub+Date+Desc&maxRec=494) **Method of treating a subject suffering stroke comprising administering Glucagon-like peptide-1 analogs** | | | US | 23.11.2006 |
| \| A61K 38/00 \| Top of Form  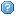  Bottom of Form \| \| --- \| --- \| | 11436457 | Eli Lilly and Company | Glaesner Wolfgang | |
| Disclosed are glucagon-like peptide-1 (GLP-1) compounds with modifications at one or more of the following positions: 11, 12, 16, 22, 23, 24, 25, 27, 30, 33, 34, 35, 36, or 37. Methods of treating a subject in need of GLP-1 receptor stimulation using these GLP-1 compounds are also disclosed. | | | | |
| 82. [20060252916](https://patentscope.wipo.int/search/en/detail.jsf?docId=US41581854&recNum=82&office=&queryString=FP%3Aanalog*+AND+PA%3ALilly+AND+PD%3A%28%5B01.01.1994+to+01.01.2015%5D%29+&prevFilter=&sortOption=Pub+Date+Desc&maxRec=494) **Modified glucagon-like peptide-1 analogs** | | | US | 09.11.2006 |
| \| A61K 38/26 \| Top of Form  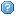  Bottom of Form \| \| --- \| --- \| | 10516490 | ELI LILLY AND COMPANY | DiMarchi Richard Dennis | |
| The invention encompasses GLP-1 compounds containing a GLP-1 peptide or a GLP-1 peptide with an extended C-terminus that is modified with a reactive group that is capable of forming covalent bonds with a blood component to form a conjugate. The conjugates may be formed in vivo or ex vivo. Methods of treating a subject in need of GLP-1 receptor stimulation using these GLP-1 compounds are also disclosed. | | | | |
| 83. [2006298938](https://patentscope.wipo.int/search/en/detail.jsf?docId=JP22707578&recNum=83&office=&queryString=FP%3Aanalog*+AND+PA%3ALilly+AND+PD%3A%28%5B01.01.1994+to+01.01.2015%5D%29+&prevFilter=&sortOption=Pub+Date+Desc&maxRec=494) **USE OF GLUCAGON-LIKE PEPTIDE-1 (GLP-1) OR ANALOG THEREOF TO PREVENT CATABOLIC CHANGE AFTER SURGERY** | | | JP | 02.11.2006 |
| \| A61P 43/00 \| Top of Form  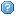  Bottom of Form \| \| --- \| --- \| | 2006199582 | ELI LILLY & CO | EFENDIC SUAD | |
| PROBLEM TO BE SOLVED: To provide a method of improving recovery after surgery by preventing the catabolic reaction and insulin resistance caused by surgical trauma.  SOLUTION: The method of attenuating post-surgical catabolic change and insulin resistance involves administering a compound selected from GLP-1, a GLP-1 analog, a GLP-1 derivative, and a pharmaceutically acceptable salt thereof to a patient requiring the attenuation.  COPYRIGHT: (C)2007,JPO&INPIT | | | | |
| 84. [1283051](https://patentscope.wipo.int/search/en/detail.jsf?docId=PT108230729&recNum=84&office=&queryString=FP%3Aanalog*+AND+PA%3ALilly+AND+PD%3A%28%5B01.01.1994+to+01.01.2015%5D%29+&prevFilter=&sortOption=Pub+Date+Desc&maxRec=494) **FORMULACOES DE INSULINA ESTAVEIS** | | | PT | 31.08.2006 |
| \| A61K 9/08 \| Top of Form  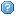  Bottom of Form \| \| --- \| --- \| | 02022956 | LILLY CO ELI | DEFELIPPIS MICHAEL ROSARIO | |
| The present invention provides a monomeric insulin analog formulation stabilized against aggregation in which the buffering agent is either TRIS or arginine. The stable formulations of the present invention are useful for treating diabetes, and are particularly advantageous in treatment regimes requiring lengthy chemical and physical stability, such as, in continuous infusion systems. | | | | |
| 85. [712862](https://patentscope.wipo.int/search/en/detail.jsf?docId=PT108334290&recNum=85&office=&queryString=FP%3Aanalog*+AND+PA%3ALilly+AND+PD%3A%28%5B01.01.1994+to+01.01.2015%5D%29+&prevFilter=&sortOption=Pub+Date+Desc&maxRec=494) **ACILACAO SELECTIVA DE GRUPOS EPSILON-AMINO EM ENSULINA** | | | PT | 31.08.2006 |
| \| A61K 38/28 \| Top of Form  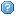  Bottom of Form \| \| --- \| --- \| | 95308167 | LILLY CO ELI |  | |
| The present invention relates to the acylation of proteins. More particularly, the invention relates to a one-step process for selectively acylating the free epsilon -amino group of insulin, insulin analog, or proinsulin in the presence of a free alpha -amino group. | | | | |
| 86. [1695983](https://patentscope.wipo.int/search/en/detail.jsf?docId=EP14596310&recNum=86&office=&queryString=FP%3Aanalog*+AND+PA%3ALilly+AND+PD%3A%28%5B01.01.1994+to+01.01.2015%5D%29+&prevFilter=&sortOption=Pub+Date+Desc&maxRec=494) **Glucagon-like peptide-1 analogs** | | | EP | 30.08.2006 |
| \| A61K 38/00 \| Top of Form  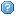  Bottom of Form \| \| --- \| --- \| | 06114553 | LILLY CO ELI | GLAESNER WOLFGANG | |
| Disclosed are glucagon-like peptide-1 (GLP-1) compounds with modifications at one or more of the following positions: 11, 12, 16, 22, 23, 24, 25, 27, 30, 33, 34, 35, 36, or 37. Methods of treating a subject in need of GLP-1 receptor stimulation using these GLP-1 compounds are also disclosed. | | | | |
| 87. [000060114567](https://patentscope.wipo.int/search/en/detail.jsf?docId=DE104209764&recNum=87&office=&queryString=FP%3Aanalog*+AND+PA%3ALilly+AND+PD%3A%28%5B01.01.1994+to+01.01.2015%5D%29+&prevFilter=&sortOption=Pub+Date+Desc&maxRec=494) **SELEKTIVE ACYLIERUNG VON A82846-GLYCOPEPTID-ANALOGE** | | | DE | 27.07.2006 |
| \| C07K 9/ \| Top of Form  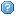  Bottom of Form \| \| --- \| --- \| | 60114567 | LILLY CO ELI | THOMPSON CRAIG | |
|  | | | | |
| 88. [1802386](https://patentscope.wipo.int/search/en/detail.jsf?docId=CN82992951&recNum=88&office=&queryString=FP%3Aanalog*+AND+PA%3ALilly+AND+PD%3A%28%5B01.01.1994+to+01.01.2015%5D%29+&prevFilter=&sortOption=Pub+Date+Desc&maxRec=494) **GLP-1 analog fusion plroteins** | | | CN | 12.07.2006 |
| \| C07K 19/00 \| Top of Form  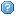  Bottom of Form \| \| --- \| --- \| | 200480015953.X | Lilly Co. Eli | Glaesner Wolfgang | |
| Disclosed are specific GLP-1 analogs fused to specific IgG4-Fc derivatives. These fusion proteins have an increased half-life, decreased immunogenicity, and reduce effector activity. The fusion proteins are useful in treating diabetes, obesity, irritable bowel syndrome and other conditions that would be benefited by lowering plasma glucose, inhibiting gastric and/or intestinal motility and inhibiting gastric and/or intestinal emptying, or inhibiting food intake. | | | | |
| 89. [200600015](https://patentscope.wipo.int/search/en/detail.jsf?docId=ea95404165&recNum=89&office=&queryString=FP%3Aanalog*+AND+PA%3ALilly+AND+PD%3A%28%5B01.01.1994+to+01.01.2015%5D%29+&prevFilter=&sortOption=Pub+Date+Desc&maxRec=494) **GLP-1 ANALOG FUSION PROTEINS** | | | ea | 30.06.2006 |
| \| C07K 14/605 \| Top of Form  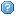  Bottom of Form \| \| --- \| --- \| | 200600015 | ЭЛИ ЛИЛЛИ ЭНД КОМПАНИ | Глеснер Вольфганг | |
| The invention provides specific GLP-1 analogs fused to specific IgG4-Fc derivatives. These fusion proteins have an increased half-life, decreased immunogenicity, and reduce effector activity. The fusion proteins are useful in treating diabetes, obesity, irritable bowel syndrome and other conditions that would be benefited by lowering plasma glucose, inhibiting gastric and/or intestinal motility and inhibiting gastric and/or intestinal emptying, or inhibiting food intake. | | | | |
| 90. [WO/2006/068910](https://patentscope.wipo.int/search/en/detail.jsf?docId=WO2006068910&recNum=90&office=&queryString=FP%3Aanalog*+AND+PA%3ALilly+AND+PD%3A%28%5B01.01.1994+to+01.01.2015%5D%29+&prevFilter=&sortOption=Pub+Date+Desc&maxRec=494) **GLP-1 ANALOG FUSION PROTEIN FORMULATIONS** | | | WO | 29.06.2006 |
| \| [C07K 14/50](http://www.wipo.int/ipcpub/?symbol=C07K0014500000&refresh=page&viewmode=a&notes=no&headings=no&showdeleted=no) \| Top of Form  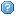  Bottom of Form \| \| --- \| --- \| | PCT/US2005/045376 | ELI LILLY AND COMPANY | GLAESNER, Wolfgang | |
| The invention provides a stable solution formulation comprising a GLP-1-Fc fusion at a pH between about pH 6 and about pH 8.5. analogs fused to specific IgG4-Fc derivatives. These formulations provide unexpected and considerably greater chemical stability than when compared to GLP-1-Fc fusions at a pH outside the described ranges. The formulations comprising a GLP-1-Fc fusion are useful in treating diabetes, obesity, irritable bowel syndrome and other conditions that would be benefited by lowering plasma glucose, inhibiting gastric and/or intestinal motility and inhibiting gastric and/or intestinal emptying, or inhibiting food intake. | | | | |
| 91. [2589647](https://patentscope.wipo.int/search/en/detail.jsf?docId=CA94281817&recNum=91&office=&queryString=FP%3Aanalog*+AND+PA%3ALilly+AND+PD%3A%28%5B01.01.1994+to+01.01.2015%5D%29+&prevFilter=&sortOption=Pub+Date+Desc&maxRec=494) **GLP-1 ANALOG FUSION PROTEIN FORMULATIONS** | | | CA | 29.06.2006 |
| \| C07K 14/50 \| Top of Form  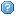  Bottom of Form \| \| --- \| --- \| | 2589647 | ELI LILLY AND COMPANY | GLAESNER, WOLFGANG | |
| The invention provides a stable solution formulation comprising a GLP-1-Fc fusion at a pH between about pH 6 and about pH 8.5. analogs fused to specific IgG4-Fc derivatives. These formulations provide unexpected and considerably greater chemical stability than when compared to GLP-1-Fc fusions at a pH outside the described ranges. The formulations comprising a GLP-1-Fc fusion are useful in treating diabetes, obesity, irritable bowel syndrome and other conditions that would be benefited by lowering plasma glucose, inhibiting gastric and/or intestinal motility and inhibiting gastric and/or intestinal emptying, or inhibiting food intake. | | | | |
| 92. [1780854](https://patentscope.wipo.int/search/en/detail.jsf?docId=CN82972541&recNum=92&office=&queryString=FP%3Aanalog*+AND+PA%3ALilly+AND+PD%3A%28%5B01.01.1994+to+01.01.2015%5D%29+&prevFilter=&sortOption=Pub+Date+Desc&maxRec=494) **Insulin analogs having protracted time action** | | | CN | 31.05.2006 |
| \| C07K 14/62 \| Top of Form  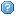  Bottom of Form \| \| --- \| --- \| | 200480011107.0 | Lilly Co. Eli | Dimarchi Richard Dennis | |
| The present invention provides the insulin analog A0 A21 B31 B32, which provides a protracted, even basal duration of action. The present invention also provides a method of treating diabetes mellitus comprising administering the insulin analog. | | | | |
| 93. [PA/a/2005/013565](https://patentscope.wipo.int/search/en/detail.jsf?docId=mx143162&recNum=93&office=&queryString=FP%3Aanalog*+AND+PA%3ALilly+AND+PD%3A%28%5B01.01.1994+to+01.01.2015%5D%29+&prevFilter=&sortOption=Pub+Date+Desc&maxRec=494) **GLP-1 ANALOG FUSION PLROTEINS** | | | mx | 16.05.2006 |
| \| A61K 38/26 \| Top of Form  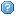  Bottom of Form \| \| --- \| --- \| | PA/a/2005/013565 | ELI LILLY AND COMPANY.* | Andrew Mark Vick | |
| The invention provides specific GLP-1 analogs fused to specific IgG4-Fc derivatives. These fusion proteins have an increased half-life, decreased immunogenicity, and reduce effector activity. The fusion proteins are useful in treating diabetes, obesity, irritable bowel syndrome and other conditions that would be benefited by lowering plasma glucose, inhibiting gastric and/or intestinal motility and inhibiting gastric and/or intestinal emptying, or inhibiting food intake. | | | | |
| 94. [1652531](https://patentscope.wipo.int/search/en/detail.jsf?docId=EP14511943&recNum=94&office=&queryString=FP%3Aanalog*+AND+PA%3ALilly+AND+PD%3A%28%5B01.01.1994+to+01.01.2015%5D%29+&prevFilter=&sortOption=Pub+Date+Desc&maxRec=494) **Use of GLP-1 or Analogues in Treatment of Stroke** | | | EP | 03.05.2006 |
| \| A61K 38/26 \| Top of Form  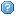  Bottom of Form \| \| --- \| --- \| | 05111027 | LILLY CO ELI | SAUD EFFENDIC | |
| This invention provides a method of reducing mortality and morbidity associated with stroke. GLP-1, a GLP-1 analogue, or a GLP-1 derivative is administered at a dose effective to normalize blood glucose. | | | | |
| 95. [WO/2006/044294](https://patentscope.wipo.int/search/en/detail.jsf?docId=WO2006044294&recNum=95&office=&queryString=FP%3Aanalog*+AND+PA%3ALilly+AND+PD%3A%28%5B01.01.1994+to+01.01.2015%5D%29+&prevFilter=&sortOption=Pub+Date+Desc&maxRec=494) **HUMAN PROTEIN C ANALOGS** | | | WO | 27.04.2006 |
| \| [C12N 9/64](http://www.wipo.int/ipcpub/?symbol=C12N0009640000&refresh=page&viewmode=a&notes=no&headings=no&showdeleted=no) \| Top of Form  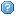  Bottom of Form \| \| --- \| --- \| | PCT/US2005/036310 | ELI LILLY AND COMPANY | SWANSON, Barbara, Anne | |
| This present invention provides human protein C analogs having increased activity as compared to wild-type human activated protein C. | | | | |
| 96. [171926](https://patentscope.wipo.int/search/en/detail.jsf?docId=il4358077&recNum=96&office=&queryString=FP%3Aanalog*+AND+PA%3ALilly+AND+PD%3A%28%5B01.01.1994+to+01.01.2015%5D%29+&prevFilter=&sortOption=Pub+Date+Desc&maxRec=494) **GLP-1 ANALOG FUSION PROTEINS** | | | il | 10.04.2006 |
| \| A61K 38//26 \| Top of Form  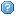  Bottom of Form \| \| --- \| --- \| | 171926 | ELI LILLY AND COMPANY |  | |
|  | | | | |
| 97. [1641823](https://patentscope.wipo.int/search/en/detail.jsf?docId=EP14509107&recNum=97&office=&queryString=FP%3Aanalog*+AND+PA%3ALilly+AND+PD%3A%28%5B01.01.1994+to+01.01.2015%5D%29+&prevFilter=&sortOption=Pub+Date+Desc&maxRec=494) **GLP-1 ANALOG FUSION PROTEINS** | | | EP | 05.04.2006 |
| \| C07K 14/605 \| Top of Form  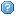  Bottom of Form \| \| --- \| --- \| | 04752589 | LILLY CO ELI | GLAESNER WOLFGANG | |
| The invention provides specific GLP-1 analogs fused to specific IgG4-Fc derivatives. These fusion proteins have an increased half-life, decreased immunogenicity, and reduce effector activity. The fusion proteins are useful in treating diabetes, obesity, irritable bowel syndrome and other conditions that would be benefited by lowering plasma glucose, inhibiting gastric and/or intestinal motility and inhibiting gastric and/or intestinal emptying, or inhibiting food intake. | | | | |
| 98. [1020060022262](https://patentscope.wipo.int/search/en/detail.jsf?docId=kr813857&recNum=98&office=&queryString=FP%3Aanalog*+AND+PA%3ALilly+AND+PD%3A%28%5B01.01.1994+to+01.01.2015%5D%29+&prevFilter=&sortOption=Pub+Date+Desc&maxRec=494) **GLP-1 ANALOG FUSION PLROTEINS** | | | kr | 09.03.2006 |
| \| C07K 14/605 \| Top of Form  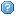  Bottom of Form \| \| --- \| --- \| | 1020057023668 | ELI LILLY AND COMPANY | GLAESNER WOLFGANG | |
| The invention provides specific GLP-1 analogs fused to specific IgG4- Fc derivatives. These fusion proteins have an increased half-life, decreased immunogenicity, and reduce effector activity. The fusion proteins are useful in treating diabetes, obesity, irritable bowel syndrome and other conditions that would be benefited by lowering plasma glucose, inhibiting gastric and/or intestinal motility and inhibiting gastric and/or intestinal emptying, or inhibiting food intake.  © KIPO & WIPO 2007 | | | | |
| 99. [1620465](https://patentscope.wipo.int/search/en/detail.jsf?docId=EP14473167&recNum=99&office=&queryString=FP%3Aanalog*+AND+PA%3ALilly+AND+PD%3A%28%5B01.01.1994+to+01.01.2015%5D%29+&prevFilter=&sortOption=Pub+Date+Desc&maxRec=494) **INSULIN ANALOGS HAVING PROTRACTED TIME ACTION** | | | EP | 01.02.2006 |
| \| A61K 38/28 \| Top of Form  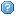  Bottom of Form \| \| --- \| --- \| | 04749928 | LILLY CO ELI | DIMARCHI RICHARD DENNIS | |
| The present invention provides the insulin analog A0Arg A21Gly B31Arg B32Arg, which provides a protracted, even basal duration of action. The present invention also provides a method of treating diabetes mellitus comprising administering the insulin analog. | | | | |
| 100. [20060014241](https://patentscope.wipo.int/search/en/detail.jsf?docId=US41510662&recNum=100&office=&queryString=FP%3Aanalog*+AND+PA%3ALilly+AND+PD%3A%28%5B01.01.1994+to+01.01.2015%5D%29+&prevFilter=&sortOption=Pub+Date+Desc&maxRec=494) **Extended glucagon-like peptide-1 analogs** | | | US | 19.01.2006 |
| \| A61K 38/26 \| Top of Form  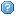  Bottom of Form \| \| --- \| --- \| | 10499111 | Eli Lilly and Company | Glaesner Wolfgang | |
| The invention encompasses GLP-1 peptides with modifications at various positions coupled with an extended C-terminus that provides increased stability. | | | | |
| 101. [1020050121748](https://patentscope.wipo.int/search/en/detail.jsf?docId=kr789852&recNum=101&office=&queryString=FP%3Aanalog*+AND+PA%3ALilly+AND+PD%3A%28%5B01.01.1994+to+01.01.2015%5D%29+&prevFilter=&sortOption=Pub+Date+Desc&maxRec=494) **INSULIN ANALOGS HAVING PROTRACTED TIME ACTION** | | | kr | 27.12.2005 |
| \| C07K 14/62 \| Top of Form  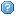  Bottom of Form \| \| --- \| --- \| | 1020057020584 | ELI LILLY AND COMPANY | DIMARCHI RICHARD DENNIS | |
| The present invention provides the insulin analog A0Arg A21Gly B31Arg B32Arg, which provides a protracted, even basal duration of action. The present invention also provides a method of treating diabetes mellitus comprising administering the insulin analog.  © KIPO & WIPO 2007 | | | | |
| 102. [000069533868](https://patentscope.wipo.int/search/en/detail.jsf?docId=DE104469816&recNum=102&office=&queryString=FP%3Aanalog*+AND+PA%3ALilly+AND+PD%3A%28%5B01.01.1994+to+01.01.2015%5D%29+&prevFilter=&sortOption=Pub+Date+Desc&maxRec=494) **Glucagon-ähnliche insulinotrope Peptid-Analoge, Zusammensetzungen und Verwendungsverfahren** | | | DE | 01.12.2005 |
| \| C12N 15/16 \| Top of Form  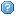  Bottom of Form \| \| --- \| --- \| | 69533868 | LILLY CO ELI | CHEN VICTOR JOHN | |
|  | | | | |
| 103. [1585959](https://patentscope.wipo.int/search/en/detail.jsf?docId=EP14415390&recNum=103&office=&queryString=FP%3Aanalog*+AND+PA%3ALilly+AND+PD%3A%28%5B01.01.1994+to+01.01.2015%5D%29+&prevFilter=&sortOption=Pub+Date+Desc&maxRec=494) **EXTENDED GLUCAGON-LIKE PEPTIDE-1 ANALOGS** | | | EP | 19.10.2005 |
| \| G01N 1/00 \| Top of Form  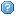  Bottom of Form \| \| --- \| --- \| | 03700026 | LILLY CO ELI | GLAESNER WOLFGANG | |
| The invention encompasses GLP-1 peptides with modifications at various positions coupled with an extended C-terminus that provides increased stability. | | | | |
| 104. [044776](https://patentscope.wipo.int/search/en/detail.jsf?docId=ar5313710&recNum=104&office=&queryString=FP%3Aanalog*+AND+PA%3ALilly+AND+PD%3A%28%5B01.01.1994+to+01.01.2015%5D%29+&prevFilter=&sortOption=Pub+Date+Desc&maxRec=494) **PROTEINAS DE FUSION ANALOGAS GLP-1** | | | ar | 05.10.2005 |
| \| C07K 14/605 \| Top of Form  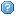  Bottom of Form \| \| --- \| --- \| | P040102037 | ELI LILLY AND COMPANY |  | |
| Se proporciona análogos GLP-1 específicos fusionados a derivados IgG4-Fc específicos. Estas proteínas de fusión tiene un período de vida media aumentado, una inmunogenicidad disminuida y una actividad efectora reducida. Las proteínas de fusión son útiles en los tratamientos para la diabetes, obesidad, síndrome del colon irritable y otros estados patológicos que podrían beneficiarse por medio de la baja de la glucosa en plasma, inhibiendo la motilidad gástrica y/o intestinal e inhibiendo la evacuación gástrica y/o intestinal, o inhibiendo la ingesta de alimentos. Reivindicación 1: Una proteína de fusión heteróloga que comprende un análogo GLP-1, que comprende una secuencia seleccionada del grupo que consiste de: a) (SEQ ID NO: 1) His-Xaa8-Glu-Gly-Thr-Phe-Thr-Ser-Asp-Val-Ser-Ser-Tyr-Leu-Glu-Glu-Gln-Ala-Ala-Lys-Glu-Phe-Ile-Ala-Trp-Leu-Val-Lys-Gly-Gly-Gly, donde Xaa8 es seleccionado de Gly y Val; b) (SEQ ID NO: 2) His-Xaa8-Glu-Gly-Thr-Phe-Thr-Ser-Asp-Val-Ser-Ser-Tyr-Leu-Glu-Glu-Gln-Ala-Ala-Lys-Glu-Phe-Ile-Ala-Trp-Leu-Lys-Asn-Gly-Gly-Gly, donde Xaa8 es seleccionado de Gly y Val: c) (SEQ ID NO: 3) His-Xaa8-Glu-Gly-Thr-Phe-Thr-Ser-Asp-Val-Ser-Ser-Tyr-Leu-Glu-Glu-Gln-Ala-Ala-Lys-Glu-Phe-Ile-Ala-Trp-Leu-Val-Lys-Gly-Gly-Pro, donde Xaa8 es seleccionado de Gly y Val; d) (SEQ ID NO: 4) His-Xaa8-Glu-Gly-Thr-Phe-Thr-Ser-Asp-Val-Ser-Ser-Tyr-Leu-Glu-Glu-Gln-Ala-Ala-Lys-Glu-Phe-Ile-Ala-Trp-Leu-Lys-Asn-Gly-Gly-Pro, donde Xaa8 es seleccionado de Gly y Val; e) (SEQ ID NO: 5) His-Xaa8-Glu-Gly-Thr-Phe-Thr-Ser-Asp-Val-Ser-Ser-Tyr-Leu-Glu-Glu-Gln-Ala-Ala-Lys-Glu-Phe-Ile-Ala-Trp-Leu-Val-Lys-Gly-Gly, donde Xaa8 es seleccionado de Gly y Val; f) (SEQ ID NO: 6) His-Xaa8-Glu-Gly-Thr-Phe-Thr-Ser-Asp-Val-Ser-Ser-Tyr-Leu-Glu-Glu-Gln-Ala-Ala-Lys-Glu-Phe-Ile-Ala-Trp-Leu-Lys-Asn-Gly-Gly, donde Xaa8 es seleccionado de Gly y Val; fusionado a la porción Fc de una inmunoglobulina que comprende la secuencia de SEQ ID IN: 7 Ala-Glu-Ser-Lys-Tyr-Gly-Pro-Pro-Cys-Pro-Pro-Cys-Pro-Ala-Pro-Xaa16-Xaa17-Xaa18-Gly-Gly-Pro-Ser-Val-Phe-Leu-Phe-Pro-Pro-Lys-Pro-Lys-Asp-Thr-Leu-Met-Ile-Ser-Arg-Thr-Pro-Glu-Val-Thr-Cys-Val-Val- Val-Asp-Val-Ser-Gln-Glu-Asp-Pro-Glu-Val-Gln-Phe-Asn-Trp-Tyr-Val-Asp-Gly-Val-Glu-Val-His-Asn-Ala-Lys-Thr-Lys-Pro-Arg-Glu-Glu-Gln-Phe-Xaa80-Ser-Thr-Tyr-Arg-Val-Val-Ser-Val-Leu-Thr-Val-Leu-His-Gln-Asp-Trp-Leu-Asn-Gly-Lys-Glu-Tyr-Lys-Cys-Lys-Val-Ser-Asn- Lys-Gly-Leu-Pro-Ser-Ser-Ile-Glu-Lys-Thr-Ile-Ser-Lys-Ala-Lys-Gly-Gln-Pro-Arg-Glu-Pro-Gln-Val-Tyr-Thr-Leu-Pro-Pro-Ser-Gln-Glu-Glu-Met-Thr-Lys-Asn-Gln-Val-Ser-Leu-Thr-Cys-Leu-Val-Lys-Gly-Phe-Tyr-Pro-Ser-Asp-Ile-Ala-Val-Glu-Trp-Glu-Ser-Asn-Gly-Gln-Pro- Glu-Asn-Asn-Tyr-Lys-Thr-Thr-Pro-Pro-Val-Leu-Asp-Ser-Asp-Gly-Ser-Phe-Phe-Leu-Tyr-Ser-Arg-Leu-Thr-Val-Asp-Lys-Ser-Arg-Trp-Gln-Glu-Gly-Asn-Val-Phe-Ser-Cys-Ser-Val-Met-His-Glu-Ala-Leu-His-Asn-His-Tyr-Thr-Gln-Lys-Ser-Leu-Ser-Leu-Ser-Leu-Gly-Xaa230 (SEQ IDNO: 7), donde: Xaa en la posición 16 es Pro o Glu; Xaa en la posición 17 es Phe, Val, o Ala; Xaa en la posición 18 es Leu, Glu, o Ala; Xaa en la p[truncated...] | | | | |
| 105. [1575490](https://patentscope.wipo.int/search/en/detail.jsf?docId=EP14405374&recNum=105&office=&queryString=FP%3Aanalog*+AND+PA%3ALilly+AND+PD%3A%28%5B01.01.1994+to+01.01.2015%5D%29+&prevFilter=&sortOption=Pub+Date+Desc&maxRec=494) **MODIFIED GLUCAGON-LIKE PEPTIDE-1 ANALOGS** | | | EP | 21.09.2005 |
| \| A61K 38/26 \| Top of Form  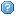  Bottom of Form \| \| --- \| --- \| | 03734046 | LILLY CO ELI | DIMARCHI RICHARD DENNIS | |
| The invention encompasses GLP-1 compounds containing a GLP-1 peptide or a GLP-1 peptide with an extended C-terminus that is modified with a reactive group that is capable of forming covalent bonds with a blood component to form a conjugate. The conjugates may be formed in vivo or ex vivo. Methods of treating a subject in need of GLP-1 receptor stimulation using these GLP-1 compounds are also disclosed. | | | | |
| 106. [1572936](https://patentscope.wipo.int/search/en/detail.jsf?docId=EP14379624&recNum=106&office=&queryString=FP%3Aanalog*+AND+PA%3ALilly+AND+PD%3A%28%5B01.01.1994+to+01.01.2015%5D%29+&prevFilter=&sortOption=Pub+Date+Desc&maxRec=494) **HETEROLOGOUS G-CSF FUSION PROTEINS** | | | EP | 14.09.2005 |
| \| C12N 1/00 \| Top of Form  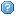  Bottom of Form \| \| --- \| --- \| | 03744099 | LILLY CO ELI | BEALS JOHN MICHAEL | |
| The present invention encompasses heterologous fusion proteins comprising a hyperglycsoylated G-CSF analog fusedto proteins such as albumin and the Fc portion of animmunoglobulin which act to extend the in vivo half-life ofthe protein compared to native G-CSF. These fusion proteinsare particularly suited for the treatment of conditions treatable by stimulation of circulating neutrophils, such as after chemotherapy regimens or in chronic congenitalneutropenia. | | | | |
| 107. [1566180](https://patentscope.wipo.int/search/en/detail.jsf?docId=EP14368569&recNum=107&office=&queryString=FP%3Aanalog*+AND+PA%3ALilly+AND+PD%3A%28%5B01.01.1994+to+01.01.2015%5D%29+&prevFilter=&sortOption=Pub+Date+Desc&maxRec=494) **Use of GLP-1 or Analogs in Treatment of Myocardial Infarction** | | | EP | 24.08.2005 |
| \| A61K 38/00 \| Top of Form  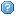  Bottom of Form \| \| --- \| --- \| | 04104453 | LILLY CO ELI | EFENDIC SUAD | |
| This invention provides a method of reducing mortality and morbidity after myocardial infarction. GLP-1, a GLP-1 analog, or a GLP-1 derivative, is administered at a dose effective to normalize blood glucose. | | | | |
| 108. [874625](https://patentscope.wipo.int/search/en/detail.jsf?docId=PT108211962&recNum=108&office=&queryString=FP%3Aanalog*+AND+PA%3ALilly+AND+PD%3A%28%5B01.01.1994+to+01.01.2015%5D%29+&prevFilter=&sortOption=Pub+Date+Desc&maxRec=494) **DERIVADOS DE INDANO PARA COMPOSICOES ANTIPSICOTICAS** | | | PT | 29.07.2005 |
| \| C07D 295/12 \| Top of Form  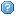  Bottom of Form \| \| --- \| --- \| | 97903904 | LILLY CO ELI | WARD JOHN S | |
| The present invention provides novel indane-like compounds which can be useful for treating psychosis and other conditions associated with the modulation of a muscarinic receptor. The invention provides formulations and methods for using the novel compounds. | | | | |
| 109. [2232820](https://patentscope.wipo.int/search/en/detail.jsf?docId=es5536900&recNum=109&office=&queryString=FP%3Aanalog*+AND+PA%3ALilly+AND+PD%3A%28%5B01.01.1994+to+01.01.2015%5D%29+&prevFilter=&sortOption=Pub+Date+Desc&maxRec=494) **ANALOGOS DE PEPTIDOS INSULINOTROPICOS DE TIPO GLUCAGONA, COMPOSICIONES Y PROCEDIMIENTOS DE USO.** | | | es | 01.06.2005 |
| \| A61P 3/08 \| Top of Form    Bottom of Form \| \| --- \| --- \| | E95307299 | ELI LILLY AND COMPANY | CHEN, VICTOR JOHN | |
| SE PRESENTAN ANALOGOS DE PEPTIDO (GLP-1 (7-37) INSULINOTROPICO COMO GLUCAGON Y DERIVADOS. LOS ANALOGOS COMPRENDEN SUSTITUCIONES DE AMINOACIDO, MODIFICACIONES TERMINALES DE AMINO O CARBONILO, Y ACILACIONES DE C{SUB,6-10}. LOS COMPUESTOS REIVINDICADOS ESTIMULAN LA SECRECION O BIOSINTESIS DE INSULINA EN CELULA BETA DE FUNCIONAIENTO POBRE Y SON POR LO TANTO UTILES EN EL TRATAMIENTO DE DIABETICOS DE TIPO II. | | | | |
| 110. [2233070](https://patentscope.wipo.int/search/en/detail.jsf?docId=es5692905&recNum=110&office=&queryString=FP%3Aanalog*+AND+PA%3ALilly+AND+PD%3A%28%5B01.01.1994+to+01.01.2015%5D%29+&prevFilter=&sortOption=Pub+Date+Desc&maxRec=494) **SINTESIS DE ANALOGOS PEPTIDICOS CICLICOS MODIFICADOS EN EL ANILLO.** | | | es | 01.06.2005 |
| \| A61K 9/72 \| Top of Form    Bottom of Form \| \| --- \| --- \| | E99942321 | ELI LILLY & COMPANY | BORROMEO, PETER, STANLEY | |
| Un proceso para modificar un núcleo de anillo peptídico cíclico que comprende las etapas de: (i) proporcionar un compuesto peptídico cíclico que comprende una unidad peptídica que tiene un grupo ó-hidro xilo y un grupo y-hidroxilo; (ii) abrir el anillo de dicho compuesto peptídico cíclico para proporcionar un primer péptido lineal en el que la unidad peptídica que tiene el grupo y-hidroxilo es la unidad peptídica N-terminal de dicho primer péptido lineal; (iii) separar por escisión dicha unidad peptídica que tiene un grupo y-hidroxilo para proporcionar un segundo péptido lineal; (iv) unir al menos un aminoácido, unidad dipeptídica o unidad sintética a dicho segundo péptido lineal para producir un tercer péptido lineal; (v) ciclar dicho tercer péptido lineal para producir un compuesto peptídico cíclico modificado que tiene un núcleo de anillo modificado. | | | | |
| 111. [2233070](https://patentscope.wipo.int/search/en/detail.jsf?docId=ES5692905&recNum=111&office=&queryString=FP%3Aanalog*+AND+PA%3ALilly+AND+PD%3A%28%5B01.01.1994+to+01.01.2015%5D%29+&prevFilter=&sortOption=Pub+Date+Desc&maxRec=494) **SINTESIS DE ANALOGOS PEPTIDICOS CICLICOS MODIFICADOS EN EL ANILLO.** | | | ES | 01.06.2005 |
| \| A61K 9/72 \| Top of Form    Bottom of Form \| \| --- \| --- \| | E99942321 | ELI LILLY & COMPANY | BORROMEO, PETER, STANLEY | |
| Un proceso para modificar un núcleo de anillo peptídico cíclico que comprende las etapas de: (i) proporcionar un compuesto peptídico cíclico que comprende una unidad peptídica que tiene un grupo ó-hidro xilo y un grupo y-hidroxilo; (ii) abrir el anillo de dicho compuesto peptídico cíclico para proporcionar un primer péptido lineal en el que la unidad peptídica que tiene el grupo y-hidroxilo es la unidad peptídica N-terminal de dicho primer péptido lineal; (iii) separar por escisión dicha unidad peptídica que tiene un grupo y-hidroxilo para proporcionar un segundo péptido lineal; (iv) unir al menos un aminoácido, unidad dipeptídica o unidad sintética a dicho segundo péptido lineal para producir un tercer péptido lineal; (v) ciclar dicho tercer péptido lineal para producir un compuesto peptídico cíclico modificado que tiene un núcleo de anillo modificado. | | | | |
| 112. [2232820](https://patentscope.wipo.int/search/en/detail.jsf?docId=ES5536900&recNum=112&office=&queryString=FP%3Aanalog*+AND+PA%3ALilly+AND+PD%3A%28%5B01.01.1994+to+01.01.2015%5D%29+&prevFilter=&sortOption=Pub+Date+Desc&maxRec=494) **ANALOGOS DE PEPTIDOS INSULINOTROPICOS DE TIPO GLUCAGONA, COMPOSICIONES Y PROCEDIMIENTOS DE USO.** | | | ES | 01.06.2005 |
| \| A61P 3/08 \| Top of Form    Bottom of Form \| \| --- \| --- \| | E95307299 | ELI LILLY AND COMPANY | CHEN, VICTOR JOHN | |
| SE PRESENTAN ANALOGOS DE PEPTIDO (GLP-1 (7-37) INSULINOTROPICO COMO GLUCAGON Y DERIVADOS. LOS ANALOGOS COMPRENDEN SUSTITUCIONES DE AMINOACIDO, MODIFICACIONES TERMINALES DE AMINO O CARBONILO, Y ACILACIONES DE C{SUB,6-10}. LOS COMPUESTOS REIVINDICADOS ESTIMULAN LA SECRECION O BIOSINTESIS DE INSULINA EN CELULA BETA DE FUNCIONAIENTO POBRE Y SON POR LO TANTO UTILES EN EL TRATAMIENTO DE DIABETICOS DE TIPO II. | | | | |
| 113. [1107981](https://patentscope.wipo.int/search/en/detail.jsf?docId=PT108354068&recNum=113&office=&queryString=FP%3Aanalog*+AND+PA%3ALilly+AND+PD%3A%28%5B01.01.1994+to+01.01.2015%5D%29+&prevFilter=&sortOption=Pub+Date+Desc&maxRec=494) **SINTESE DE ANALOGOS PEPTIDICOS CICLICOS DE ANEL MODIFICADO** | | | PT | 31.05.2005 |
| \| A61K 9/08 \| Top of Form    Bottom of Form \| \| --- \| --- \| | 99942321 | LILLY CO ELI | TURNER WILLIAM WILSON JR | |
| A method for modifying the cyclic peptide ring system of Echinocandin-type compounds to produce new analogs having antifungal activity is provided. The inventive process comprises opening the cyclic peptide ring, cleaving the terminal ornithine unit, inserting at least one new amino acid or other synthetic unit and closing the ring to produce a new cyclic peptide ring structure. The process allows one to incorporate features such as water-solubility into the cyclic peptide ring nucleus, sites for further modification, increase or decrease the number of amino acid or peptide units within the ring nucleus, and increase or decrease the total number of members within the ring. The invention further provides novel Echinocandin type compounds and their use as antifungal or anti-parasitic agents. | | | | |
| 114. [WO/2005/042733](https://patentscope.wipo.int/search/en/detail.jsf?docId=WO2005042733&recNum=114&office=&queryString=FP%3Aanalog*+AND+PA%3ALilly+AND+PD%3A%28%5B01.01.1994+to+01.01.2015%5D%29+&prevFilter=&sortOption=Pub+Date+Desc&maxRec=494) **HBV VARIANTS DETECTION AND APPLICATION** | | | WO | 12.05.2005 |
| \| [C12Q 1/70](http://www.wipo.int/ipcpub/?symbol=C12Q0001700000&refresh=page&viewmode=a&notes=no&headings=no&showdeleted=no) \| Top of Form    Bottom of Form \| \| --- \| --- \| | PCT/AU2004/001440 | MELBOURNE HEALTH | BARTHOLOMEUSZ, Angeline, Ingrid | |
| The present invention relates generally to viral variants exhibiting reduced sensitivity to particular agents and/or reduced interactivity with immunological reagents. More particularly, the present invention is directed to hepatitis B virus (HBV) variants exhibiting complete or partial resistance to nucleoside or nucleotide analogs and/or reduced interactivity with antibodies to viral surface components including reduced sensitivity to these antibodies. The present invention further contemplates assays for detecting such viral variants, which assays are useful in monitoring anti-viral therapeutic regimens and in developing new or modified vaccines directed against viral agents and in particular HBV variants. The present invention also contemplates the use of the viral variants to screen for and/or develop or design agents capable of inhibiting infection, replication and/or release of the virus. | | | | |
| 115. [PA/a/2004/006679](https://patentscope.wipo.int/search/en/detail.jsf?docId=mx124568&recNum=115&office=&queryString=FP%3Aanalog*+AND+PA%3ALilly+AND+PD%3A%28%5B01.01.1994+to+01.01.2015%5D%29+&prevFilter=&sortOption=Pub+Date+Desc&maxRec=494) **EXTENDED GLUCAGON-LIKE PEPTIDE-1 ANALOGS** | | | mx | 04.05.2005 |
| \| G01N 000/00000 \| Top of Form    Bottom of Form \| \| --- \| --- \| | PA/a/2004/006679 | ELI LILLY AND COMPANY | WOLFGANG GLAESNER | |
| The invention encompasses GLP-1 peptides with modifications at various positions coupled with an extended C-terminus that provides increased stability. | | | | |
| 116. [WO/2005/000892](https://patentscope.wipo.int/search/en/detail.jsf?docId=WO2005000892&recNum=116&office=&queryString=FP%3Aanalog*+AND+PA%3ALilly+AND+PD%3A%28%5B01.01.1994+to+01.01.2015%5D%29+&prevFilter=&sortOption=Pub+Date+Desc&maxRec=494) **GLP-1 ANALOG FUSION PLROTEINS** | | | WO | 06.01.2005 |
| \| [A61K 38/00](http://www.wipo.int/ipcpub/?symbol=A61K0038000000&refresh=page&viewmode=a&notes=no&headings=no&showdeleted=no) \| Top of Form    Bottom of Form \| \| --- \| --- \| | PCT/US2004/015595 | ELI LILLY AND COMPANY | GLAESNER, Wolfgang | |
| The invention provides specific GLP-1 analogs fused to specific IgG4-Fc derivatives. These fusion proteins have an increased half-life, decreased immunogenicity, and reduce effector activity. The fusion proteins are useful in treating diabetes, obesity, irritable bowel syndrome and other conditions that would be benefited by lowering plasma glucose, inhibiting gastric and/or intestinal motility and inhibiting gastric and/or intestinal emptying, or inhibiting food intake. | | | | |
| 117. [2528591](https://patentscope.wipo.int/search/en/detail.jsf?docId=CA94196808&recNum=117&office=&queryString=FP%3Aanalog*+AND+PA%3ALilly+AND+PD%3A%28%5B01.01.1994+to+01.01.2015%5D%29+&prevFilter=&sortOption=Pub+Date+Desc&maxRec=494) **GLP-1 ANALOG FUSION PROTEINS** | | | CA | 06.01.2005 |
| \| C07K 14/605 \| Top of Form    Bottom of Form \| \| --- \| --- \| | 2528591 | ELI LILLY AND COMPANY | GLAESNER, WOLFGANG | |
| The invention provides specific GLP-1 analogs fused to specific IgG4-Fc derivatives. These fusion proteins have an increased half-life, decreased immunogenicity, and reduce effector activity. The fusion proteins are useful in treating diabetes, obesity, irritable bowel syndrome and other conditions that would be benefited by lowering plasma glucose, inhibiting gastric and/or intestinal motility and inhibiting gastric and/or intestinal emptying, or inhibiting food intake. | | | | |
| 118. [038102](https://patentscope.wipo.int/search/en/detail.jsf?docId=ar5307039&recNum=118&office=&queryString=FP%3Aanalog*+AND+PA%3ALilly+AND+PD%3A%28%5B01.01.1994+to+01.01.2015%5D%29+&prevFilter=&sortOption=Pub+Date+Desc&maxRec=494) **ANALOGOS EXTENDIDOS DE PEPTIDO 1 DE TIPO GLUCAGON** | | | ar | 29.12.2004 |
| \| A61K 38/16 \| Top of Form    Bottom of Form \| \| --- \| --- \| | P030100014 | ELI LILLY AND COMPANY |  | |
| La invención incluye péptidos GLP-1 con modificaciones en diversas posiciones unidas a un terminación C extendida que proporcionan una estabilidad aumentada. Reivindicación 1: Un péptido GLP-1 extendido que comprende una secuencia aminoacídica de fórmula: Xaa7-Xaa8-Glu-Gly-Thr-Xaa12-Thr-Ser-Asp-Xaa16-Ser-Xaa18-Xaa19-Xaa20-Glu-Xaa22-GLn-Ala-Xaa25-Lys-Xaa27-Phe-Ile-Xaa30-Trp-Leu-Xaa33-Xaa34-Gly-Xaa36-Xaa37-Xaa38-Xaa39-Xaa40-Xaa41-Xaa42-Xaa43-Xaa44-Xaa45-Xaa46-Xaa47-Xaa48Xaa49-Xaa50 fórmula (1) (SEC N° ID 1) en al que: Xaa7 es: L-histidina, D-histidina, desaminohistidinina, 2-aminohistidina, beta-hidroxihistidina, homohistidina, alfa-fluorometilhistidina, o alfa-metilhistidina; Xaa8 es: Ala, Gly, Val, Leu, Ile, Ser o Thr; Xaa12 es: Phe, Trp o Tyr; Xaa16 es: Val, Trp Ile, Leu, Phe o Tyr; Xaa18 es: Ser, Trp, Tyr, Phe, Lys, Ile, Leu, Val; Xaa19 es: Tyr, Trp, o Phe; Xaa20 es: Leu, Phe, Tyr o Trp; Xaa22 es: Gly, Glu, Asp o Lys; Xaa25 es: Ala, Val, Ile, o Leu; Xaa27 es: Glu; Ile o Ala; Xaa30 es: Ala o Glu; Xaa33 es: Val o Ile; Xaa34 es: Lys, Asp, Arg o Glu; Xaa36 es: Gly, Pro o Arg; Xaa37 es: Gly, Pro o Ser; Xaa38 es: Ser, Pro o His; Xaa39 es: Ser, Arg, Thr, Trp o Lys; Xaa40 es: Ser o Gly; Xaa41 es: Ala, Asp, Arg, Glu, Lys o Gly; Xaa42 es: Pro, Ala, NH2 o está ausente; Xaa43 es: Pro, Ala, NH2 o está ausente; Xaa44 es: Pro, Ala, Arg, Lys, His, NH2 o está ausente; Xaa45 es: Ser, His, Pro, Lys, Arg, NH2 o está ausente; Xaa46 es: His, Ser, Arg, Lys, NH2 o está ausente; y Xaa47 es: His, Ser, Arg, Lys, NH2 o está ausente; con la condición de que si Xaa42, Xaa43, Xaa44, Xaa45, Xaa46, o Xaa47 está ausente, cada aminoácido cadena abajo está ausente, y con la condición además de que si Xaa36 es Agr y Xaa37 es Gly o Ser, el péptido GLP-1 no tiene la siguiente extensión de aminoácidos C-terminales empezando en Xaa38: Ser-Ser-Gly-Ala-Pro-Pro-Pro-Ser-NH2. Reivindicación 21: Un péptido GLP-1 extendido que comprende la secuencia aminoacídica de fórmula: Xaa7-Xaa8-Glu-Gly-Thr-Ser-Asp-Xaa16-Ser-Ser-Tyr-Lys-Glu-Xaa22-GLn-Ala-Xaa25-Lys-Glu-Phe-Ile-Ala-Trp-Leu-Xaa33-Xaa34-Gly-Xaa36-Xaa37-Xaa38-Xaa39-Xaa40-Xaa41-Xaa42-Xaa43-Xaa44-Xaa45-Xaa46-Xaa47; fórmula (3) (SEC N° ID 3) en al que: Xaa7 es: L-histidina, D-histidina, desaminohistidinina, 2-aminohistidina, beta-hidroxihistidina, homohistidina, alfa-fluorometilhistidina, o alfa-metilhistidina; Xaa8 es: Gly, Val, Leu, Ile, Ser o Thr; Xaa16 es: Val, Trp Ile, Leu, Phe o Tyr; Xaa22 es: Gly, Glu, Asp o Lys; Xaa25 es: Ala, Val, Ile, o Leu; Xaa33 es: Val o Ile; Xaa34 es: Lys, Asp, Arg o Glu; Xaa36 es: Gly Pro o Arg; Xaa37 es: Gly, Pro o Ser; Xaa38 es: Ser, Pro o His; Xaa39 es: Ser, Arg, Thr, Trp o Lys; Xaa40 es: Ser o Gly; Xaa41 es: Ala, Asp, Arg, Glu, Lys o Gly; Xaa42 es: Pro o Ala, NH2 o está ausente; Xaa43 es: Pro o Ala, NH2 o está ausente; Xaa44 es: Pro, Ala, Arg, Lys, His, NH2 o está ausente; Xaa45 es: Ser, His, Pro, Lys, Arg, NH2 o está ausente; Xaa46 es: His, Ser, Arg, Lys, NH2 o está ausente; y Xaa47 es: His, Ser, Arg, Lys, N[truncated...] | | | | |
| 119. [1481010](https://patentscope.wipo.int/search/en/detail.jsf?docId=EP14222302&recNum=119&office=&queryString=FP%3Aanalog*+AND+PA%3ALilly+AND+PD%3A%28%5B01.01.1994+to+01.01.2015%5D%29+&prevFilter=&sortOption=Pub+Date+Desc&maxRec=494) **ANTI-INTERLEUKIN-1 BETA ANALOGS** | | | EP | 01.12.2004 |
| \| A61K 39/395 \| Top of Form    Bottom of Form \| \| --- \| --- \| | 03707670 | LILLY CO ELI | BEALS JOHN MICHAEL | |
| The present invention encompasses analogs of humanized antibody Hu007 that neutralize IL−1&bgr; activity *in vivo*. These antibodies can be used to treat various diseases such as rheumatoid arthritis and osteoarthritis. | | | | |
| 120. [PA/a/2002/010263](https://patentscope.wipo.int/search/en/detail.jsf?docId=mx104321&recNum=120&office=&queryString=FP%3Aanalog*+AND+PA%3ALilly+AND+PD%3A%28%5B01.01.1994+to+01.01.2015%5D%29+&prevFilter=&sortOption=Pub+Date+Desc&maxRec=494) **PROCESS FOR PREPARING LIPID II** | | | mx | 12.11.2004 |
| \| C07H 15/00 \| Top of Form    Bottom of Form \| \| --- \| --- \| | PA/a/2002/010263 | ELI LILLY AND COMPANY* | BLASZCZAK, Larry, Chris | |
| A process is described for preparing a substrate for the transglycosylase enzymes of bacterial cell wall biosynthesis. The chemical synthesis makes available a sustainable and substantially pure source of supply of lipid II, including analogs thereof, that maybe used in the identification of new therapeutic agents capable of disrupting steps in bacterial cell wall biosynthesis. | | | | |
| 121. [WO/2004/096854](https://patentscope.wipo.int/search/en/detail.jsf?docId=WO2004096854&recNum=121&office=&queryString=FP%3Aanalog*+AND+PA%3ALilly+AND+PD%3A%28%5B01.01.1994+to+01.01.2015%5D%29+&prevFilter=&sortOption=Pub+Date+Desc&maxRec=494) **INSULIN ANALOGS HAVING PROTRACTED TIME ACTION** | | | WO | 11.11.2004 |
| \| [A61K 38/00](http://www.wipo.int/ipcpub/?symbol=A61K0038000000&refresh=page&viewmode=a&notes=no&headings=no&showdeleted=no) \| Top of Form    Bottom of Form \| \| --- \| --- \| | PCT/US2004/010960 | ELI LILLY AND COMPANY | DIMARCHI, Richard, Dennis | |
| The present invention provides the insulin analog A0Arg A21Gly B31Arg B32Arg, which provides a protracted, even basal duration of action. The present invention also provides a method of treating diabetes mellitus comprising administering the insulin analog. | | | | |
| 122. [2518776](https://patentscope.wipo.int/search/en/detail.jsf?docId=CA94164781&recNum=122&office=&queryString=FP%3Aanalog*+AND+PA%3ALilly+AND+PD%3A%28%5B01.01.1994+to+01.01.2015%5D%29+&prevFilter=&sortOption=Pub+Date+Desc&maxRec=494) **INSULIN ANALOGS HAVING PROTRACTED TIME ACTION** | | | CA | 11.11.2004 |
| \| C07K 14/62 \| Top of Form    Bottom of Form \| \| --- \| --- \| | 2518776 | ELI LILLY AND COMPANY | DIMARCHI, RICHARD DENNIS | |
| The present invention provides the insulin analog A0Arg A21Gly B31Arg B32Arg, which provides a protracted, even basal duration of action. The present invention also provides a method of treating diabetes mellitus comprising administering the insulin analog. | | | | |
| 123. [PA/a/2004/001525](https://patentscope.wipo.int/search/en/detail.jsf?docId=mx119824&recNum=123&office=&queryString=FP%3Aanalog*+AND+PA%3ALilly+AND+PD%3A%28%5B01.01.1994+to+01.01.2015%5D%29+&prevFilter=&sortOption=Pub+Date+Desc&maxRec=494) **GLUCAGON-LIKE PEPTIDE-1 ANALOGS** | | | mx | 26.08.2004 |
| \| C07C 000/00000 \| Top of Form    Bottom of Form \| \| --- \| --- \| | PA/a/2004/001525 | ELI LILLY AND COMPANY | WOLFGANG GLAESNER | |
| Disclosed are glucagon-like peptide-1 (GLP-1) compounds with modifications at one or more of the following positions:7, 8, 12, 16, 18, 19, 20, 22, 25, 27, 30, 33, and 37. Methods of treating a subject in need of GLP-1 receptor stimulation using these GLP-1 compounds are also disclosed. | | | | |
| 124. [160493](https://patentscope.wipo.int/search/en/detail.jsf?docId=il4332839&recNum=124&office=&queryString=FP%3Aanalog*+AND+PA%3ALilly+AND+PD%3A%28%5B01.01.1994+to+01.01.2015%5D%29+&prevFilter=&sortOption=Pub+Date+Desc&maxRec=494) **GLUCAGON-LIKE PEPTIDE-1 ANALOGS** | | | il | 25.07.2004 |
| \|  \|  \| \| --- \| --- \| | 160493 | ELI LILLY AND COMPANY |  | |
|  | | | | |
| 125. [20040132647](https://patentscope.wipo.int/search/en/detail.jsf?docId=US40450710&recNum=125&office=&queryString=FP%3Aanalog*+AND+PA%3ALilly+AND+PD%3A%28%5B01.01.1994+to+01.01.2015%5D%29+&prevFilter=&sortOption=Pub+Date+Desc&maxRec=494) **Amidated glucagon-like peptide-1** | | | US | 08.07.2004 |
| \| C07K 14/435 \| Top of Form    Bottom of Form \| \| --- \| --- \| | 10450042 | Eli Lilly and Company | DiMarchi Richard Dennis | |
| The present invention encompasses a GLP-**1** analog and compositions and formulations thereof useful for the treatment of hyperglycemia and other various diseases and conditions in mammals. | | | | |
| 126. [1432730](https://patentscope.wipo.int/search/en/detail.jsf?docId=EP14137088&recNum=126&office=&queryString=FP%3Aanalog*+AND+PA%3ALilly+AND+PD%3A%28%5B01.01.1994+to+01.01.2015%5D%29+&prevFilter=&sortOption=Pub+Date+Desc&maxRec=494) **GLUCAGON-LIKE PEPTIDE-1 ANALOGS** | | | EP | 30.06.2004 |
| \| C07K 14/605 \| Top of Form    Bottom of Form \| \| --- \| --- \| | 02756392 | LILLY CO ELI | GLAESNER WOLFGANG | |
| Disclosed are glucagon-like peptide-1 (GLP-1) compounds with modifications at one or more of the following positions: 7, 8, 12, 16, 18, 19, 20, 22, 25, 27, 30, 33, and 37. Methods of treating a subject in need of GLP-1 receptor stimulation using these GLP-1 compounds are also disclosed. | | | | |
| 127. [2208666](https://patentscope.wipo.int/search/en/detail.jsf?docId=es5528896&recNum=127&office=&queryString=FP%3Aanalog*+AND+PA%3ALilly+AND+PD%3A%28%5B01.01.1994+to+01.01.2015%5D%29+&prevFilter=&sortOption=Pub+Date+Desc&maxRec=494) **FRAGMENTOS DE PEPTIDO INSULINOTROPICO SIMILAR AL GLUCAGON BIOLOGICAMENTE ACTIVOS.** | | | es | 16.06.2004 |
| \| A61K 38/00 \| Top of Form    Bottom of Form \| \| --- \| --- \| | E95305963 | ELI LILLY AND COMPANY | JOHNSON, WILLIAM TERRY | |
| SE SUMINISTRAN FORMAS TRUNCADAS EN EL TERMINAL N DE UN PEPTIDO INSULINOTROPICO SIMILAR AL GLUCANON (GLP-1) Y ANALOGOS DEL MISMO. LOS POLIPEPTIDOS PRESENTADOS PROMUEVEN LA GLUCOSA PRODUCIDA POR LAS CELULAS PERO NO ESTIMULA LA EXPRESION A LA SECRECION DE INSULINA. LA INVENCION TAMBIEN SUMINISTRA METODOS PARA EL TRATAMIENTO DE LA DIABETES Y FORMULACIONES FARMACEUTICAS QUE COMPRENDEN LOS POLIPEPTIDOS PRESENTADOS. | | | | |
| 128. [2208666](https://patentscope.wipo.int/search/en/detail.jsf?docId=ES5528896&recNum=128&office=&queryString=FP%3Aanalog*+AND+PA%3ALilly+AND+PD%3A%28%5B01.01.1994+to+01.01.2015%5D%29+&prevFilter=&sortOption=Pub+Date+Desc&maxRec=494) **FRAGMENTOS DE PEPTIDO INSULINOTROPICO SIMILAR AL GLUCAGON BIOLOGICAMENTE ACTIVOS.** | | | ES | 16.06.2004 |
| \| A61K 38/00 \| Top of Form    Bottom of Form \| \| --- \| --- \| | E95305963 | ELI LILLY AND COMPANY | JOHNSON, WILLIAM TERRY | |
| SE SUMINISTRAN FORMAS TRUNCADAS EN EL TERMINAL N DE UN PEPTIDO INSULINOTROPICO SIMILAR AL GLUCANON (GLP-1) Y ANALOGOS DEL MISMO. LOS POLIPEPTIDOS PRESENTADOS PROMUEVEN LA GLUCOSA PRODUCIDA POR LAS CELULAS PERO NO ESTIMULA LA EXPRESION A LA SECRECION DE INSULINA. LA INVENCION TAMBIEN SUMINISTRA METODOS PARA EL TRATAMIENTO DE LA DIABETES Y FORMULACIONES FARMACEUTICAS QUE COMPRENDEN LOS POLIPEPTIDOS PRESENTADOS. | | | | |
| 129. [1423402](https://patentscope.wipo.int/search/en/detail.jsf?docId=EP14120186&recNum=129&office=&queryString=FP%3Aanalog*+AND+PA%3ALilly+AND+PD%3A%28%5B01.01.1994+to+01.01.2015%5D%29+&prevFilter=&sortOption=Pub+Date+Desc&maxRec=494) **NOVEL POLYPEPTIDE ANALOGS AND FUSIONS AND THEIR METHODS OF USE** | | | EP | 02.06.2004 |
| \| C07H 21/04 \| Top of Form    Bottom of Form \| \| --- \| --- \| | 02783970 | LILLY CO ELI | HEUER JOSEF GEORG | |
| Novel polypeptide analogs and fusion proteins of a transmembrane protein, LP276, are provided. Vectors and host cells directed to these polypeptides are provided. Additionally, methods of use are provided for the treatment or prevention of allergic autoimmune diseases, type 1 diabetes, inflammation, immunodeficiencies, cancers, and infectious diseases by administering an LP276 polypeptide, analogs and fusion proteins thereof to a patient in need of such therapy. | | | | |
| 130. [2002/10098](https://patentscope.wipo.int/search/en/detail.jsf?docId=za1397465&recNum=130&office=&queryString=FP%3Aanalog*+AND+PA%3ALilly+AND+PD%3A%28%5B01.01.1994+to+01.01.2015%5D%29+&prevFilter=&sortOption=Pub+Date+Desc&maxRec=494) **GLUCAGON-LIKE PEPTIDE-1 ANALOGS** | | | za | 26.05.2004 |
| \| C07K \| Top of Form    Bottom of Form \| \| --- \| --- \| | 2002/10098 | ELI LILLY AND COMPANY | Wolfgang GLAESNER | |
| Abstract: Disclosed are glucagon-like peptide-1 (GLP-1) comppunds with modifications at one or more of the following posi: 11, 12, 16, 22, 23, 24, 5, 27, 30, 33, 34, 35, 36, or 37. Methods of treating these GLP-1 compounds are also disclosed. | | | | |
| 131. [1417223](https://patentscope.wipo.int/search/en/detail.jsf?docId=EP14109560&recNum=131&office=&queryString=FP%3Aanalog*+AND+PA%3ALilly+AND+PD%3A%28%5B01.01.1994+to+01.01.2015%5D%29+&prevFilter=&sortOption=Pub+Date+Desc&maxRec=494) **PROCESS FOR PREPARING LIPID II** | | | EP | 12.05.2004 |
| \| C07K 1/10 \| Top of Form    Bottom of Form \| \| --- \| --- \| | 02723498 | LILLY CO ELI | BLASZCZAK LARRY CHRIS | |
| A process is described for preparing a substrate for the transglycosylase enzymes of bacterial cell wall biosynthesis. The chemical synthesis makes available a sustainable and substantially pure source of supply of lipid II, including analogs thereof, that may be used in the identification of new therapeutic agents capable of disrupting steps in bacterial cell wall biosynthesis. | | | | |
| 132. [1020040039302](https://patentscope.wipo.int/search/en/detail.jsf?docId=kr596899&recNum=132&office=&queryString=FP%3Aanalog*+AND+PA%3ALilly+AND+PD%3A%28%5B01.01.1994+to+01.01.2015%5D%29+&prevFilter=&sortOption=Pub+Date+Desc&maxRec=494) **GLUCAGON-LIKE PEPTIDE-1 ANALOGS** | | | kr | 10.05.2004 |
| \| C07K 14/605 \| Top of Form    Bottom of Form \| \| --- \| --- \| | 1020047002632 | ELI LILLY AND COMPANY | GLAESNER WOLFGANG | |
| Disclosed are glucagon-like peptide-1 (GLP-1) compounds with modifications at one or more of the following positions: 7, 8, 12, 16, 18, 19, 20, 22, 25, 27, 30, 33, and 37. Methods of treating a subject in need of GLP-1 receptor stimulation using these GLP-1 compounds are also disclosed.  © KIPO & WIPO 2007 | | | | |
| 133. [20040068094](https://patentscope.wipo.int/search/en/detail.jsf?docId=US40400832&recNum=133&office=&queryString=FP%3Aanalog*+AND+PA%3ALilly+AND+PD%3A%28%5B01.01.1994+to+01.01.2015%5D%29+&prevFilter=&sortOption=Pub+Date+Desc&maxRec=494) **Ring modified cyclic peptide analogs** | | | US | 08.04.2004 |
| \| C07K 7/50 \| Top of Form    Bottom of Form \| \| --- \| --- \| | 10676575 | Eli Lilly and Company | Borromeo Peter Stanley | |
| A method for modifying the cyclic peptide ring system of Echinocandin-type compounds to produce new analogs having antifungal activity is provided. The inventive process comprises opening the cyclic peptide ring, cleaving the terminal ornithine unit, inserting at least one new amino acid or other synthetic unit and closing the ring to produce a new cyclic peptide ring structure. The process allows one to incorporate features such as water-solubility into the cyclic peptide ring nucleus, sites for further modification, increase or decrease the number of amino acid or peptide units within the ring nucleus, and increase or decrease the total number of members within the ring. The invention further provides novel Echinocandin type compounds and their use as antifungal or anti-parasitic agents. | | | | |
| 134. [1396272](https://patentscope.wipo.int/search/en/detail.jsf?docId=EP14085968&recNum=134&office=&queryString=FP%3Aanalog*+AND+PA%3ALilly+AND+PD%3A%28%5B01.01.1994+to+01.01.2015%5D%29+&prevFilter=&sortOption=Pub+Date+Desc&maxRec=494) **Insoluble Insulin Compositions for Controlling Blood Glucose** | | | EP | 10.03.2004 |
| \| A61K 38/28 \| Top of Form    Bottom of Form \| \| --- \| --- \| | 03104237 | LILLY CO ELI | BRADER MARK LAURENCE | |
| The present invention relates to insoluble compositions comprising a protein selected from the group consisting of insulin, insulin analogs, and proinsulins; a derivatized protein selected from the group consisting of derivatized insulin, derivatized insulin analog, and derivatized proinsulin; a complexing compound; a hexamer-stabilizing compound; and a divalent metal cation. Formulations of the insoluble composition are suitable for both parenteral and non-parenteral delivery for treating hyperglycemia and diabetes. Microcrystal forms of the insoluble precipitate are pharmaceutically analogous to the neutral protamine Hagedorn (NPH) insulin crystal form. Surprisingly, it has been discovered that suspension formulations of such insoluble compositions possess unique and controllable dissolution properties that provide therapeutically advantageous glucodynamics compared with insulin NPH formulations. | | | | |
| 135. [699686](https://patentscope.wipo.int/search/en/detail.jsf?docId=PT108203434&recNum=135&office=&queryString=FP%3Aanalog*+AND+PA%3ALilly+AND+PD%3A%28%5B01.01.1994+to+01.01.2015%5D%29+&prevFilter=&sortOption=Pub+Date+Desc&maxRec=494) **FRAGMENTOS DE PEPTIDO INSULINOTROPICO DO TIPO GLUCAGONA BIOLOGICAMENTE ACTIVOS** | | | PT | 27.02.2004 |
| \| A61K 38/00 \| Top of Form    Bottom of Form \| \| --- \| --- \| | 95305963 | LILLY CO ELI | JOHNSON WILLIAM TERRY | |
| N-terminal truncated forms of glucagon like insulinotropic peptide (GLP-1) and analogs thereof are provided. The claimed polypeptides promote glucose uptake by cells but do not stimulate insulin expression or secretion. The invention also provides methods for treating diabetes and pharmaceutical formulations comprising the claimed polypeptides. | | | | |
| 136. [000069719798](https://patentscope.wipo.int/search/en/detail.jsf?docId=DE104521033&recNum=136&office=&queryString=FP%3Aanalog*+AND+PA%3ALilly+AND+PD%3A%28%5B01.01.1994+to+01.01.2015%5D%29+&prevFilter=&sortOption=Pub+Date+Desc&maxRec=494) **PERIPHÄRE VERABREICHUNG VON GLP-1 ANALOGEN UND DERIVATE ZUR REGULEIRUNG DER FETTLEIBIGKEIT** | | | DE | 12.02.2004 |
| \| A61K 38/26 \| Top of Form    Bottom of Form \| \| --- \| --- \| | 69719798 | ELI LILLY AND CO., INDIANAPOLIS | DIMARCHI, D. | |
|  | | | | |
| 137. [20040018975](https://patentscope.wipo.int/search/en/detail.jsf?docId=US40473461&recNum=137&office=&queryString=FP%3Aanalog*+AND+PA%3ALilly+AND+PD%3A%28%5B01.01.1994+to+01.01.2015%5D%29+&prevFilter=&sortOption=Pub+Date+Desc&maxRec=494) **Use of GLP-1 analogs and derivatives administered peripherally in regulation of obesity** | | | US | 29.01.2004 |
| \| A61K 38/00 \| Top of Form    Bottom of Form \| \| --- \| --- \| | 10429522 | Eli Lilly and Company | DiMarchi Richard | |
| This invention relates the use of glucagon-like peptides such as GLP-1, a GLP-1 analog, or a GLP-1 derivative in methods and compositions for reducing body weight. | | | | |
| 138. [2002/06360](https://patentscope.wipo.int/search/en/detail.jsf?docId=za1395356&recNum=138&office=&queryString=FP%3Aanalog*+AND+PA%3ALilly+AND+PD%3A%28%5B01.01.1994+to+01.01.2015%5D%29+&prevFilter=&sortOption=Pub+Date+Desc&maxRec=494) **SELECTIVE N-ACYLATION OF A82846 GLYCOPEPTIDE ANALOGS** | | | za | 28.01.2004 |
| \| A61K \| Top of Form    Bottom of Form \| \| --- \| --- \| | 2002/06360 | ELI LILLY AND COMPANY | Richard Craig THOMPSON | |
|  | | | | |
| 139. [2198033](https://patentscope.wipo.int/search/en/detail.jsf?docId=es5570599&recNum=139&office=&queryString=FP%3Aanalog*+AND+PA%3ALilly+AND+PD%3A%28%5B01.01.1994+to+01.01.2015%5D%29+&prevFilter=&sortOption=Pub+Date+Desc&maxRec=494) **COMPUESTOS DE IMIDAZOLINA HIPOGLUCEMICOS.** | | | es | 16.01.2004 |
| \| A61K 31/4178 \| Top of Form    Bottom of Form \| \| --- \| --- \| | E98310461 | ELI LILLY AND COMPANY | JIROUSEK, MICHAEL ROBERT | |
| LA PRESENTE INVENCION SE REFIERE A DETERMINADOS COMPUESTOS NUEVOS DE IMIDAZOLINA Y SUS ANALOGOS, A SU USO PARA EL TRATAMIENTO DE LA DIABETES, LAS COMPLICACIONES DIABETICAS, LOS TRASTORNOS METABOLICOS O ENFERMEDADES RELACIONADAS EN LAS QUE EXISTE UNA ALTERACION EN LA EVACUACION DE LA GLUCOSA, A COMPOSICIONES FARMACEUTICAS QUE LOS COMPRENDEN Y A LOS PROCEDIMIENTOS PARA SU PREPARACION. LOS COMPUESTOS TIENEN LA FORMULA SIGUIENTE: EN LA QUE: X ES -O-, -S-, O -NR5 -, R5 ES HIDROGENO, ALQUILO C1-8 , O UN GRUPO PROTECTOR AMINO; R1, R2 , R3 SON, INDEPENDIENTEMENTE, HIDROGENO O ALQUILO C1-8 ; R 1 Y R 2 FORMAN OPTATIVAMENTE Y EN CONJUNTO UN ENLACE Y R 1'' Y R 3 SON, INDEPENDIENTEMENTE, HIDROGENO O ALQUILO C1-8 ; R1 Y R2 SE COMBINAN O PTATIVAMENTE JUNTOS CON LOS ATOMOS DE CARBONO A LOS QUE VAN ACOPLADOS PARA FORMAR UN ANILLO CARBOCICLICO C3-7 Y R1'' Y R3 SON, INDEPENDIENTEMENTE, HIDROGENO O ALQUILO C1-8 ; R1 Y R1'' , JUNTO CON EL ATOMO DE CARBONO AL QUE VAN ACOPLADOS SE COMBINAN OPTATIVAMENTE PARA FORMARUN ANILLO ESPIROCARBOCICLICO C3-7 , Y R2 Y R3 SON INDEPENDIENTEMENTE, HIDROGENO O ALQUILO C1-8 ; R2 Y R3 , JUNTO CON EL ATOMO DE CARBONO AL QUE VAN ACOPLADOS, SE COMBINAN OPTATIVAMENTE PARA FORMAR UN ANILLO ESPIROCARBOCICLICO C 3-7 Y R 1 Y R 1'' SON, INDEPENDIENTEMENT E, HIDROGENO O ALQUILO C1-8 ; N ES 0, 1 O 2; M ES 0, 1 O 2; M'' ES 0, 1 O 2; Q'' ES 0, 1, 2, 3, 4 O5; R4 ES Y ES -O-, -S-, O -NR6 -; Y'' ES -0- O -S-; R6 Y R7 SON, INDEPENDIENTEMENTE, HIDROGENO, ALQUILO C1-8 , CICLOALQUILO C3-7 , ALCOXI C1-8 , ALQUILTIO C1-8 , ALQUILTIO C1-8 HALO, ALQUILSULFINILO C1-8 , ALQUILSULFONILO C1-8 , CICLOALCOXI C3-7 , ARIL-ALCOXI C1-8 , HALO, HALO- ALQUILO C1-8 , HALO- ALCOXI C18 , NITRO, NR10 R11 , -CON R10 R11 , ARILOALQUILO C1-8 , HETEROCICLILO OPTATIVAMENTE SUSTITUIDO, FENILO OPTATIVAMENTE SUSTITUIDO, NAFTILO OPTATIVAMENTE SUSTITUIDO, ACILAMINO OPTATIVAMENTE HALO-SUSTITUIDO, CIANO, HIDROXI, COR12 , HALO-ALQUILSULFINILO C1-8 , O HALOALQUILSULFONILO C1-8 , O ALCOXIALQUILO DE FORMULA: EN LA QUE P ES 0,1, 2, 3 O 4; Y Q ES 1, 2, 3, 4, O 5; R12 ES ALQUILO C1-8 O FENILO OPTATIVAMENTE SUSTITUIDO; R8 ES HIDROGENO, ALQUILO C1-8 , HALOALQUILO C1-8), FENILO OPTATIVAMENTE SUSTITUIDO, HETEROCICLILO OPTATIVAMENTE SUSTITUIDO, COO-ALQUILO C1-8 , CO-ARILO OPTATIVAMENTE SUSTITUIDO, CO-ALQUILO C1-8 , SO2 C1-8 , ARILO SO2 OPTATIVAMENTE SUSTITUIDO, FENIL-ALQUILO C1-8 OPTATIVAMENTE SUSTITUIDO, CH3 ((CH2) P )-O(CH2) 1 -O-; R9 ES HIDROGENO, HALO, ALQUILO C1-8 ,HALOALQUILTIO C1-8 , ALQU ILTIO C1-8 , ALQUILTIO-HALO C1-8 , CICLOALQUILTIO C3-7 , ARILTIO O HETEROARILTIO OPTATIVAMENTE SUSTITUIDO, ALCOXI C1-8 , CICLOALCOXI C3-7 , ARILOXI OPTATIVAMENTE SUSTITUIDO, HETEROARILOXI OPTATIVAMENTE SUSTITUIDO, O ARILO O HETEROARILO OPTATIVAMENTE SUSTITUIDO, CICLOALQUILO C3-7 , HALO-CICLOALQUILO C3-7 , CICLOALQUENILO C3-7 , CIANO, COOR10 , CONR10 R11 O NR10 R11 , ALQUENILO C2-6 O HETEROCICLILO OPTATIVAMENTE SUSTITUIDO. | | | | |
| 140. [2198033](https://patentscope.wipo.int/search/en/detail.jsf?docId=ES5570599&recNum=140&office=&queryString=FP%3Aanalog*+AND+PA%3ALilly+AND+PD%3A%28%5B01.01.1994+to+01.01.2015%5D%29+&prevFilter=&sortOption=Pub+Date+Desc&maxRec=494) **COMPUESTOS DE IMIDAZOLINA HIPOGLUCEMICOS.** | | | ES | 16.01.2004 |
| \| A61K 31/4178 \| Top of Form    Bottom of Form \| \| --- \| --- \| | E98310461 | ELI LILLY AND COMPANY | JIROUSEK, MICHAEL ROBERT | |
| LA PRESENTE INVENCION SE REFIERE A DETERMINADOS COMPUESTOS NUEVOS DE IMIDAZOLINA Y SUS ANALOGOS, A SU USO PARA EL TRATAMIENTO DE LA DIABETES, LAS COMPLICACIONES DIABETICAS, LOS TRASTORNOS METABOLICOS O ENFERMEDADES RELACIONADAS EN LAS QUE EXISTE UNA ALTERACION EN LA EVACUACION DE LA GLUCOSA, A COMPOSICIONES FARMACEUTICAS QUE LOS COMPRENDEN Y A LOS PROCEDIMIENTOS PARA SU PREPARACION. LOS COMPUESTOS TIENEN LA FORMULA SIGUIENTE: EN LA QUE: X ES -O-, -S-, O -NR5 -, R5 ES HIDROGENO, ALQUILO C1-8 , O UN GRUPO PROTECTOR AMINO; R1, R2 , R3 SON, INDEPENDIENTEMENTE, HIDROGENO O ALQUILO C1-8 ; R 1 Y R 2 FORMAN OPTATIVAMENTE Y EN CONJUNTO UN ENLACE Y R 1'' Y R 3 SON, INDEPENDIENTEMENTE, HIDROGENO O ALQUILO C1-8 ; R1 Y R2 SE COMBINAN O PTATIVAMENTE JUNTOS CON LOS ATOMOS DE CARBONO A LOS QUE VAN ACOPLADOS PARA FORMAR UN ANILLO CARBOCICLICO C3-7 Y R1'' Y R3 SON, INDEPENDIENTEMENTE, HIDROGENO O ALQUILO C1-8 ; R1 Y R1'' , JUNTO CON EL ATOMO DE CARBONO AL QUE VAN ACOPLADOS SE COMBINAN OPTATIVAMENTE PARA FORMARUN ANILLO ESPIROCARBOCICLICO C3-7 , Y R2 Y R3 SON INDEPENDIENTEMENTE, HIDROGENO O ALQUILO C1-8 ; R2 Y R3 , JUNTO CON EL ATOMO DE CARBONO AL QUE VAN ACOPLADOS, SE COMBINAN OPTATIVAMENTE PARA FORMAR UN ANILLO ESPIROCARBOCICLICO C 3-7 Y R 1 Y R 1'' SON, INDEPENDIENTEMENT E, HIDROGENO O ALQUILO C1-8 ; N ES 0, 1 O 2; M ES 0, 1 O 2; M'' ES 0, 1 O 2; Q'' ES 0, 1, 2, 3, 4 O5; R4 ES Y ES -O-, -S-, O -NR6 -; Y'' ES -0- O -S-; R6 Y R7 SON, INDEPENDIENTEMENTE, HIDROGENO, ALQUILO C1-8 , CICLOALQUILO C3-7 , ALCOXI C1-8 , ALQUILTIO C1-8 , ALQUILTIO C1-8 HALO, ALQUILSULFINILO C1-8 , ALQUILSULFONILO C1-8 , CICLOALCOXI C3-7 , ARIL-ALCOXI C1-8 , HALO, HALO- ALQUILO C1-8 , HALO- ALCOXI C18 , NITRO, NR10 R11 , -CON R10 R11 , ARILOALQUILO C1-8 , HETEROCICLILO OPTATIVAMENTE SUSTITUIDO, FENILO OPTATIVAMENTE SUSTITUIDO, NAFTILO OPTATIVAMENTE SUSTITUIDO, ACILAMINO OPTATIVAMENTE HALO-SUSTITUIDO, CIANO, HIDROXI, COR12 , HALO-ALQUILSULFINILO C1-8 , O HALOALQUILSULFONILO C1-8 , O ALCOXIALQUILO DE FORMULA: EN LA QUE P ES 0,1, 2, 3 O 4; Y Q ES 1, 2, 3, 4, O 5; R12 ES ALQUILO C1-8 O FENILO OPTATIVAMENTE SUSTITUIDO; R8 ES HIDROGENO, ALQUILO C1-8 , HALOALQUILO C1-8), FENILO OPTATIVAMENTE SUSTITUIDO, HETEROCICLILO OPTATIVAMENTE SUSTITUIDO, COO-ALQUILO C1-8 , CO-ARILO OPTATIVAMENTE SUSTITUIDO, CO-ALQUILO C1-8 , SO2 C1-8 , ARILO SO2 OPTATIVAMENTE SUSTITUIDO, FENIL-ALQUILO C1-8 OPTATIVAMENTE SUSTITUIDO, CH3 ((CH2) P )-O(CH2) 1 -O-; R9 ES HIDROGENO, HALO, ALQUILO C1-8 ,HALOALQUILTIO C1-8 , ALQU ILTIO C1-8 , ALQUILTIO-HALO C1-8 , CICLOALQUILTIO C3-7 , ARILTIO O HETEROARILTIO OPTATIVAMENTE SUSTITUIDO, ALCOXI C1-8 , CICLOALCOXI C3-7 , ARILOXI OPTATIVAMENTE SUSTITUIDO, HETEROARILOXI OPTATIVAMENTE SUSTITUIDO, O ARILO O HETEROARILO OPTATIVAMENTE SUSTITUIDO, CICLOALQUILO C3-7 , HALO-CICLOALQUILO C3-7 , CICLOALQUENILO C3-7 , CIANO, COOR10 , CONR10 R11 O NR10 R11 , ALQUENILO C2-6 O HETEROCICLILO OPTATIVAMENTE SUSTITUIDO. | | | | |
| 141. [1468258](https://patentscope.wipo.int/search/en/detail.jsf?docId=CN82659489&recNum=141&office=&queryString=FP%3Aanalog*+AND+PA%3ALilly+AND+PD%3A%28%5B01.01.1994+to+01.01.2015%5D%29+&prevFilter=&sortOption=Pub+Date+Desc&maxRec=494) **Glucagon-like peptide-1 analogs** | | | CN | 14.01.2004 |
| \| C07K 14/605 \| Top of Form    Bottom of Form \| \| --- \| --- \| | 01811213.7 | Eli Lilly and Co. | Glaesner Wolfgang | |
| Disclosed are glucagon-like peptide-1 (GLP-1) compounds with modifications at one or more of the following positions: 11, 12, 16, 22, 23, 24, 25, 27, 30, 33, 34, 35, 36, or 37. Methods of treating a subject in need of GLP-1 receptor stimulation using these GLP-1 compounds are also disclosed. | | | | |
| 142. [WO/2003/103572](https://patentscope.wipo.int/search/en/detail.jsf?docId=WO2003103572&recNum=142&office=&queryString=FP%3Aanalog*+AND+PA%3ALilly+AND+PD%3A%28%5B01.01.1994+to+01.01.2015%5D%29+&prevFilter=&sortOption=Pub+Date+Desc&maxRec=494) **MODIFIED GLUCAGON-LIKE PEPTIDE-1 ANALOGS** | | | WO | 18.12.2003 |
| \| [C07K 7/00](http://www.wipo.int/ipcpub/?symbol=C07K0007000000&refresh=page&viewmode=a&notes=no&headings=no&showdeleted=no) \| Top of Form    Bottom of Form \| \| --- \| --- \| | PCT/US2003/015395 | ELI LILLY AND COMPANY | DIMARCHI, Richard, Dennis | |
| The invention encompasses GLP-1 compounds containing a GLP-1 peptide or a GLP-1 peptide with an extended C-terminus that is modified with a reactive group that is capable of forming covalent bonds with a blood component to form a conjugate. The conjugates may be formed in vivo or ex vivo. Methods of treating a subject in need of GLP-1 receptor stimulation using these GLP-1 compounds are also disclosed. | | | | |
| 143. [2194890](https://patentscope.wipo.int/search/en/detail.jsf?docId=es5478060&recNum=143&office=&queryString=FP%3Aanalog*+AND+PA%3ALilly+AND+PD%3A%28%5B01.01.1994+to+01.01.2015%5D%29+&prevFilter=&sortOption=Pub+Date+Desc&maxRec=494) **ANALOGOS DE INSULINA ACILADOS.** | | | es | 01.12.2003 |
| \| A61K 38/28 \| Top of Form    Bottom of Form \| \| --- \| --- \| | E95308166 | ELI LILLY AND COMPANY | BAKER, JEFFREY CLAYTON | |
| LA INVENCION SE REFIERE AL CAMPO DE LA DIABETES. MAS PARTICULARMENTE, LA INVENCION SE REFIERE A UN ANALOGO MONOMERICO DE LA INSULINA EN EL QUE LA CADENA A ES LA SECUENCIA QUE SE PRODUCE NATURALMENTE DE LA CADENA A DE LA INSULINA HUMANA Y LA CADENA B ESTA MODIFICADA EN CUALQUIER POSICION B28 O B29 O EN AMBAS. EL ANALOGO ESTA MONOACILADO EN EL TERMINAL N DE LA CADENA A O DE LA CADENA B O EN LA LISINA. LOS ANALOGOS ACILADOS DE LA INSULINA TIENEN UN PERIODO DE ACCION DE DURACION EXTENDIDA. | | | | |
| 144. [2194890](https://patentscope.wipo.int/search/en/detail.jsf?docId=ES5478060&recNum=144&office=&queryString=FP%3Aanalog*+AND+PA%3ALilly+AND+PD%3A%28%5B01.01.1994+to+01.01.2015%5D%29+&prevFilter=&sortOption=Pub+Date+Desc&maxRec=494) **ANALOGOS DE INSULINA ACILADOS.** | | | ES | 01.12.2003 |
| \| A61K 38/28 \| Top of Form    Bottom of Form \| \| --- \| --- \| | E95308166 | ELI LILLY AND COMPANY | BAKER, JEFFREY CLAYTON | |
| LA INVENCION SE REFIERE AL CAMPO DE LA DIABETES. MAS PARTICULARMENTE, LA INVENCION SE REFIERE A UN ANALOGO MONOMERICO DE LA INSULINA EN EL QUE LA CADENA A ES LA SECUENCIA QUE SE PRODUCE NATURALMENTE DE LA CADENA A DE LA INSULINA HUMANA Y LA CADENA B ESTA MODIFICADA EN CUALQUIER POSICION B28 O B29 O EN AMBAS. EL ANALOGO ESTA MONOACILADO EN EL TERMINAL N DE LA CADENA A O DE LA CADENA B O EN LA LISINA. LOS ANALOGOS ACILADOS DE LA INSULINA TIENEN UN PERIODO DE ACCION DE DURACION EXTENDIDA. | | | | |
| 145. [20030220243](https://patentscope.wipo.int/search/en/detail.jsf?docId=US40070848&recNum=145&office=&queryString=FP%3Aanalog*+AND+PA%3ALilly+AND+PD%3A%28%5B01.01.1994+to+01.01.2015%5D%29+&prevFilter=&sortOption=Pub+Date+Desc&maxRec=494) **Glucagon-like peptide-1 analogs** | | | US | 27.11.2003 |
| \| A61K 38/00 \| Top of Form    Bottom of Form \| \| --- \| --- \| | 10276772 | Eli Lilly and Company | Glaesner Wolfgang | |
| Disclosed are glucagon-like peptide-1 (GLP-1) compounds with modifications at one or more of the following positions: 11, 12, 16, 22, 23, 24, 25, 27, 30, 33, 34, 35, 36, or 37. Methods of treating these GLP-1 compounds are also disclosed. | | | | |
| 146. [PA/a/2002/012203](https://patentscope.wipo.int/search/en/detail.jsf?docId=mx106161&recNum=146&office=&queryString=FP%3Aanalog*+AND+PA%3ALilly+AND+PD%3A%28%5B01.01.1994+to+01.01.2015%5D%29+&prevFilter=&sortOption=Pub+Date+Desc&maxRec=494) **GLUCAGON-LIKE PEPTIDE-1 ANALOGS** | | | mx | 17.11.2003 |
| \| C07K 14/00 \| Top of Form    Bottom of Form \| \| --- \| --- \| | PA/a/2002/012203 | ELI LILLY AND COMPANY | GLAESNER, Wolfgang | |
| Disclosed are glucagon-like peptide-1 (GLP-1) compounds with modifications at one or more of the following positions:11, 12, 16, 22, 23, 24, 25, 27, 30, 33, 34, 35, 36, or 37. Methods of treating these GLP-1 compounds are also disclosed. | | | | |
| 147. [2194224](https://patentscope.wipo.int/search/en/detail.jsf?docId=es5675285&recNum=147&office=&queryString=FP%3Aanalog*+AND+PA%3ALilly+AND+PD%3A%28%5B01.01.1994+to+01.01.2015%5D%29+&prevFilter=&sortOption=Pub+Date+Desc&maxRec=494) **USO DE ANALOGOS Y DERIVADOS DE GLP-1 ADMINISTRADOS PERIFERICAMENTE PARA LA REGULACION DE LA OBESIDAD.** | | | es | 16.11.2003 |
| \| C07K 14/605 \| Top of Form    Bottom of Form \| \| --- \| --- \| | E97947357 | ELI LILLY AND COMPANY | DIMARCHI, RICHARD, D. | |
| ESTA INVENCION SE REFIERE AL USO DE PEPTIDOS DEL TIPO GLUCAGON COMO GLP - 1, UN ANALOGO DE ESTE ULTIMO O UN DERIVADO DE GLP 1, EN UNAS COMPOSICIONES PARA REDUCIR LA SOBRECARGA PONDERAL ASI COMO EN EL MARCO DE LOS PROCEDIMIENTOS CORRESPONDIENTES. | | | | |
| 148. [2194224](https://patentscope.wipo.int/search/en/detail.jsf?docId=ES5675285&recNum=148&office=&queryString=FP%3Aanalog*+AND+PA%3ALilly+AND+PD%3A%28%5B01.01.1994+to+01.01.2015%5D%29+&prevFilter=&sortOption=Pub+Date+Desc&maxRec=494) **USO DE ANALOGOS Y DERIVADOS DE GLP-1 ADMINISTRADOS PERIFERICAMENTE PARA LA REGULACION DE LA OBESIDAD.** | | | ES | 16.11.2003 |
| \| C07K 14/605 \| Top of Form    Bottom of Form \| \| --- \| --- \| | E97947357 | ELI LILLY AND COMPANY | DIMARCHI, RICHARD, D. | |
| ESTA INVENCION SE REFIERE AL USO DE PEPTIDOS DEL TIPO GLUCAGON COMO GLP - 1, UN ANALOGO DE ESTE ULTIMO O UN DERIVADO DE GLP 1, EN UNAS COMPOSICIONES PARA REDUCIR LA SOBRECARGA PONDERAL ASI COMO EN EL MARCO DE LOS PROCEDIMIENTOS CORRESPONDIENTES. | | | | |
| 149. [1354045](https://patentscope.wipo.int/search/en/detail.jsf?docId=EP14009732&recNum=149&office=&queryString=FP%3Aanalog*+AND+PA%3ALilly+AND+PD%3A%28%5B01.01.1994+to+01.01.2015%5D%29+&prevFilter=&sortOption=Pub+Date+Desc&maxRec=494) **HYPERGLYCOSYLATED POLYPEPTIDES** | | | EP | 22.10.2003 |
| \| A61K 38/19 \| Top of Form    Bottom of Form \| \| --- \| --- \| | 01967940 | LILLY CO ELI | BEALS JOHN MICHAEL | |
| The present invention addresses the need for better pharmaceutical agents for treating patients that have reduced circulating levels of neutrophilic granulocytes, such as after chemotherpay regimens or in chronic congenital neutropneia by providing novel biological active glycosylated G-CSF analogs. | | | | |
| 150. [1351984](https://patentscope.wipo.int/search/en/detail.jsf?docId=EP13992600&recNum=150&office=&queryString=FP%3Aanalog*+AND+PA%3ALilly+AND+PD%3A%28%5B01.01.1994+to+01.01.2015%5D%29+&prevFilter=&sortOption=Pub+Date+Desc&maxRec=494) **AMIDATED GLUCAGON-LIKE PEPTIDE-1** | | | EP | 15.10.2003 |
| \| A61K 38/26 \| Top of Form    Bottom of Form \| \| --- \| --- \| | 01989720 | LILLY CO ELI | MILLICAN ROHN LEE JUNIOR | |
| The present invention encompasses a GLP-1 analog and compositions and formulations thereof useful for the treatment of hyperglycemia and other various diseases and conditions in mammals. | | | | |
| 151. [2003-0820](https://patentscope.wipo.int/search/en/detail.jsf?docId=pe21168441&recNum=151&office=&queryString=FP%3Aanalog*+AND+PA%3ALilly+AND+PD%3A%28%5B01.01.1994+to+01.01.2015%5D%29+&prevFilter=&sortOption=Pub+Date+Desc&maxRec=494) **ANALOGOS EXTENDIDOS DE PEPTIDO 1 DE TIPO GLUCAGON** | | | pe | 04.10.2003 |
| \| G01N 1/00 \| Top of Form    Bottom of Form \| \| --- \| --- \| | 2003000021 | ELI LILLY AND COMPANY | WOLFGANG GLAESNER | |
| SE REFIERE A UN PEPTIDO 1 DE TIPO GLUCAGON GLP-1 EXTENDIDO QUE COMPRENDE UNA SECUENCIA DE AMINOACIDOS 1; DONDE Xaa7 ES HISTIDINA, DESAMINOHISTIDINA, 2-AMNOHISTIDINA, ß-HIDROXIHISTIDINA, HOMOHISTIDINA, O-FLUOROMETILHISTIDINA, O-METILHISTIDINA; Xaa8 ES ALA, GLY, VAL, LEU, ILE, SER, THR; Xaa12 ES PHE, TRP, TYR, Xaa16 ES VAL, TRP, LEU, PHE, TYR; Xaa18 ES SER, TRP, TYR, PHE, LYS, ILE, LEU, VAL; Xaa19 ES TYR, TRP, PHE; Xaa20 ES LEU, PHE, TYR, TRP; Xaa22 ES GLY, GLU, ASP, LYS; Xaa25 ES ALA, VAL, ILE, LEU; Xaa27 ES GLU, ILE, ALA; Xaa30 ES ALA, GLU, Xaa33 ES VAL, ILE; Xaa34 ES LYS, ASP, ARG, GLU; Xaa36 ES GLY, PRO, ARG; Xaa37 ES GLY, PRO, SER; Xaa38 ES SER, PRO, HIS; Xaa39 ES SER, ARG, THR, TRP, LYS; Xaa 40 ES SER, GLY; Xaa41 ES ALA, ASP, ARG, GLU, LYS, GLY, Xaa42 ES PRO, ALA, NH2; Xaa43 ES PRO, ALA, NH2; Xaa44 ES PRO, ALA, ARG, LYS, HIS, NH2, XAA45 ES SER, HIS, PRO, LYS, ARG, NH2, Xaa46 ES HIS, SER, ARG, NH2; Xaa47 ES HIS, SER, ARG, LYS, NH2; SI Xaa42-Xaa47 ESTAN AUSENTES CADA AMINOACIDO CADENA ABAJO ESTA AUSENTE, ENTRE OTRAS CONDICIONES; CARACTERIZADO PORQUE LOS PRIMEROS 31 AMINOACIDOS DEL PEPTIDO NO DIFIEREN DE GLP-1(7-37) EN MAS DE 6 AMINOACIDOS. EL PEPTIDO GLP-1 AL QUE SE HAN ANADIDO AMINOACIDOS EN C-TERMINAL PRESENTA SEMIVIDA SERICA MAS LARGA Y ES ADECUADO PARA ADMINISTRACION ORAL Y PULMONAR DEBIDO A QUE ES RESISTENTE A ENZIMAS PROTEOLITICAS Y PUEDE SER UTIL PARA LA ESTIMULACION DEL RECEPTOR DE GLP-1 EN UN SUJETO NECESITADO DE NORMALIZACION DE GLUCOSA EN SANGRE COMO DIABETES N | | | | |
| 152. [031701](https://patentscope.wipo.int/search/en/detail.jsf?docId=ar5300640&recNum=152&office=&queryString=FP%3Aanalog*+AND+PA%3ALilly+AND+PD%3A%28%5B01.01.1994+to+01.01.2015%5D%29+&prevFilter=&sortOption=Pub+Date+Desc&maxRec=494) **COMPUESTOS ANÁLOGOS DEL (GLP-1)-PEPTIDO-1 ANALOGO DEL GLUCAGÓN, METODO PARA ESTIMULAR LOS RECEPTORES GLP-1 UTILIZANDO DICHOS COMPUESTOS Y USO DE DICHOS COMPUESTOS EN LA PREPARACION DE MEDICAMENTOS PARA ESTIMULAR LA SECRECION DE INSULINA** | | | ar | 01.10.2003 |
| \| A61K 38/26 \| Top of Form    Bottom of Form \| \| --- \| --- \| | P010102775 | ELI LILLY AND COMPANY |  | |
| Se describen y reivindican análogos al glucagón (GLP-1), diferenciados por al menos una sustitución en las siguientes posiciones: 11, 12, 16, 22, 23, 24, 25, 27, 30, 33, 34, 35, 36 o 37 y además un procedimiento para estimular dichos receptores GLP-1 utilizando dichos análogos y el uso de los mismos para preparar medicamentos para el tratamiento de diabetes no dependientes de la insulina, obesidad, apoplejía, infarto de miocardio, cambios catabólicos post quirúrgicos y síndromes del intestino irritable. En general, dichos análogos poseen manifiesta una reducida capacidad para formar agregados con respecto al GLP-1 (7-37)OH, siendo su capacidad de activación de los receptores GLP-1, al menos comparable con otros compuestos GLP-1. Compuestos análogos del GLP-1 caracterizado porque comprende la secuencia de aminoácidos de fórmula 1(SEC ID NO: 1): His-Xaa8-Glu-Gly-Xaa11-Xaa12-Thr-Ser-Asp-Xaa16-Ser- Ser-Tyr-Leu-Glu-Xaa22-Xaa23-Xaa24-Ala-Xaa26-Xaa27-Phe-Ile-Xaa30-Trp-Leu-Xaa33-Xaa34-Xaa35-Xaa36-R. Fórmula 1 (SEC ID NO:1) en la que: Xaa8 es: Gly, Ala, Val, Leu, Ile, Ser o Thr; Xaa11 es: Asp, Glu, Arg, Thr, Ala, Lys o His; Xaa12 es: His, Trp, Phe o Tyr; Xaa16 es: Leu, Ser, Thr, Trp, His, Phe, Asp, Val, Tyr, Glu o Ala; Xaa22 es: Gly, Asp, Glu, Gln, Asn, Lys, Arg, Cys o Ácido Cisteico; Xaa23 es: His, Asp, Lys, Glu, Gln o Arg; Xaa24 es Glu, Arg, Ala o Lys; Xaa26 es: Trp, Tyr, Phe, Asp, Lys, Glu oHis; Xaa27 es Ala, Glu, His, Phe, Tyr, Trp, Arg o Lys; Xaa30 es: Ala, Glu, Asp, Ser o His; Xaa33 es: Asp, Arg, Val, Lys, Ala, Gly o Glu; Xaa34 es: Glu, Lys o Asp; Xaa35 es Thr, Ser, Lys, Arg, Trp, TYr, Phe, Asp, Gly, Pro, His o Glu; Xaa36 es: thR, Ser, Asp, Tyr, Phe, Arg, Glu o His; y R es: Lys, Arg, Thr, Ser, Glu, Asp, Tro, Tyr, Phe, His, NH2, Gly, Gly-Pro-NH2, o está delecionado, con la condición de que el compuesto GLP-1 no tenga la secuencia de GLP-1(7-37)OH o GLP-1(7-36)-NH2 y con la condición de que el compiuesto GLP-1 no sea Gly8-GLP-1-(7-37)OH, Gly8-GLP-1(7-36)NH2, Val8-GLP-1(7-37)OH, Val8-GLP-1(7-36)NH2, Leu8-GLP-1(7-37)OH, Leu8-GLP-1(7-36)NH2, Ile8-GLP-1-(7-37)OH, Ile8-GLP-1(7-36)NH2, Ser8-GLP-1(7-37)OH, Ser8-GLP-1-(7-36)NH2, Thr8-GLP-1-(7-37)OH o Thr8-GLP-1(7-36)NH2, Ala11-GLP-1-(7-37)OH, Ala11-GLP-1(7-36)NH2, Ala16-GLP-1(7-37)OH, Ala16-GLP-1(7-36)NH2, Ala18-GLP-1(7-37)OH, Ala18-GLP-1(7-36)NH2, Ala27-GLP-1 (7-37)OH, Ala27-GLP-1(7-36)NH2, Ala33-GLP-1(7-37)OH o Ala33-GLP-1-(7-36)NH2. | | | | |
| 153. [WO/2003/076567](https://patentscope.wipo.int/search/en/detail.jsf?docId=WO2003076567&recNum=153&office=&queryString=FP%3Aanalog*+AND+PA%3ALilly+AND+PD%3A%28%5B01.01.1994+to+01.01.2015%5D%29+&prevFilter=&sortOption=Pub+Date+Desc&maxRec=494) **HETEROLOGOUS G-CSF FUSION PROTEINS** | | | WO | 18.09.2003 |
| \| [A61K 39/00](http://www.wipo.int/ipcpub/?symbol=A61K0039000000&refresh=page&viewmode=a&notes=no&headings=no&showdeleted=no) \| Top of Form    Bottom of Form \| \| --- \| --- \| | PCT/US2003/003120 | ELI LILLY AND COMPANY | BEALS, John, Michael | |
| The present invention encompasses heterologous fusion proteins comprising a hyperglycsoylated G-CSF analog fusedto proteins such as albumin and the Fc portion of animmunoglobulin which act to extend the in vivo half-life ofthe protein compared to native G-CSF. These fusion proteinsare particularly suited for the treatment of conditions treatable by stimulation of circulating neutrophils, such as after chemotherapy regimens or in chronic congenitalneutropenia. | | | | |
| 154. [WO/2003/073982](https://patentscope.wipo.int/search/en/detail.jsf?docId=WO2003073982&recNum=154&office=&queryString=FP%3Aanalog*+AND+PA%3ALilly+AND+PD%3A%28%5B01.01.1994+to+01.01.2015%5D%29+&prevFilter=&sortOption=Pub+Date+Desc&maxRec=494) **ANTI-INTERLEUKIN-1 BETA ANALOGS** | | | WO | 12.09.2003 |
| \| [C07K 16/24](http://www.wipo.int/ipcpub/?symbol=C07K0016240000&refresh=page&viewmode=a&notes=no&headings=no&showdeleted=no) \| Top of Form    Bottom of Form \| \| --- \| --- \| | PCT/US2003/003117 | ELI LILLY AND COMPANY | BEALS, John, Michael | |
| The present invention encompasses analogs of humanized antibody Hu007 that neutralize IL-1β activity in vivo. These antibodies can be used to treat various diseases such as rheumatoid arthritis and osteoarthritis. | | | | |
| 155. [924209](https://patentscope.wipo.int/search/en/detail.jsf?docId=PT108331493&recNum=155&office=&queryString=FP%3Aanalog*+AND+PA%3ALilly+AND+PD%3A%28%5B01.01.1994+to+01.01.2015%5D%29+&prevFilter=&sortOption=Pub+Date+Desc&maxRec=494) **COMPOSTOS DE IMIDAZOLINA HIPOGLICEMICOS** | | | PT | 29.08.2003 |
| \| A61K 31/4164 \| Top of Form    Bottom of Form \| \| --- \| --- \| | 98310461 | LILLY CO ELI | RUTHER GERD | |
| This invention relates to certain novel imidazoline compounds and analogues thereof, to their use for the treatment of diabetes, diabetic complications, metabolic disorders, or related diseases where impaired glucose disposal is present, to pharmaceutical compositions comprising them, and to processes for their preparation. The compounds have the following formula: wherein X is -O-, -S-, or -NR<5>-, R<5> is hydrogen, C1-8 alkyl, or an amino protecting group; R<1>, R<1'>, R<2>, and R<3> are independently hydrogen or C1-8 alkyl; R<1> and R<2> optionally together form a bond and R<1'> and R<3> are independently hydrogen or C1-8 alkyl; R<1> and R<2> optionally combine together with the carbon atoms to which they are attached form a C3-7 carbocyclic ring and R<1'> and R<3> are independently hydrogen or C1-8 alkyl; R<1> and R<1'> together with the carbon atom to which they are attached optionally combine to form a C3-7 spirocarbocyclic ring and R<2> and R<3> are independently hydrogen or C1-8 alkyl; R<2> and R<3> together with the carbon atom to which they are attached optionally combine to form a C3-7 spirocarbocyclic and R<1> and R<1'> are independently hydrogen or C1-8 alkyl; n is 0, 1, or 2; m is 0, 1 or 2; m' is 0, 1, or 2; q' is 0,1,2,3,4, or 5; R<4> is Y is -O-, -S-, or -NR<8>-; Y' is -O- or -S-; R<6> and R<7> are independently hydrogen, C1-8 alkyl, C3-7 cycloalkyl, C1-8 alkoxy, C1-8 alkylthio, halo C1-8 alkylthio, C1-8 alkylsulfinyl, C1-8 alkylsulfonyl, C3-7 cycloalkoxy, aryl-C1-8 alkoxy, halo, halo-C1-8 alkyl, halo- C1-8 alkoxy, nitro, -NR<10>R<11>, -CONR<10>R<11>, aryl C1-8 alkyl, optionally substituted heterocyclyl, optionally substituted phenyl, optionally substituted naphthyl, optionally halo substituted acylamino, cyano, hydroxy, COR<12>, halo C1-8 alkylsulfinyl, or halo C1-8 alkylsulfonyl, or alkoxyalkyl of the formula CH3(CH2)p-O-(CH2)q-O-; where p is 0, 1, 2, 3, or 4; and q is 1, 2, 3, 4, or 5; R<12> is C1-8 alkyl or optionally substituted phenyl; R<8> is hydrogen, C1-8 alkyl, halo-C1_8 alkyl, optionally substituted phenyl, optionally substituted heterocyclyl, COO C1-8 alkyl, optionally substituted COaryl, COC1-8 alkyl, SO2C1-8 alkyl, optionally substituted SO2 aryl, optionally substituted phenyl-C1-8 alkyl, CH3(CH2)p-O-(CH2)q-O-; R<9> is hydrogen, halo, C1-8 alkyl, halo C1-8 alkyl, C1-8 alkylthio, halo C1-8 alkylthio, C3-7 cycloalkylthio, optionally substituted arylthio or heteroarylthio, C1-8 alkoxy, C3-7 cycloalkoxy, optionally substituted aryloxy, optionally substituted heteroaryloxy, or optionally substituted aryl or heteroaryl, C3-7 cycloalkyl, halo C3-7 cycloalkyl, C3-7 cycloalkenyl, cyano, COOR<10>,CONR<10>R<11> or NR<10>R<11>, C2-6 alkenyl, optionally substituted heterocyclyl, optionally substituted aryl C1-8 alkyl, optionally substituted heteroaryl C1-8 alkyl in which the alkyl group can be substituted by hydroxy, or C1-8 alkyl substituted by hydroxy, R<10> and R<11> are independently hydrogen, C1-8 alkyl, optionally substituted aryl C1-8 alkyl, optionally substituted phenyl, or R<10> and R<11> together with the nitrogen atom to which they are attached may combine to form a ring with up to six carbon atoms which optionally may be substituted with up to two C1-8 alkyl groups or one carbon atom may be replaced by oxygen or sulfur; R<14> and R<16> are independently hydrogen, halo, C1-8 alkyl, C3-7 cycloalkyl, C3-7 cycloalkoxy, C3-7 cycloalkylC1-8 alkoxy, halo-C1-8 alkyl, halo-C1-8 alkoxy, C1-8 alkoxy, carbo(C1-8)alkoxy, optionally substituted aryl, or optionally substituted heteroaryl; R<15> and R<17> are independently hydrogen, halo, C1-8 alkoxy, C3-7-cycloalkyl, C3-7 cycloalkylC1-8 alkoxy, C1-8 alkyl, C3-7 cycloalkoxy, hydroxy, halo C1-8 alkoxy, carbo(C1-8)alkoxy, optionally substituted phenyl, optionally substituted phenyl-C1-8 alkyl, optionally substituted phenyloxy, optionally substituted phenyl-C1-8 alkoxy, (tetrahydropyran-2-yl)methoxy, C1-8 alkyl | | | | |
| 156. [WO/2003/058203](https://patentscope.wipo.int/search/en/detail.jsf?docId=WO2003058203&recNum=156&office=&queryString=FP%3Aanalog*+AND+PA%3ALilly+AND+PD%3A%28%5B01.01.1994+to+01.01.2015%5D%29+&prevFilter=&sortOption=Pub+Date+Desc&maxRec=494) **EXTENDED GLUCAGON-LIKE PEPTIDE-1 ANALOGS** | | | WO | 17.07.2003 |
| \| [A61K 38/26](http://www.wipo.int/ipcpub/?symbol=A61K0038260000&refresh=page&viewmode=a&notes=no&headings=no&showdeleted=no) \| Top of Form    Bottom of Form \| \| --- \| --- \| | PCT/US2003/000001 | ELI LILLY AND COMPANY | GLAESNER, Wolfgang | |
| The invention encompasses GLP-1 peptides with modifications at various positions coupled with an extended C-terminus that provides increased stability. | | | | |
| 157. [2468700](https://patentscope.wipo.int/search/en/detail.jsf?docId=CA94114725&recNum=157&office=&queryString=FP%3Aanalog*+AND+PA%3ALilly+AND+PD%3A%28%5B01.01.1994+to+01.01.2015%5D%29+&prevFilter=&sortOption=Pub+Date+Desc&maxRec=494) **EXTENDED GLUCAGON-LIKE PEPTIDE-1 ANALOGS** | | | CA | 17.07.2003 |
| \| C07K 14/605 \| Top of Form    Bottom of Form \| \| --- \| --- \| | 2468700 | ELI LILLY AND COMPANY | GLAESNER, WOLFGANG | |
| The invention encompasses GLP-1 peptides with modifications at various positions coupled with an extended C-terminus that provides increased stability. | | | | |
| 158. [WO/2003/053460](https://patentscope.wipo.int/search/en/detail.jsf?docId=WO2003053460&recNum=158&office=&queryString=FP%3Aanalog*+AND+PA%3ALilly+AND+PD%3A%28%5B01.01.1994+to+01.01.2015%5D%29+&prevFilter=&sortOption=Pub+Date+Desc&maxRec=494) **CRYSTALLINE COMPOSITIONS FOR CONTROLLING BLOOD GLUCOSE** | | | WO | 03.07.2003 |
| \| [A61K 38/28](http://www.wipo.int/ipcpub/?symbol=A61K0038280000&refresh=page&viewmode=a&notes=no&headings=no&showdeleted=no) \| Top of Form    Bottom of Form \| \| --- \| --- \| | PCT/US2002/037602 | ELI LILLY AND COMPANY | BRADER, Mark, Laurence | |
| The present invention relates to insulin crystals formed from zinc, protamine, a hexamer-stabilizing compound, and a polypeptide selected from the group consisting of insulin, an insulin analog, and a derivatized insulin. The crystals are suitable for administering to a patient for control of blood glucose levels. The crystals have been derived from the neutral protamine Hagedorn (NPH) form in a process utilizing precisely determined protamine concentrations and fortification of NPH crystals formed at a first lower concentration of protamine to achieve a second higher concentration of protamine. | | | | |
| 159. [2188637](https://patentscope.wipo.int/search/en/detail.jsf?docId=es5582812&recNum=159&office=&queryString=FP%3Aanalog*+AND+PA%3ALilly+AND+PD%3A%28%5B01.01.1994+to+01.01.2015%5D%29+&prevFilter=&sortOption=Pub+Date+Desc&maxRec=494) **PREPARACION DE CRISTALES ESTABLES DE ZINC ANALOGOS A LA INSULINA.** | | | es | 01.07.2003 |
| \| A61P 5/00 \| Top of Form    Bottom of Form \| \| --- \| --- \| | E95304098 | ELI LILLY AND COMPANY | BAKER, JEFFREY CLAYTON | |
| EN LA PRESENTE INVENCION SE PRESENTA UN PROCESO PARA LA PREPARACION DE UN ANALOGO DE INSULINA CRISTALINO. EL PROCESO ES UTIL PARA LA PURIFICACION Y FABRICACION DE INSULINA HUMANA LYS{SUP,B28}PRO{SUP,B29}. LA INSULINA HUMANA LYS{SUP,B28}PRO{SUP,B29} ES UTIL PARA EL TRATAMIENTO DE LA DIABETES. | | | | |
| 160. [2188637](https://patentscope.wipo.int/search/en/detail.jsf?docId=ES5582812&recNum=160&office=&queryString=FP%3Aanalog*+AND+PA%3ALilly+AND+PD%3A%28%5B01.01.1994+to+01.01.2015%5D%29+&prevFilter=&sortOption=Pub+Date+Desc&maxRec=494) **PREPARACION DE CRISTALES ESTABLES DE ZINC ANALOGOS A LA INSULINA.** | | | ES | 01.07.2003 |
| \| A61P 5/00 \| Top of Form    Bottom of Form \| \| --- \| --- \| | E95304098 | ELI LILLY AND COMPANY | BAKER, JEFFREY CLAYTON | |
| EN LA PRESENTE INVENCION SE PRESENTA UN PROCESO PARA LA PREPARACION DE UN ANALOGO DE INSULINA CRISTALINO. EL PROCESO ES UTIL PARA LA PURIFICACION Y FABRICACION DE INSULINA HUMANA LYS{SUP,B28}PRO{SUP,B29}. LA INSULINA HUMANA LYS{SUP,B28}PRO{SUP,B29} ES UTIL PARA EL TRATAMIENTO DE LA DIABETES. | | | | |
| 161. [946191](https://patentscope.wipo.int/search/en/detail.jsf?docId=PT108332724&recNum=161&office=&queryString=FP%3Aanalog*+AND+PA%3ALilly+AND+PD%3A%28%5B01.01.1994+to+01.01.2015%5D%29+&prevFilter=&sortOption=Pub+Date+Desc&maxRec=494) **USO DE ANALOGOS E DERIVADOS DE GLP-1 ADMINISTRADOSPERIFERICAMENTE NA REGULACAO DA OBESIDADE** | | | PT | 30.06.2003 |
| \| A61K 38/00 \| Top of Form    Bottom of Form \| \| --- \| --- \| | 97947357 | LILLY CO ELI | EFENDIC SUAD | |
| This invention relates to the use of glucagon-like peptides such as GLP-1, a GLP-1 analog, or a GLP-1 derivative in methods and compositions for reducing body weight. | | | | |
| 162. [6583111](https://patentscope.wipo.int/search/en/detail.jsf?docId=US40320122&recNum=162&office=&queryString=FP%3Aanalog*+AND+PA%3ALilly+AND+PD%3A%28%5B01.01.1994+to+01.01.2015%5D%29+&prevFilter=&sortOption=Pub+Date+Desc&maxRec=494) **Use of GLP-1 analogs and derivative adminstered peripherally in regulation of obesity** | | | US | 24.06.2003 |
| \| A61K 38/03 \| Top of Form    Bottom of Form \| \| --- \| --- \| | 09585186 | Eli Lilly and Company | DiMarchi, Richard | |
| This invention relates the use of glucagon-like peptides such as GLP-1, a GLP-1 analog, or a GLP-1 derivative in methods and compositions for reducing body weight. | | | | |
| 163. [WO/2003/050129](https://patentscope.wipo.int/search/en/detail.jsf?docId=WO2003050129&recNum=163&office=&queryString=FP%3Aanalog*+AND+PA%3ALilly+AND+PD%3A%28%5B01.01.1994+to+01.01.2015%5D%29+&prevFilter=&sortOption=Pub+Date+Desc&maxRec=494) **USE OF PHOSPHONATE NUCLEOTIDE ANALOGUE FOR TREATING HEPATITIS B VIRUS INFECTIONS** | | | WO | 19.06.2003 |
| \| [A61K 31/675](http://www.wipo.int/ipcpub/?symbol=A61K0031675000&refresh=page&viewmode=a&notes=no&headings=no&showdeleted=no) \| Top of Form    Bottom of Form \| \| --- \| --- \| | PCT/US2002/033641 | ELI LILLY AND COMPANY | WISE, Stephen, Douglas | |
| Provided are pharmaceutically acceptable compositions comprising 2-amino-9-[2-[bis(2,2,2-trifluoroethoxy)phosphonylmethoxy]ethyl]-6-(4-methoxyphenylthio) purine (LY582563) for oral administration to treat hepatitis B virus infections in infected patients. The compositions are in unit dosage form including, per unit dosage, about 2.5 to about 20 mg of the purine. The compositions are adapted for oral administration, preferably in the form of a tablet or capsule, and can be administered in single or multiple doses, provided that the total daily dose of the purine is in the range of from about 2.5 mg to about 20 mg per patient per day. These compositions are particularly advantageous for lowering the plasma HBV DNA levels, or ameliorating symptoms, conditions, or disorders caused by hepatitis B virus, of human patients with chronic HBV infection. X-15484PCT331 | | | | |
| 164. [20030104983](https://patentscope.wipo.int/search/en/detail.jsf?docId=US40080062&recNum=164&office=&queryString=FP%3Aanalog*+AND+PA%3ALilly+AND+PD%3A%28%5B01.01.1994+to+01.01.2015%5D%29+&prevFilter=&sortOption=Pub+Date+Desc&maxRec=494) **Stable insulin formulations** | | | US | 05.06.2003 |
| \| A61K 38/28 \| Top of Form    Bottom of Form \| \| --- \| --- \| | 10264176 | Eli Lilly and Company | DeFelippis Michael Rosario | |
| The present invention provides a monomeric insulin analog formulation stabilized against aggregation in which the buffering agent is either TRIS or arginine. The stable formulations of the present invention are useful for treating diabetes, and are particularly advantageous in treatment regimes requiring lengthy chemical and physical stability, such as, in continuous infusion systems. | | | | |
| 165. [1422279](https://patentscope.wipo.int/search/en/detail.jsf?docId=CN82614312&recNum=165&office=&queryString=FP%3Aanalog*+AND+PA%3ALilly+AND+PD%3A%28%5B01.01.1994+to+01.01.2015%5D%29+&prevFilter=&sortOption=Pub+Date+Desc&maxRec=494) **Selective N-acylation of A82846 glycopeptide analogs** | | | CN | 04.06.2003 |
| \| A61K 38/04 \| Top of Form    Bottom of Form \| \| --- \| --- \| | 01807738.2 | Eli Lilly and Co. | R.C. Thompson | |
| The present invention provides a process for selectively acylating an A82846A, A82846B, A82846C or PA-42867-A glycopeptide at the N1, N2 or N3 positions and the monoacylated compounds prepared therefrom. | | | | |
| 166. [1306092](https://patentscope.wipo.int/search/en/detail.jsf?docId=EP13921070&recNum=166&office=&queryString=FP%3Aanalog*+AND+PA%3ALilly+AND+PD%3A%28%5B01.01.1994+to+01.01.2015%5D%29+&prevFilter=&sortOption=Pub+Date+Desc&maxRec=494) **Use of GLP-1 and analogs administered peripherally, in regulation of obesity** | | | EP | 02.05.2003 |
| \| C07K 14/605 \| Top of Form    Bottom of Form \| \| --- \| --- \| | 02026991 | LILLY CO ELI | DIMARCHI RICHARD DENNIS | |
| This invention relates the use of glucagon-like peptides such as GLP-1, a GLP-1 analog, or a GLP-1 derivative in methods and compositions for reducing body weight | | | | |
| 167. [200300026](https://patentscope.wipo.int/search/en/detail.jsf?docId=ea95395598&recNum=167&office=&queryString=FP%3Aanalog*+AND+PA%3ALilly+AND+PD%3A%28%5B01.01.1994+to+01.01.2015%5D%29+&prevFilter=&sortOption=Pub+Date+Desc&maxRec=494) **GLUCAGON-LIKE PEPTIDE-1 ANALOGS AND USE THEREOF** | | | ea | 24.04.2003 |
| \| C07K 14/605 \| Top of Form    Bottom of Form \| \| --- \| --- \| | 200300026 | ЭЛИ ЛИЛЛИ ЭНД КОМПАНИ | Глеснер Вольфганг | |
| The invention relates to glucagon-like peptide-1 (GLP-1). The invention also relates to the use of glucagon-like peptide-1 for treating obesity, stroke, myocardial infarction, catabolic changes after surgery, or irritable bowel syndrome. The invention also relates to the use of glucagon-like peptide-1 for the preparation of a medicament for the treatment of obesity, stroke, myocardial infarction, catabolic changes after surgery, or irritable bowel syndrome. | | | | |
| 168. [6551992](https://patentscope.wipo.int/search/en/detail.jsf?docId=US40132589&recNum=168&office=&queryString=FP%3Aanalog*+AND+PA%3ALilly+AND+PD%3A%28%5B01.01.1994+to+01.01.2015%5D%29+&prevFilter=&sortOption=Pub+Date+Desc&maxRec=494) **Stable insulin formulations** | | | US | 22.04.2003 |
| \| A61K 38/00 \| Top of Form    Bottom of Form \| \| --- \| --- \| | 09450794 | Eli Lilly and Company | DeFelippis, Michael Rosario | |
| The present invention provides a monomeric insulin analog formulation stabilized against aggregation in which the buffering agent is either TRIS or arginine. The stable formulations of the present invention are useful for treating diabetes, and are particularly advantageous in treatment regimes requiring lengthy chemical and physical stability, such as, in continuous infusion systems. | | | | |
| 169. [2185113](https://patentscope.wipo.int/search/en/detail.jsf?docId=es5443339&recNum=169&office=&queryString=FP%3Aanalog*+AND+PA%3ALilly+AND+PD%3A%28%5B01.01.1994+to+01.01.2015%5D%29+&prevFilter=&sortOption=Pub+Date+Desc&maxRec=494) **FORMULACIONES ESTABLES DE INSULINA.** | | | es | 16.04.2003 |
| \| A61K 38/28 \| Top of Form    Bottom of Form \| \| --- \| --- \| | E98304661 | ELI LILLY AND COMPANY | DEFILIPPIS, MICHAEL ROSARIO | |
| LA PRESENTE INVENCION DESCRIBE UN FORMULADO DE UN ANALOGO DE LA INSULINA MONOMERICO ESTABILIZADO FRENTE A LA AGREGACION, EN EL CUAL EL AGENTE TAMPONANTE ES TRIS O ARGININA. LOS FORMULADOS ESTABLES DE LA PRESENTE INVENCION RESULTAN UTILES PARA TRATAR LA DIABETES, Y RESULTAN PARTICULARMENTE VENTAJOSOS EN REGIMENES DE TRATAMIENTO QUE REQUIEREN UNA ESTABILIDAD QUIMICA Y FISICA DURADERA, TAL COMO LOS SISTEMAS DE INFUSION CONTINUA. | | | | |
| 170. [2185113](https://patentscope.wipo.int/search/en/detail.jsf?docId=ES5443339&recNum=170&office=&queryString=FP%3Aanalog*+AND+PA%3ALilly+AND+PD%3A%28%5B01.01.1994+to+01.01.2015%5D%29+&prevFilter=&sortOption=Pub+Date+Desc&maxRec=494) **FORMULACIONES ESTABLES DE INSULINA.** | | | ES | 16.04.2003 |
| \| A61K 38/28 \| Top of Form    Bottom of Form \| \| --- \| --- \| | E98304661 | ELI LILLY AND COMPANY | DEFILIPPIS, MICHAEL ROSARIO | |
| LA PRESENTE INVENCION DESCRIBE UN FORMULADO DE UN ANALOGO DE LA INSULINA MONOMERICO ESTABILIZADO FRENTE A LA AGREGACION, EN EL CUAL EL AGENTE TAMPONANTE ES TRIS O ARGININA. LOS FORMULADOS ESTABLES DE LA PRESENTE INVENCION RESULTAN UTILES PARA TRATAR LA DIABETES, Y RESULTAN PARTICULARMENTE VENTAJOSOS EN REGIMENES DE TRATAMIENTO QUE REQUIEREN UNA ESTABILIDAD QUIMICA Y FISICA DURADERA, TAL COMO LOS SISTEMAS DE INFUSION CONTINUA. | | | | |
| 171. [PA/a/2002/002571](https://patentscope.wipo.int/search/en/detail.jsf?docId=mx97021&recNum=171&office=&queryString=FP%3Aanalog*+AND+PA%3ALilly+AND+PD%3A%28%5B01.01.1994+to+01.01.2015%5D%29+&prevFilter=&sortOption=Pub+Date+Desc&maxRec=494) **PESTICIDAL MACROLIDES** | | | mx | 04.04.2003 |
| \| A01N 43/22 \| Top of Form    Bottom of Form \| \| --- \| --- \| | PA/a/2002/002571 | DOW AGROSCIENCES LLC.* | PAUL LEWER. | |
| Macrolide compounds produced by culturing Saccharopolyspora species LW107129 (NRRL 30141) have insecticidal and acaricidal activity and are useful intermediates for preparing spinosyn analogs. | | | | |
| 172. [1294757](https://patentscope.wipo.int/search/en/detail.jsf?docId=EP13886517&recNum=172&office=&queryString=FP%3Aanalog*+AND+PA%3ALilly+AND+PD%3A%28%5B01.01.1994+to+01.01.2015%5D%29+&prevFilter=&sortOption=Pub+Date+Desc&maxRec=494) **GLUCAGON-LIKE PEPTIDE-1 ANALOGS** | | | EP | 26.03.2003 |
| \| A61K 38/00 \| Top of Form    Bottom of Form \| \| --- \| --- \| | 01939252 | LILLY CO ELI | GLAESNER WOLFGANG | |
| Disclosed are glucagon-like peptide-1 (GLP-1) compounds with modifications at one or more of the following positions: 11, 12, 16, 22, 23, 24, 25, 27, 30, 33, 34, 35, 36, or 37. Methods of treating these GLP-1 compounds are also disclosed. | | | | |
| 173. [6531448](https://patentscope.wipo.int/search/en/detail.jsf?docId=US40109138&recNum=173&office=&queryString=FP%3Aanalog*+AND+PA%3ALilly+AND+PD%3A%28%5B01.01.1994+to+01.01.2015%5D%29+&prevFilter=&sortOption=Pub+Date+Desc&maxRec=494) **Insoluble compositions for controlling blood glucose** | | | US | 11.03.2003 |
| \| C07K 7/00 \| Top of Form    Bottom of Form \| \| --- \| --- \| | 09217275 | Eli Lilly and Company | Brader, Mark Laurence | |
| The present invention relates to insoluble compositions comprising a protein selected from the group consisting of insulin, insulin analogs, and proinsulins; a derivatized protein selected from the group consisting of derivatized insulin, derivatized insulin analog, and derivatized proinsulin; a complexing compound; a hexamer-stabilizing compound; and a divalent metal cation. Formulations of the insoluble composition are suitable for both parenteral and non-parenteral delivery for treating hyperglycemia and diabetes. Microcrystal forms of the insoluble precipitate are pharmaceutically analogous to the neutral protamine Hagedorn (NPH) insulin crystal form. Surprisingly, it has been discovered that suspension formulations of such insoluble compositions possess unique and controllable dissolution properties that provide therapeutically advantageous glucodynamics compared with insulin NPH formulations. | | | | |
| 174. [WO/2003/018516](https://patentscope.wipo.int/search/en/detail.jsf?docId=WO2003018516&recNum=174&office=&queryString=FP%3Aanalog*+AND+PA%3ALilly+AND+PD%3A%28%5B01.01.1994+to+01.01.2015%5D%29+&prevFilter=&sortOption=Pub+Date+Desc&maxRec=494) **GLUCAGON-LIKE PEPTIDE-1 ANALOGS** | | | WO | 06.03.2003 |
| \| [A61K 38/00](http://www.wipo.int/ipcpub/?symbol=A61K0038000000&refresh=page&viewmode=a&notes=no&headings=no&showdeleted=no) \| Top of Form    Bottom of Form \| \| --- \| --- \| | PCT/US2002/021325 | ELI LILLY AND COMPANY | GLAESNER, Wolfgang | |
| Disclosed are glucagon-like peptide-1 (GLP-1) compounds with modifications at one or more of the following positions: 7, 8, 12, 16, 18, 19, 20, 22, 25, 27, 30, 33, and 37. Methods of treating a subject in need of GLP-1 receptor stimulation using these GLP-1 compounds are also disclosed. | | | | |
| 175. [2458371](https://patentscope.wipo.int/search/en/detail.jsf?docId=CA94104370&recNum=175&office=&queryString=FP%3Aanalog*+AND+PA%3ALilly+AND+PD%3A%28%5B01.01.1994+to+01.01.2015%5D%29+&prevFilter=&sortOption=Pub+Date+Desc&maxRec=494) **GLUCAGON-LIKE PEPTIDE-1 ANALOGS** | | | CA | 06.03.2003 |
| \| C07K 14/435 \| Top of Form    Bottom of Form \| \| --- \| --- \| | 2458371 | ELI LILLY AND COMPANY | GLAESNER, WOLFGANG | |
| Disclosed are glucagon-like peptide-1 (GLP-1) compounds with modifications at one or more of the following positions: 7, 8, 12, 16, 18, 19, 20, 22, 25, 27, 30, 33, and 37. Methods of treating a subject in need of GLP-1 receptor stimulation using these GLP-1 compounds are also disclosed. | | | | |
| 176. [WO/2003/014293](https://patentscope.wipo.int/search/en/detail.jsf?docId=WO2003014293&recNum=176&office=&queryString=FP%3Aanalog*+AND+PA%3ALilly+AND+PD%3A%28%5B01.01.1994+to+01.01.2015%5D%29+&prevFilter=&sortOption=Pub+Date+Desc&maxRec=494) **NOVEL POLYPEPTIDE ANALOGS AND FUSIONS AND THEIR METHODS OF USE** | | | WO | 20.02.2003 |
| \| [A61K 38/00](http://www.wipo.int/ipcpub/?symbol=A61K0038000000&refresh=page&viewmode=a&notes=no&headings=no&showdeleted=no) \| Top of Form    Bottom of Form \| \| --- \| --- \| | PCT/US2002/021293 | ELI LILLY AND COMPANY | HEUER, Josef, Georg | |
| Novel polypeptide analogs and fusion proteins of a transmembrane protein, LP276, are provided. Vectors and host cells directed to these polypeptides are provided. Additionally, methods of use are provided for the treatment or prevention of allergic autoimmune diseases, type 1 diabetes, inflammation, immunodeficiencies, cancers, and infectious diseases by administering an LP276 polypeptide, analogs and fusion proteins thereof to a patient in need of such therapy. | | | | |
| 177. [94180](https://patentscope.wipo.int/search/en/detail.jsf?docId=sg1341094&recNum=177&office=&queryString=FP%3Aanalog*+AND+PA%3ALilly+AND+PD%3A%28%5B01.01.1994+to+01.01.2015%5D%29+&prevFilter=&sortOption=Pub+Date+Desc&maxRec=494) **GLUCAGON-LIKE PEPTIDE-1 ANALOGS** | | | sg | 18.02.2003 |
| \| C07K 14/00 \| Top of Form    Bottom of Form \| \| --- \| --- \| | 2002079762 | ELI LILLY AND COMPANY | GLAESNER, WOLFGANG | |
|  | | | | |
| 178. [PA/a/2002/007739](https://patentscope.wipo.int/search/en/detail.jsf?docId=mx101939&recNum=178&office=&queryString=FP%3Aanalog*+AND+PA%3ALilly+AND+PD%3A%28%5B01.01.1994+to+01.01.2015%5D%29+&prevFilter=&sortOption=Pub+Date+Desc&maxRec=494) **SELECTIVE N ACYLATION OF A82846 GLYCOPEPTIDE ANALOGS** | | | mx | 17.02.2003 |
| \| A61K 38/00 \| Top of Form    Bottom of Form \| \| --- \| --- \| | PA/a/2002/007739 | ELI LILLY AND COMPANY* | ZWEIFEL, Mark, James | |
| The present invention provides a process for selectively acylating an A82846A, A82846B, A82846C or PA 42867 A glycopeptide at the N1, N2 or N3 positions and the monoacylated compounds prepared therefrom. | | | | |
| 179. [2180511](https://patentscope.wipo.int/search/en/detail.jsf?docId=es5669239&recNum=179&office=&queryString=FP%3Aanalog*+AND+PA%3ALilly+AND+PD%3A%28%5B01.01.1994+to+01.01.2015%5D%29+&prevFilter=&sortOption=Pub+Date+Desc&maxRec=494) **FORMULACIONES MONODISPERSAS DE ANALOGOS DE INSULINA ACILADOS HEXAMERICOS.** | | | es | 16.02.2003 |
| \| A61K 9/00 \| Top of Form    Bottom of Form \| \| --- \| --- \| | E00904496 | ELI LILLY AND COMPANY | NG, KINGMAN | |
| Una formulación farmacéutica adecuada para su administración a un paciente, que comprende: una solución acuosa con un pH mayor de aproximadamente 7, 9, comprendiendo la solución acuosa: a) un agente de isotonicidad; b) un derivado fenólico; c) iones de cinc; y d) un análogo de insulina humana monoacilado o una sal farmacéuticamente aceptable del análogo de insulina humana monoacilado, comprendiendo el análogo de insulina humana monoacilado o la sal farmacéuticamente aceptable del análogo de insulina humana monoacilado el polipéptido de la SEC ID NO:1 entrecruzado de manera apropiada con la SEC ID NO:2, en el que Xaa en la posición 21 de la SEC ID NO:1 se selecciona entre el grupo compuesto por Asn, Asp, Gly y Glx; Xaa en la posición 3 de la SEC ID NO:2 se selecciona entre el grupo compuesto por Asn, Asp y Glx; Xaa en la posición 28 de la SEC ID NO:2 se selecciona entre el grupo compuesto por Asp, Leu, Val, Ala y Lys acilada; y Xaa en la posición 29 de la SEC ID NO:2 se selecciona entre el grupo compuesto por Pro y Lys acilada; y además, en el que la posición 28 o la posición 29 es Lys acilada, y si la posición 28 es Lys acilada, la posición 29 no es Lys acilada, y si la posición 29 es Lys acilada, la posición 28 no es Lys acilada. | | | | |
| 180. [2180511](https://patentscope.wipo.int/search/en/detail.jsf?docId=ES5669239&recNum=180&office=&queryString=FP%3Aanalog*+AND+PA%3ALilly+AND+PD%3A%28%5B01.01.1994+to+01.01.2015%5D%29+&prevFilter=&sortOption=Pub+Date+Desc&maxRec=494) **FORMULACIONES MONODISPERSAS DE ANALOGOS DE INSULINA ACILADOS HEXAMERICOS.** | | | ES | 16.02.2003 |
| \| A61K 9/00 \| Top of Form    Bottom of Form \| \| --- \| --- \| | E00904496 | ELI LILLY AND COMPANY | NG, KINGMAN | |
| Una formulación farmacéutica adecuada para su administración a un paciente, que comprende: una solución acuosa con un pH mayor de aproximadamente 7, 9, comprendiendo la solución acuosa: a) un agente de isotonicidad; b) un derivado fenólico; c) iones de cinc; y d) un análogo de insulina humana monoacilado o una sal farmacéuticamente aceptable del análogo de insulina humana monoacilado, comprendiendo el análogo de insulina humana monoacilado o la sal farmacéuticamente aceptable del análogo de insulina humana monoacilado el polipéptido de la SEC ID NO:1 entrecruzado de manera apropiada con la SEC ID NO:2, en el que Xaa en la posición 21 de la SEC ID NO:1 se selecciona entre el grupo compuesto por Asn, Asp, Gly y Glx; Xaa en la posición 3 de la SEC ID NO:2 se selecciona entre el grupo compuesto por Asn, Asp y Glx; Xaa en la posición 28 de la SEC ID NO:2 se selecciona entre el grupo compuesto por Asp, Leu, Val, Ala y Lys acilada; y Xaa en la posición 29 de la SEC ID NO:2 se selecciona entre el grupo compuesto por Pro y Lys acilada; y además, en el que la posición 28 o la posición 29 es Lys acilada, y si la posición 28 es Lys acilada, la posición 29 no es Lys acilada, y si la posición 29 es Lys acilada, la posición 28 no es Lys acilada. | | | | |
| 181. [1283051](https://patentscope.wipo.int/search/en/detail.jsf?docId=EP13878602&recNum=181&office=&queryString=FP%3Aanalog*+AND+PA%3ALilly+AND+PD%3A%28%5B01.01.1994+to+01.01.2015%5D%29+&prevFilter=&sortOption=Pub+Date+Desc&maxRec=494) **Stable insulin formulations** | | | EP | 12.02.2003 |
| \| A61K 38/28 \| Top of Form    Bottom of Form \| \| --- \| --- \| | 02022956 | LILLY CO ELI | DEFELIPPIS MICHAEL ROSARIO | |
| The present invention provides a monomeric insulin analog formulation stabilized against aggregation in which the buffering agent is either TRIS or arginine. The stable formulations of the present invention are useful for treating diabetes, and are particularly advantageous in treatment regimes requiring lengthy chemical and physical stability, such as, in continuous infusion systems. | | | | |
| 182. [1020030011306](https://patentscope.wipo.int/search/en/detail.jsf?docId=kr476533&recNum=182&office=&queryString=FP%3Aanalog*+AND+PA%3ALilly+AND+PD%3A%28%5B01.01.1994+to+01.01.2015%5D%29+&prevFilter=&sortOption=Pub+Date+Desc&maxRec=494) **PROCESS FOR PREPARING LIPID II AND DERIVATIVES THEREOF** | | | kr | 07.02.2003 |
| \| C07H 15/00 \| Top of Form    Bottom of Form \| \| --- \| --- \| | 1020027013914 | ELI LILLY AND COMPANY | BLASZCZAK LARRY CHRIS | |
| A process is described for preparing a substrate for the transglycosylase enzymes of bacterial cell wall biosynthesis. The chemical synthesis makes available a sustainable and substantially pure source of supply of lipid II, including analogs thereof, that maybe used in the identification of new therapeutic agents capable of disrupting steps in bacterial cell wall biosynthesis.  © KIPO & WIPO 2007 | | | | |
| 183. [20030027980](https://patentscope.wipo.int/search/en/detail.jsf?docId=US39973435&recNum=183&office=&queryString=FP%3Aanalog*+AND+PA%3ALilly+AND+PD%3A%28%5B01.01.1994+to+01.01.2015%5D%29+&prevFilter=&sortOption=Pub+Date+Desc&maxRec=494) **Process for preparing lipid II** | | | US | 06.02.2003 |
| \| C07H 1/00 \| Top of Form    Bottom of Form \| \| --- \| --- \| | 09833647 | Eli Lilly and Company | Alborn, Jr. William Ernest | |
| A process is described for preparing a substrate for the transglycosylase enzymes of bacterial cell wall biosynthesis. The chemical synthesis makes available a sustainable and substantially pure source of supply of lipid II, including analogs thereof, that may be used in the identification of new therapeutic agents capable of disrupting steps in bacterial cell wall biosynthesis. | | | | |
| 184. [20030022823](https://patentscope.wipo.int/search/en/detail.jsf?docId=US39969033&recNum=184&office=&queryString=FP%3Aanalog*+AND+PA%3ALilly+AND+PD%3A%28%5B01.01.1994+to+01.01.2015%5D%29+&prevFilter=&sortOption=Pub+Date+Desc&maxRec=494) **Use of GLP-1 or analogs in treatment of myocardial infarction** | | | US | 30.01.2003 |
| \| A61K 38/17 \| Top of Form    Bottom of Form \| \| --- \| --- \| | 09834229 | Eli Lilly and Company | Efendic, Suad | |
| This invention provides a method of reducing mortality and morbidity after myocardial infarction. GLP-1, a GLP-1 analog, or a GLP-1 derivative, is administered at a dose effective to normalize blood glucose. | | | | |
| 185. [1020030009537](https://patentscope.wipo.int/search/en/detail.jsf?docId=kr474765&recNum=185&office=&queryString=FP%3Aanalog*+AND+PA%3ALilly+AND+PD%3A%28%5B01.01.1994+to+01.01.2015%5D%29+&prevFilter=&sortOption=Pub+Date+Desc&maxRec=494) **GLUCAGON-LIKE PEPTIDE-1 ANALOGS** | | | kr | 29.01.2003 |
| \| C07K 14/605 \| Top of Form    Bottom of Form \| \| --- \| --- \| | 1020027017141 | ELI LILLY AND COMPANY | GLAESNER WOLFGANG | |
| Disclosed are glucagon-like peptide-1 (GLP-1) compounds with modifications at one or more of the following positions: 11, 12, 16, 22, 23, 24, 25, 27, 30, 33, 34, 35, 36, or 37. Methods of treating these GLP- 1 compounds are also disclosed.  © KIPO & WIPO 2007 | | | | |
| 186. [RE037971](https://patentscope.wipo.int/search/en/detail.jsf?docId=US40120839&recNum=186&office=&queryString=FP%3Aanalog*+AND+PA%3ALilly+AND+PD%3A%28%5B01.01.1994+to+01.01.2015%5D%29+&prevFilter=&sortOption=Pub+Date+Desc&maxRec=494) **Selective acylation of epsilon-amino groups** | | | US | 28.01.2003 |
| \| C07K 16/00 \| Top of Form    Bottom of Form \| \| --- \| --- \| | 09351103 | Eli Lilly and Company | Baker, Jeffrey C. | |
| The present invention relates to the acylation of proteins. More particularly, the invention relates to a one-step process for selectively acylating the free ε-amino group of insulin, insulin analog, or proinsulin in the presence of a free α-amino group. | | | | |
| 187. [1276746](https://patentscope.wipo.int/search/en/detail.jsf?docId=EP13867050&recNum=187&office=&queryString=FP%3Aanalog*+AND+PA%3ALilly+AND+PD%3A%28%5B01.01.1994+to+01.01.2015%5D%29+&prevFilter=&sortOption=Pub+Date+Desc&maxRec=494) **PROCESS FOR PREPARING LIPID II AND DERIVATIVES THEREOF** | | | EP | 22.01.2003 |
| \| C07K 9/00 \| Top of Form    Bottom of Form \| \| --- \| --- \| | 01928624 | LILLY CO ELI | ALBORN WILLIAM ERNEST JR | |
| A process is described for preparing a substrate for the transglycosylase enzymes of bacterial cell wall biosynthesis. The chemical synthesis makes available a sustainable and substantially pure source of supply of lipid II, including analogs thereof, that maybe used in the identification of new therapeutic agents capable of disrupting steps in bacterial cell wall biosynthesis. | | | | |
| 188. [884053](https://patentscope.wipo.int/search/en/detail.jsf?docId=PT108198616&recNum=188&office=&queryString=FP%3Aanalog*+AND+PA%3ALilly+AND+PD%3A%28%5B01.01.1994+to+01.01.2015%5D%29+&prevFilter=&sortOption=Pub+Date+Desc&maxRec=494) **FORMULACOES DE INSULINA ESTAVEIS** | | | PT | 31.12.2002 |
| \| A61K 9/08 \| Top of Form    Bottom of Form \| \| --- \| --- \| | 98304661 | LILLY CO ELI | LI SHUN | |
| The present invention provides a monomeric insulin analog formulation stabilized against aggregation in which the buffering agent is either TRIS or arginine. The stable formulations of the present invention are useful for treating diabetes, and are particularly advantageous in treatment regimes requiring lengthy chemical and physical stability, such as, in continuous infusion systems. | | | | |
| 189. [1266897](https://patentscope.wipo.int/search/en/detail.jsf?docId=EP13848356&recNum=189&office=&queryString=FP%3Aanalog*+AND+PA%3ALilly+AND+PD%3A%28%5B01.01.1994+to+01.01.2015%5D%29+&prevFilter=&sortOption=Pub+Date+Desc&maxRec=494) **Hypoglycemic imidazoline compounds** | | | EP | 18.12.2002 |
| \| A61K 31/415 \| Top of Form    Bottom of Form \| \| --- \| --- \| | 02020546 | LILLY CO ELI | JIROUSEK MICHAEL ROBERT | |
| This invention relates to certain novel imidazoline compounds and analogues thereof, to their use for the treatment of diabetes, diabetic complications, metabolic disorders, or related diseases where impaired glucose disposal is present, to pharmaceutical compositions comprising them, and to processes for their preparation. The compounds have the following formula: wherein X is -O-, -S-, or -NR<5>-; R<5> is hydrogen, C1-8 alkyl, or an amino protecting group; R<4> is Y is -O-, -S-, or -NR<8>-; Y' is -O- or -S-; | | | | |
| 190. [2002000484](https://patentscope.wipo.int/search/en/detail.jsf?docId=sv10687217&recNum=190&office=&queryString=FP%3Aanalog*+AND+PA%3ALilly+AND+PD%3A%28%5B01.01.1994+to+01.01.2015%5D%29+&prevFilter=&sortOption=Pub+Date+Desc&maxRec=494) **ANALOGOS DEL PEPTIDO-1 SEMEJANTE A GLUCACON REF. X-13989** | | | sv | 02.12.2002 |
| \| C12N 15/12 \| Top of Form    Bottom of Form \| \| --- \| --- \| | 2001000484 | ELI LILLY AND COMPANY | MILLICAN ROHN LEE | |
| SE DESCRIBEN COMPUESTOS DE PEPTIDO-1 SEMEJANTE AL GLUCAGON (GLP-1)CON MODIFICACIONES EN UNA O MAS DE LAS SIGUIENTES POSICIONES: 11, 12, 16, 24, 25, 27, 30, 33, 34, 35, 36 O 37. TAMBIEN SE DESCRIBEN PROCEDIMIENTOS DE TRATAMIENTO DE UN SUJETO EN NECESIDAD DE ESTIMULACION DEL RECEPTOR DE GLP-1 USANDO ESTOS COMPUESTOS GLP-1 | | | | |
| 191. [WO/2002/091989](https://patentscope.wipo.int/search/en/detail.jsf?docId=WO2002091989&recNum=191&office=&queryString=FP%3Aanalog*+AND+PA%3ALilly+AND+PD%3A%28%5B01.01.1994+to+01.01.2015%5D%29+&prevFilter=&sortOption=Pub+Date+Desc&maxRec=494) **ANTIVIRAL THERAPIES USING POLYAMINE OR POLYAMINE ANALOG-AMINO ACID CONJUGATES** | | | WO | 21.11.2002 |
| \| [A61K 47/48](http://www.wipo.int/ipcpub/?symbol=A61K0047480000&refresh=page&viewmode=a&notes=no&headings=no&showdeleted=no) \| Top of Form    Bottom of Form \| \| --- \| --- \| | PCT/US2001/043887 | SLIL BIOMEDICAL CORPORATION | FRYDMAN, Benjamin | |
| Methods for treating viral diseases are provided, using conjugates in which polyamine analogs are conjugated to an amino acid. | | | | |
| 192. [WO/2002/089731](https://patentscope.wipo.int/search/en/detail.jsf?docId=WO2002089731&recNum=192&office=&queryString=FP%3Aanalog*+AND+PA%3ALilly+AND+PD%3A%28%5B01.01.1994+to+01.01.2015%5D%29+&prevFilter=&sortOption=Pub+Date+Desc&maxRec=494) **AGENTS FOR TREATMENT OF HCV AND METHODS OF USE** | | | WO | 14.11.2002 |
| \| [A61K 38/00](http://www.wipo.int/ipcpub/?symbol=A61K0038000000&refresh=page&viewmode=a&notes=no&headings=no&showdeleted=no) \| Top of Form    Bottom of Form \| \| --- \| --- \| | PCT/US2002/013951 | STANFORD UNIVERSITY | GLENN, Jeffrey, S. | |
| An amphipathic helix at the approximate N-terminus of Hepatitis C virus (HCV) nonstructural proteins mediates the association of these proteins with cytoplasmic membranes in infected cells. This association is essential for replication. Thus, assessing the ability of compounds or protocols to disrupt the association of such helices with cytoplasmic membranes permits identification of compounds and protocols which are useful in the treatment of HCV infection. Also useful in the invention are mimics, or function-disrupting ligands, of an amphipathic helix of the nonstructural proteins described herein and antibodies and fragments thereof immunoreactive with said helix. | | | | |
| 193. [PA/a/2002/000321](https://patentscope.wipo.int/search/en/detail.jsf?docId=mx94907&recNum=193&office=&queryString=FP%3Aanalog*+AND+PA%3ALilly+AND+PD%3A%28%5B01.01.1994+to+01.01.2015%5D%29+&prevFilter=&sortOption=Pub+Date+Desc&maxRec=494) **PSEUDOMYCIN N-ACYL SIDE-CHAIN ANALOGS** | | | mx | 08.11.2002 |
| \| C07K 7/06 \| Top of Form    Bottom of Form \| \| --- \| --- \| | PA/a/2002/000321 | ELI LILLY AND COMPANY* | BELVO, Matthew, David | |
| Semi-synthetic pseudomycin compounds having structure (I) are described which may be useful as antifungal agents or intermediates in the design of antifungal agents. | | | | |
| 194. [PA/a/2002/000312](https://patentscope.wipo.int/search/en/detail.jsf?docId=mx94898&recNum=194&office=&queryString=FP%3Aanalog*+AND+PA%3ALilly+AND+PD%3A%28%5B01.01.1994+to+01.01.2015%5D%29+&prevFilter=&sortOption=Pub+Date+Desc&maxRec=494) **PSEUDOMYCIN AMIDE AND ESTER ANALOGS** | | | mx | 08.11.2002 |
| \| A61K 38/08 \| Top of Form    Bottom of Form \| \| --- \| --- \| | PA/a/2002/000312 | ELI LILLY AND COMPANY* | GALKA, Christopher, Stanley | |
| Acid-modification of the aspartic acid and/or hydroxyaspartic acid units of naturally occurring or semi-synthetic pseudomycin compounds is described as well as methods of treatment against fungal activities. | | | | |
| 195. [1254164](https://patentscope.wipo.int/search/en/detail.jsf?docId=EP13825164&recNum=195&office=&queryString=FP%3Aanalog*+AND+PA%3ALilly+AND+PD%3A%28%5B01.01.1994+to+01.01.2015%5D%29+&prevFilter=&sortOption=Pub+Date+Desc&maxRec=494) **SELECTIVE N-ACYLATION OF A82846 GLYCOPEPTIDE ANALOGS** | | | EP | 06.11.2002 |
| \| A61K 38/04 \| Top of Form    Bottom of Form \| \| --- \| --- \| | 01910588 | LILLY CO ELI | THOMPSON RICHARD CRAIG | |
| The present invention provides a process for selectively acylating an A82846A, A82846B, A82846C or PA-42867-A glycopeptide at the N1, N2 or N3 positions and the monoacylated compounds prepared therefrom. | | | | |
| 196. [WO/2002/085929](https://patentscope.wipo.int/search/en/detail.jsf?docId=WO2002085929&recNum=196&office=&queryString=FP%3Aanalog*+AND+PA%3ALilly+AND+PD%3A%28%5B01.01.1994+to+01.01.2015%5D%29+&prevFilter=&sortOption=Pub+Date+Desc&maxRec=494) **PROCESS FOR PREPARING LIPID II** | | | WO | 31.10.2002 |
| \| [A61K 38/00](http://www.wipo.int/ipcpub/?symbol=A61K0038000000&refresh=page&viewmode=a&notes=no&headings=no&showdeleted=no) \| Top of Form    Bottom of Form \| \| --- \| --- \| | PCT/US2002/008266 | ELI LILLY AND COMPANY | BLASZCZAK, Larry, Chris | |
| A process is described for preparing a substrate for the transglycosylase enzymes of bacterial cell wall biosynthesis. The chemical synthesis makes available a sustainable and substantially pure source of supply of lipid II, including analogs thereof, that may be used in the identification of new therapeutic agents capable of disrupting steps in bacterial cell wall biosynthesis. | | | | |
| 197. [1146896](https://patentscope.wipo.int/search/en/detail.jsf?docId=PT108225668&recNum=197&office=&queryString=FP%3Aanalog*+AND+PA%3ALilly+AND+PD%3A%28%5B01.01.1994+to+01.01.2015%5D%29+&prevFilter=&sortOption=Pub+Date+Desc&maxRec=494) **FORMULACOES MONODISPERSAS DE ANALOGO ACILADO E HEXAMERICO INSULINA** | | | PT | 31.10.2002 |
| \| A61K 9/08 \| Top of Form    Bottom of Form \| \| --- \| --- \| | 00904496 | LILLY CO ELI | NG KINGMAN | |
| The present invention provides formulations and methods for preparing formulations containing an aqueous solution at a pH of greater than about 7.9. The aqueous solution includes an isotonicity agent, a phenolic derivative, zinc ions, and an acylated human insulin analog. More particularly, the invention relates to formulations having a pH of greater than about 7.9 that include a monoacylated human insulin analog such as an acylated des(B30) human insulin analog or an analog that comprises a native or modified human insulin A chain optionally modified at position A21, and a modified native human insulin B chain optionally modified at position B3 and modified at position B28, or at both positions B28 and B29, and that contains a lysine residue at either position B28 or B29 acylated with a fatty acid residue. The invention also provides a method for treating a patient suffering from hyperglycemia using the pharmaceutical formulations of the invention. | | | | |
| 198. [1020020073546](https://patentscope.wipo.int/search/en/detail.jsf?docId=kr441295&recNum=198&office=&queryString=FP%3Aanalog*+AND+PA%3ALilly+AND+PD%3A%28%5B01.01.1994+to+01.01.2015%5D%29+&prevFilter=&sortOption=Pub+Date+Desc&maxRec=494) **SELECTIVE N-ACYLATION OF A82846 GLYCOPEPTIDE ANALOGS** | | | kr | 26.09.2002 |
| \| C07K 9/00 \| Top of Form    Bottom of Form \| \| --- \| --- \| | 1020027010374 | ELI LILLY AND COMPANY | THOMPSON RICHARD CRAIG | |
| The present invention provides a process for selectively acylating an A82846A, A82846B, A82846C or PA-42867-A glycopeptide at the N1, N2 or N3 positions and the monoacylated compounds prepared therefrom.  © KIPO & WIPO 2007 | | | | |
| 199. [90831](https://patentscope.wipo.int/search/en/detail.jsf?docId=sg1337777&recNum=199&office=&queryString=FP%3Aanalog*+AND+PA%3ALilly+AND+PD%3A%28%5B01.01.1994+to+01.01.2015%5D%29+&prevFilter=&sortOption=Pub+Date+Desc&maxRec=494) **SELECTIVE N-ACYLATION OF A82846 GLYCOPEPTIDE ANALOGS** | | | sg | 17.09.2002 |
| \| C07K 9/00 \| Top of Form    Bottom of Form \| \| --- \| --- \| | 2002045862 | ELI LILLY AND COMPANY | THOMPSON, RICHARD, CRAIG | |
|  | | | | |
| 200. [2171839](https://patentscope.wipo.int/search/en/detail.jsf?docId=es5582654&recNum=200&office=&queryString=FP%3Aanalog*+AND+PA%3ALilly+AND+PD%3A%28%5B01.01.1994+to+01.01.2015%5D%29+&prevFilter=&sortOption=Pub+Date+Desc&maxRec=494) **ANALOGOS DE CARBAZOL COMO AGONISTAS ADRENERGICOS SELECTIVOS DE BETA3.** | | | es | 16.09.2002 |
| \| A61K 31/403 \| Top of Form    Bottom of Form \| \| --- \| --- \| | E97306613 | ELI LILLY AND COMPANY | CROWELL, THOMAS ALAN | |
| LA PRESENTE INVENCION SE SITUA EN EL CAMPO DE LA MEDICINA, EN PARTICULAR EN EL TRATAMIENTO DE LA DIABETES DE TIPO II Y LA OBESIDAD. DE FORMA MAS ESPECIFICA, LA PRESENTE INVENCION TRATA DE AGONISTAS DEL RECEPTOR BE} SUB,3} SELECTIVOS QUE RESULTAN UTILES EN EL TRATAMIENTO DE LA DIABETES DE TIPO II Y LA OBESIDAD. LA INVENCION DESCRIBE COMPUESTOS Y PROCEDIMIENTOS PARA TRATAR LA DIABETES DE TIPO II Y LA OBESIDAD, QUE INCLUYEN ADMINISTRAR A UN MAMIFERO QUE LO NECESITE LOS COMPUESTOS DE FORMULA I. | | | | |
